# Cyclin-dependent protein kinases and cell cycle regulation in biology and disease

**DOI:** 10.1038/s41392-024-02080-z

**Published:** 2025-01-13

**Authors:** Ilenia Pellarin, Alessandra Dall’Acqua, Andrea Favero, Ilenia Segatto, Valentina Rossi, Nicole Crestan, Javad Karimbayli, Barbara Belletti, Gustavo Baldassarre

**Affiliations:** Division of Molecular Oncology, Centro di Riferimento Oncologico di Aviano (CRO) IRCCS, National Cancer Institute, Aviano, Italy

**Keywords:** Epigenetics, Drug development, Diseases of the nervous system, Prognostic markers, Cardiology

## Abstract

Cyclin Dependent Kinases (CDKs) are closely connected to the regulation of cell cycle progression, having been first identified as the kinases able to drive cell division. In reality, the human genome contains 20 different CDKs, which can be divided in at least three different sub-family with different functions, mechanisms of regulation, expression patterns and subcellular localization. Most of these kinases play fundamental roles the normal physiology of eucaryotic cells; therefore, their deregulation is associated with the onset and/or progression of multiple human disease including but not limited to neoplastic and neurodegenerative conditions. Here, we describe the functions of CDKs, categorized into the three main functional groups in which they are classified, highlighting the most relevant pathways that drive their expression and functions. We then discuss the potential roles and deregulation of CDKs in human pathologies, with a particular focus on cancer, the human disease in which CDKs have been most extensively studied and explored as therapeutic targets. Finally, we discuss how CDKs inhibitors have become standard therapies in selected human cancers and propose novel ways of investigation to export their targeting from cancer to other relevant chronic diseases. We hope that the effort we made in collecting all available information on both the prominent and lesser-known CDK family members will help in identify and develop novel areas of research to improve the lives of patients affected by debilitating chronic diseases.

## Introduction

Signal transduction is heavily reliant on the activity of protein kinases which, by catalyzing protein phosphorylation, control many cellular processes, regulating intercellular communication and coordination of complex functions.

In humans, the complex regulation of processes like immunity, neurobiology, cell cycle or morphogenesis is ensure by 518 protein kinases and approximately 20 lipid kinases that compose the human kinome (∼1.7% of human genes).^1,2^ 478 of the 518 human protein kinases contain a eukaryotic protein kinase (ePK) domain, shared by all the classical kinase families.^3,4^ The ePKs are further classified into eight major groups (AGC, CAMK, CK1, CMGC, STE, TK, TKL and RGC kinase families) based on sequence similarity within this domain and can be roughly divided in those able to phosphorylate tyrosine residues and those able to phosphorylate the serine or threonine residues (also known as STK). Approximately 350 of ePKs belong to the STK group and are primarily involved in transmitting the signals within the cell, amplifying and modulating the signals received from the local microenvironment.^5^ One of the principal groups of STK, highly conserved across organisms, is the CMGC kinase family, named after the initials of its subfamily members, including cyclin-dependent kinase (CDK), mitogen-activated protein kinase (MAPK), glycogen synthase kinase (GSK) and CDC-like kinase (CLK). In total CMGC kinase family has 63 family members, including also CMGC subfamilies DYRK and SRPK.^3,6,7^

Here we focus on the roles of CDKs in physiological and pathological contexts. CDKs were first identified as the kinases able to regulate the progression through the different phases of the cell cycle in association with their activating partners cyclins. However, we now know that the 20 CDKs present in the human genome can be divided in at least three different sub-family and that, during the evolution, each CDK has acquired specific and distinct functions that extend far beyond the “simple” regulation of cell cycle progression.^8^

### The long journey from the first description of the cell to the clinical use of CDKs inhibitors

**Fig. 1:**
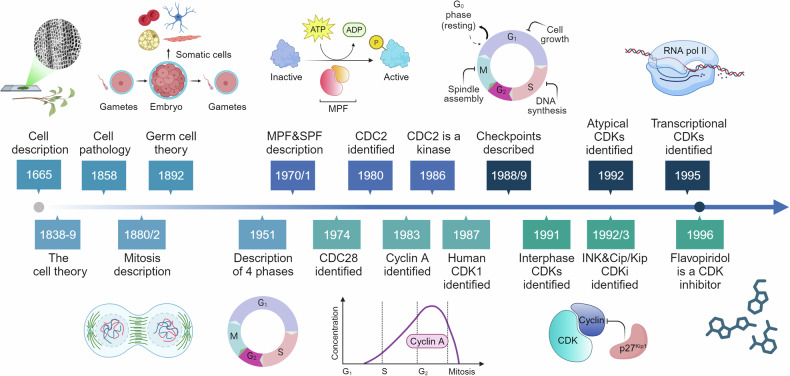
**From the cell discovery to the CDKs targeting: a subjective timeline**. Timeline of milestone events leading to the discovery, research and development related to cyclin dependent kinases (CDKs), cyclins and cell cycles regulators. In this long journey many more milestones could be identified, and this timeline represents our personal point of view. See text for references; caption of the original cork picture, described in “Micrographia” by Robert Hooke (1665), is reported in the Figure. (Adapted from “Timeline (7 segments, Horizontal), by BioRender.com (2024). Retrieved from https://app.biorender.com/biorender-templates)

The development of the first optical microscopes at the end of XVI century provided a significant boost to the study of biological and medical sciences. In 1665, physicist Robert Hooke observed, using one of the first microscopes, small, distinct structures in elderberry pith, which he named “cells” due to their resemblance to the rooms occupied by monks in convents. Hooke was actually observing dead cells and therefore only described their walls, not the nucleus or organelles^9^ (Fig. 1).

It took about 200 years of crucial observations during the XVII and XVIII centuries to confirm the presence of the cells in living organism. This culminated in the development of the “cell theory” by Matthias Schleiden and Theodor Schwann in 1838-1839, which asserted that all plants and animals are composed of cells.^10,11^ Then in 1858 Rudolf Virchow proposed that every cell arises from another pre-existing cell (“*Omnis cellula e cellula*”) and established the foundation for cell pathology as a scientific discipline.^12^

In 1880/2 advancements in staining techniques and microscopy allowed Walther Flemming and Eduard Strasburger to describe the process of mitosis, identify chromosomes, and distinguish the phases of cell division.^13,14^ In the next decade August Weisman suggested that chromosomes were the basis of heredity, proposing that genetic information is stored and transmitted by chromosomes in germ cells. He also proposed that somatic cells had limitation in their reproductive potential, which might explain ageing and natural death.^15,16^

These illuminating works on the mitotic division and chromosomes segregation were confirmed in the first 20 years of XX century but then “*the period between 1920 and 1950 was somewhat of a Dark Ages for the cell cycle*”.^17^ However, the work in these years laid the foundations for the description of the Mitosis four phases one of synthesis (S phase) and one of division (M phase) interspersed by two phases of Gap (G1 and G2) described by Howard in 1951.^18,19^

After the cell cycle was established as consisting of G1, S, G2, and M phases subsequent studies focused on understanding how transitions between these phases are regulated and led to the discovery of the M phase- and an S phase-promoting factor (MPF and SPF) which accelerate the onset of mitosis and S phase progression, respectively.^17,19^

The introduction of genetics to cell cycle studies by researchers like Hartwell and Nurse, led to the identification in yeast cells of cell division cycle (CDC) genes as necessary for cell cycle progression. Temperature sensitive lethal mutants allowed the identification of CDC28 in budding yeast as a gene necessary to control the first step of cell cycle progression in G1 phase and of CDC2 proteins in fission yeast as necessary for G2/M transition.^17,20,21^ Subsequently, using complementation approaches, the eucaryotic homologs of CDC2 (now named CDK1) has been identified and has been introduced the concept that CDKs are kinases regulated by post translational modifications.^17,22–24^ In the same years by studying the cell division of sea urchin eggs Hunt and collaborators identified one protein destroyed every time the cells divide that they proposed to name cyclin (i.e. cyclin A).^25^ At the end of 80 s, the concept that specific checkpoints exist to ensure a correct progression along the cell cycle and that damaged DNA is not transmitted to daughter cells was developed.^26^ The 90 s could marked a deeper understanding of the complex regulation of cell cycle progression, beginning with the identification of interphases cyclins and CDKs and with the key roles of TP53 and Retinoblastoma proteins as major targets and regulators of CDKs activity and cell cycle progression.^27–30^ A significant advancement was the identification of a new class of cell cycle regulating proteins: the CDK inhibitors (CKIs). CKIs inhibit the activity of CDKs by either preventing CDK4 and CDK6 binding to cyclins (INK family) or by inhibiting the activity of formed cyclin-CDK complexes (Cip/Kip family), recently reviewed by others.^31^

In the same years Harlow and collaborators, using degenerate oligonucleotides corresponding to conserved regions of CDC2 (CDK1) to amplify human sequences via PCR, identified five other genes clearly related to CDK1.^32^ Similarly, others researchers looking for the expression of CDK1-like genes in human neurons identified CDK5.^33,34^ These observations introduce the concept that CDKs form a large family of serine-threonine kinases that could have diverse functions and could be expressed in a tissue-specific manner. The CDKs family continued to expand with the identification of CDK7 (also known as a CDK Activating Kinase or CAK),^35,36^ CDK8^37^ and CDK9,^38^ now categorized as transcriptional CDKs (see next paragraphs).

CDKs play a role in various pathological conditions (as discussed in the following paragraphs). It is no surprise that their discovery stimulated the research of compounds able to inhibit their activity. This successful line of research, started with the identification that flavopiridol, a flavone with antitumor activity, that was the first in class molecule able to inhibit all cell-cycle related cyclin-CDK complexes.^39^ Although flavopiridol lacks the necessary specificity and potency to become a drug with a possible clinical use, we can say that its identification as CDK inhibitor open a new era in the treatment of several type of human cancers, as better described below (see section “CDKs as therapeutic targets”). The journey to understanding how a single cell divides into two daughter cells with high fidelity in transmitting genetic information was long and marked by the accumulation of experimental evidence, the formulation of new ideas and theories, and significant technical advancements (Fig. 1). This progress has ultimately paved the way for a new era of precision medicine, particularly in cancer treatment, where specific CDKs can now be precisely targeted for more effective patient care.

### CDK subfamilies expression and regulation in normal tissue

**Fig. 2:**
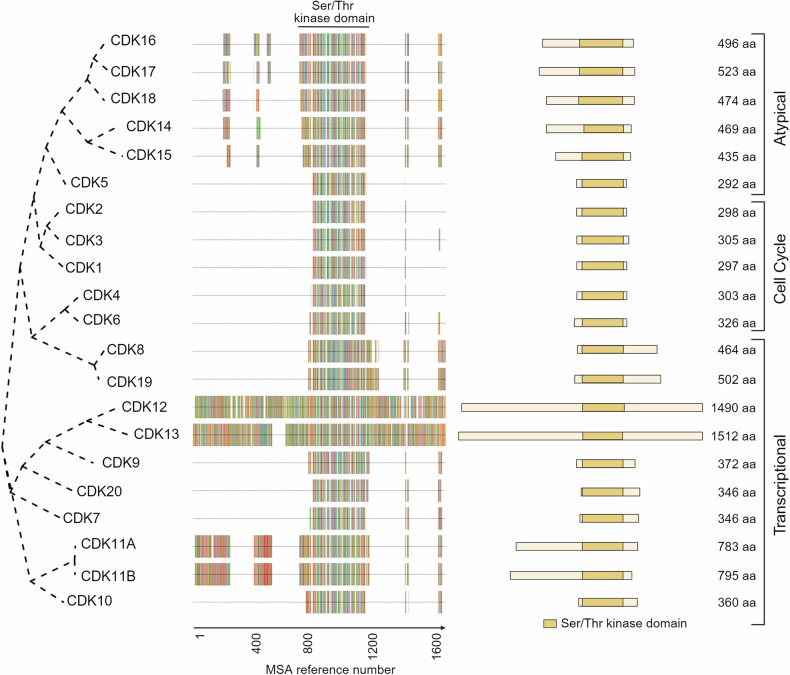
**Generation and Analysis of the Phylogenetic Tree of Human Full-Length CDKs**. The phylogenetic tree of human full-length CDKs was generated using the ggtree R package. Homology among the protein sequences was evaluated using Multiple Sequence Alignment (MSA) analysis, with CDK12 and CDK13, the longest CDKs, serving as references. The MSA reference number denotes the alignment position. Different amino acids in the protein sequences are color-coded, and gaps are introduced to properly separate conserved amino acid regions among CDKs (indicated by horizontal lines/spaces). The Serine/Threonine protein kinase domain is well conserved across all CDKs, although they show significant divergence in the lengths of their N-terminal and C-terminal extensions. On the right, a schematic representation of CDKs is provided, highlighting the kinase domain and indicating the amino acid length of each CDK. (Created with BioRender.com)

In the human genome are present 21 genes encoding 20 CDKs, named from CDK1 to CDK20, with CDK11 encompassing two isoforms, encoded by separate genes (CDK11A and CDK11B). CDKs can be classified into three phylogenetic subgroups based on their primary functions: 1) primarily involved in cell cycle regulation (CDK 1, 2, 3, 4, and 6); 2) primarily involved in transcription regulation (CDK 7, 8, 9, 10, 11, 12 and 13); and 3) atypical CDKs (CDK 5, and 14–20).^8^ Each CDK has acquired specific and distinct functions beyond merely regulating cell cycle progression (Fig. 2).

While cell cycle CDKs, with the exception of CDK3, are well studied and characterized, much less is known about the regulation and biological function of transcriptional and atypical CDKs (with the exception of CDK5). These CDKs remain part of the Dark Kinases group, which include nearly one-third of identified human kinases, and are listed in the Dark Kinase database (DKK; https://darkkinome.org). In the following sections, we will provide a detailed description of how the three CDKs subfamily are regulated by their most relevant binding partners and phosphorylation events (Table 1).

**Table 1:** Overview of CDKs-cyclin complexes and their main regulators

CDK	Cyclin/Cyclin-like	Main complex regulators	Regulators Effects on the CDK/cyclin complex
**CDK1**	Cyclin A1, A2 Cyclin B1, B2, B3	CAK complex	Activating phosphorylation
		CDC24A, CDC25B, CDC25C	Activating de-phosphorylation
		WEE1, MYT1	Inactivating phosphorylation
		p21^Cip1/Waf1/Sdi1^, p27^Kip1^	Negative regulators
**CDK2**	Cyclin E1, E2, Cyclin A1, A2	CAK complex	Activating phosphorylation
		CDC24A, CDC25B, CDC25C	Activating de-phosphorylation
		p21^Cip1/Waf1/Sdi1^, p27^Kip1^, p57^Kip2^	Negative regulators
**CDK3**	Cyclin C	CAK complex	Activating phosphorylation
		p21^Cip1/Waf1/Sdi1^	Negative regulator
**CDK4**	Cyclin D1, D2, D3	CAK complex	Activating phosphorylation
		WEE1, MYT1	Inactivating phosphorylation
		CDC25A	Activating de-phosphorylation
		p16^INK4a^, p15^INK4b^, p18^INK4c^, p19^INK4d^, p21^Cip1/Waf1/Sdi1^, p27^Kip1^	Negative regulators
**CDK5**	p25/p35/p39	CDK5RAP1 and 2	Negative regulator
**CDK6**	Cyclin D1, D2, D3	CAK complex	Activating phosphorylation
		WEE1, MYT1	Inactivating phosphorylation
		CDC25A	Activating de-phosphorylation
		p16^INK4a^, p15^INK4b^, p18^INK4c^, p19^INK4d^, p21^Cip1/Waf1/Sdi1^, p27^Kip1^	Negative regulators
**CDK7**	Cyclin H	MAT1	Required for full CDK7 activation
		CDK8-Cyclin C	Inactivating phosphorylation
**CDK8**	Cyclin C	Med12-Med13 (and paralogs)	Required for full CDK8 activation
		p21^Cip1/Waf1/Sdi1^	Stimulates CDK8 activity
**CDK9**	Cyclin T1, T2a, T2b Cyclin K	HEXIM1-2 7SK snRNA	Negative regulators
		BRD4	Positive regulator
		CAK complex	Activating phosphorylation
		PP2A phosphatase	Activating de-phosphorylation
		p300 HAT	Acetylation of cyclin T1 (activation of P-TEFb complex)
**CDK10**	Cyclin M	-	-
**CDK11**	Cyclin L	-	-
**CDK12**	Cyclin K	CAK complex	Activating phosphorylation
**CDK13**	Cyclin K	CAK complex	Activating phosphorylation
**CDK14**	Cyclin Y Cyclin D3	14-3-3	Increase of CDK14-Cyclin Y binding
		p21^Cip1/Waf1/Sdi1^	Negative regulator
**CDK15**	-	-	-
**CDK16**	Cyclin Y Cyclin Y L1	PKA	Increase of CDK16 kinase activity
		14-3-3	Increase of CDK16 kinase activity
**CDK17**	-	-	-
**CDK18**	Cyclin A2 Cyclin E1	PKA	Increase of CDK18 kinase activity
**CDK19**	Cyclin C	Med12-Med13 (and paralogs)	Required for full CDK19 activation
**CDK20**	Cyclin H	-	-

The table summarizes the CDKs and the most relevant associated cyclins and complex regulators, based on available literature. Only the interactions confirmed in multiple reports are listed. See main text for the appropriate references

*CAK* CDKs Activating Kinases, *HAT* Histone Acetyl Transferase, *PKA* Protein Kinase A. *PP2A* Protein Phosphatase 2A

Among understudied kinases, there is also the related family of CDKL (CDK like Kinases), composed of five members CDKL1 to CDKL5 which have high sequence similarity to CDKs and encode for a cyclin binding domain, although there are no evidence of their interaction with cyclins (reviewed in Ref. ^40^). The members of CDKL family will not be discussed here.

#### Cell cycle CDKs

**Fig. 3:**
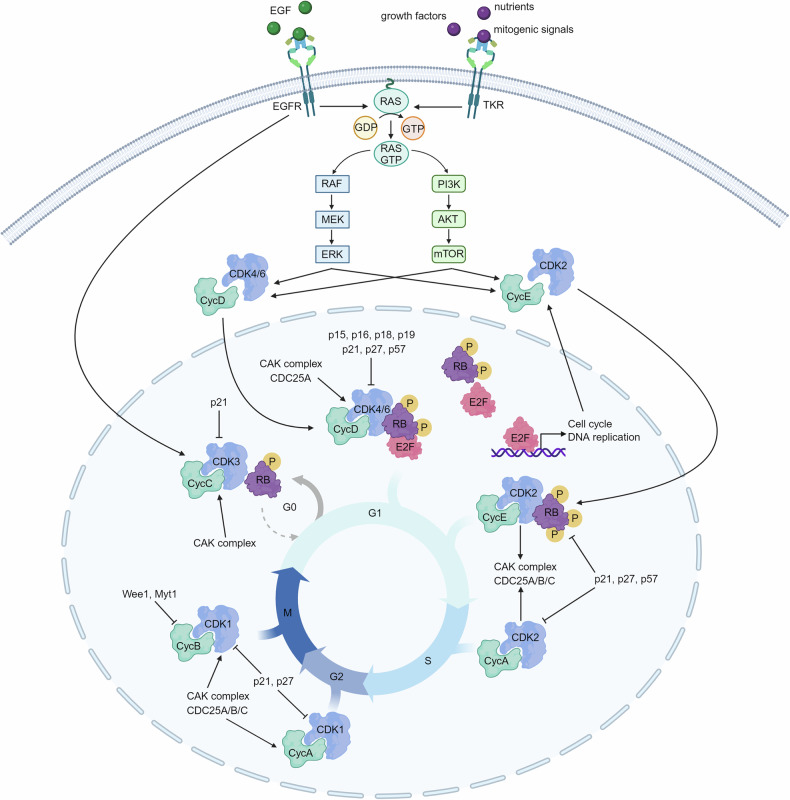
**Cell Cycle Progression via EGFR and Receptor Tyrosine Kinase Signaling**. EGFR stimulation promotes the activation of the CycC-CDK3 complex, enabling the cell to exit quiescence (G0) and enter the G1 phase by priming RB phosphorylation. Activation of various Receptor Tyrosine Kinases (RTKs) can similarly promote G1 progression through signal cascades involving RAS-GTP and its downstream RAF/MEK/ERK and PI3K/AKT/mTOR pathways. These signaling cascades lead to the formation and activation of CycD-CDK4/6 complexes and their translocation to the nucleus. In the nucleus, CycD-CDK4/6 complexes further phosphorylate RB. The inhibition of RB allows the accumulation of E2F on DNA, promoting the transcription of genes essential for cell cycle progression and DNA replication. Subsequently, the activation of the CycE-CDK2 complex drives the transition from G1 to S phase by hyper-phosphorylating RB, enabling cell cycle progression independently of growth factor stimuli (bypassing the restriction point). The accumulation of CycA and the displacement of CycE from the CycE-CDK2 complex facilitate the formation of the CycA-CDK2 complex, which drives S phase entry, progression, and DNA synthesis. Following faithful DNA replication, the CycA-CDK1 complex triggers entry into mitosis. This is followed by the formation and activation of the CycB-CDK1 complex, which is necessary for the completion of proper cell division. The roles of activating proteins (such as CAK and CDC25A/B/C) and inhibitory proteins (such as CDK inhibitors, WEE1, and MYT1) on specific cyclin-CDK complexes are indicated by black arrows. Abbreviations used: RTKs Receptor Tyrosine Kinase, CAK complex CDK Activating Kinase complex, CycA cyclin A, CycB cyclin B, CycC cyclin C, CycD cyclin D, CycE cyclin E, CDC25 Cell Division Cycle 25. (Adapted from “Cell Cycle Checkpoints”, “RAS Pathway”, by BioRender.com (2024)

The eukaryotic cell cycle (Fig. 3) can be defined the universal process by which cells correctly divide into two daughter cells ensuring that no damaged DNA is transmitted. This process underlies the growth and development of all living organs.^17^

Cell cycle goal is to guarantee that DNA faithfully replicates once during S phase (DNA Synthesis phase) and that identical chromosomes copies segregate equally into two dividing cells during mitosis (or M phase).^17^ Progression along the different phases of the cell cycle is strictly monitored to ensure that S phase is completed before mitosis begins. There are two Gap phases in somatic cell cycle: G1 separating the M and S phase, and G2 separating the S and M phase- absent in early embryonic cell cycles. During G1 cells respond to extracellular stimuli that make the cell decide whether to replicate its DNA and divide or to exit the cell cycle and enter a quiescent state (G0). Once entered into the mitotic cell cycle and decide to replicate their DNA, cells are irreversibly committed to complete the cycle and this decision is virtually taken by overstepping the so called “restriction point” or G1 checkpoint, set in late G1.^41^ While continuous mitogenic stimulation is necessary for cells to cross the restriction point, once this checkpoint is left behind, the cell cycle takes place in a mitogen independent manner. Similarly anti-proliferative signals can exert their effect only if they target the cell in early G1 before passing through the restriction point. Yet, if damaged or incompletely replicated DNA is not sufficiently repaired cells block the cell cycle in G2 and do not initiate mitosis thanks to the activation of the G2/M checkpoint, linking DNA damage response to cell cycle progression.^42,43^

To ensure that cell cycle progress and arrest as necessary, cell cycle CDKs, including CDK1, CDK2, CDK3, CDK4 and CDK6 (reviewed in Ref. ^8^), are usually constitutively expressed in quiescent cells as inactive form and, their activity tightly regulated by a four-level activating cascade that include environmental factors, cyclins expression, CDKs phosphorylations and CDKs inactivation (Fig. 3). Schematically:

I. Cell decision to progress through the cell cycle and divide is based on its ability to sense and transduce environmental signals like nutrients, mitogens, or cytostatic factors. For instance, growth factor stimulation (e.g., EGF) activates the RAS-RAF-MEK-ERK1/2 pathway, a crucial signal transduction cascade that drives cells from a quiescent state (G0) into the cell cycle, mainly by regulating cyclins and CKI expression. Once cells commit to entering the cycle, they are irreversibly committed to completing it, regardless of the continued presence of these external signals.^44^
II. Each cell cycle phase is characterized by the expression of specific cyclins, which regulate CDK activation. Cyclin levels fluctuate throughout the cycle, with their synthesis and degradation (known as “cyclin waves”) tightly controlling kinase activity in a time-dependent manner.^8^ Cyclins not only activate CDKs but may also influence CDK substrate specificity. Ten cyclins, grouped into four types (A-, B-, D-, and E-type), associate with cell cycle CDKs.^8,45^
III. The final step in fully activating cyclin-CDK complexes involves phosphorylation by the CAK complex (CDK Activating Complex) in the nucleus. The CAK complex is a heterodimer formed by the catalytic subunit CDK7, the regulatory subunit cyclin H and the assembly factor MAT1. Several evidences suggest that cyclin binding is necessary for CAK phosphorylation, as it induces conformational changes in the CDK, exposing the phosphorylation site within the T-loop. This allows way, the CAK complex to phosphorylate and activate CDKs (CDK1 at T161, CDK2 at T160, CDK3 at T161, CDK4 at T172, and CDK6 at T177).^35,46^
IV. CDK inactivation occurs through phosphorylation on specific sites or by binding with CDK inhibitors (CKIs). Phosphorylation of threonine and tyrosine residues in the G-loop (T14 and Y15), by WEE1 and MYT1 inhibit kinase activity, preventing cell cycle progression, particularly in response to DNA damage. This phosphorylation does not significantly alter CDK structure but reduces its substrates affinity.^8,47,48^

In the second case two families of CKIs have been defined. INK4 proteins (inhibitors of CDK4), including p16^INK4a^, p15^INK4b^, p18^INK4c^ and p19^INK4d^, which specifically inhibit CDK4 and CDK6 and the CIP/KIP family (i.e. p21^Cip1/Waf1/Sdi1^, p27^Kip1^, and p57^Kip2^), which bind to both cyclins and CDKs through a conserved N-terminal domain, blocking their catalytical activity.^31^

##### Entering into the cell cycle: Role of CDK4 and CDK6

The G1 kinases CDK4 (303 aa, 12q14.1) and CDK6 (326 aa, 7q21.2) share 71% sequence homology and were first described by Meyerson et al. as members of the Cdc2 (also known as CDK1)-related kinase family, along with CDK2 and CDK3.^32,49^ Despite their phylogenetic distinction from canonical CDKs (CDK1 and CDK2) due to different substrate specificities, CDK4 and CDK6 play crucial roles in cell cycle regulation. Their expression is predominant in the intestinal tract, with CDK4 also present in gynecological tissues such as the ovary and endometrium, and CDK6 in lymphoid tissues, breast, and testis (Human Protein Atlas data).

In normal cells, CDK4 and CDK6 activation requires binding with D-type cyclins (D1, D2, and D3),^50^ which are multifunctional regulators induced by extracellular mitogenic signals, like the activation of growth factor receptors and integrin-derived adhesion signaling.^44^

The assembly of cyclin D-CDK4/6 complexes is carefully and temporally regulated during various stages of cell development and differentiation. This regulation varies across cell types, as seen, in hematopoietic, neuronal, and murine embryonic stem cells (mESCs). For instance, cyclin D2 and D3 are highly expressed in most hematopoietic lineages, while cyclin D1 expression is high in embryonic tissue and persist in adult tissue (e.g. in the adult mouse brain). Additionally, cyclin D1 primarily associates with CDK4 in undifferentiated, exponentially growing mESCs but this binding is abolished in G1 arrested mESCs.^51,52^

CDK4 and CDK6 activation requires phosphorylation by the trimeric CAK complex and dephosphorylation of inhibitory phosphates by CDC25 family members. These kinases enter the cell cycle through phosphorylation at T172 (CDK4) and T177 (CDK6), either as monomers or when complexed with cyclin D and CKIs.^53^ During the G1 phase, CDC25A removes inhibitory phosphorylation fully activating CDK4 and CDK6. D-type cyclins direct the nuclear translocation of CDK4/6 to phosphorylate retinoblastoma (RB) tumor suppressor family members RB/p105 and RB2/p130, inactivating them.^54^

CDK4/6 initially phosphorylates RB in G1, with cyclin E-CDK2 driving hyperphosphorylation at the G1/S transition, fully inactivating RB’s nuclear functions.^55^ Phosphorylation on S807/S811 by CDK4/6, unlike most other sites in RB (i.e. T821, T826), does not seem to cause a structural change nor inhibition of E2F binding, however mutation on these sites prevents the efficient phosphorylation of RB in vivo, sustaining the idea that priming at these sites promotes an intermolecular association to facilitate further phosphorylation.^53^ Overall, the most accepted model of cell cycle progression in G1 (Fig. 3) suggests that CDK4/6 activity is sufficient to induce RB hyperphosphorylation and E2F activation, while cyclin E/A-CDK2 maintains hyperphosphorylation in the S phase.

Genetic modified mouse models (GEMM) studies have been crucial in understanding the role of CDKs in mammalian development and adult organs physiology. Studies involving CDK4 and CDK6 knockout mice have revealed their redundant and unique non-overlapping functions. Combined ablation of these genes results in specific phenotypes, primarily affecting liver and cardiac tissue.^56^ CDK4 knockout embryos develop normally, suggesting that CDK4 is not essential for proper mouse development but preferentially affects overall postnatal growth.^57^ Indeed, they exhibit hypoplasia of various organs, decreased body weight, polyuria, polydipsia, diabetes mellitus, and pancreatic islet degeneration, suggesting a critical role in postnatal proliferation and maintenance of endocrine cells.^57^ CDK4 depletion also impairs adipocyte function and female fertility, affecting glucose metabolism and the hypothalamic-pituitary axis.^58,59^ CDK4-deficient MEFs exhibit reduced RB phosphorylation, delaying S-phase entry and extending G1 phase duration.^60^ On the contrary, MEFs harboring the CDK4 activating mutation R24C, which abrogates p16^INK4^ inhibitory activity, exhibit decrease doubling times, with a slightly higher percentage of cells in S and G2/M phases, and fail to undergo senescence. Accordingly, CDK4-R24C knock-in mice have increased adipogenic potential, with increased weight of 5-10% compared to control littermates.^57,61^

CDK6 deletion leads to hematopoietic defects, including thymus and spleen hypoplasia, and reduced megakaryocyte and erythrocyte numbers.^56^ Combined CDK4/6 knockout results in late embryonic or postnatal lethality due to severe anemia, highlighting their importance in hematopoietic lineage development.^56^ CDK6 is predominantly localized in the cytoplasm, with a smaller nuclear fraction, indicating additional functions beyond cell cycle progression, such as rapid cell cycle entry post-reactivation in CD8 memory cells and cytoskeletal rearrangement in astrocytes.^62^

More recently, CDK6 was shown to profoundly reduce thymic cellularity and development, interfering with the proliferative and survival signals activated by NOTCH and AKT pathways.^63^

Altogether, above mentioned studies revealed that many normal non-transformed mammalian cell types can proliferate without any cyclin D-CDK4/6 activity, suggesting compensatory role of other cyclin-CDKs complexes or pathways.^56,64^

Although the canonical role of cyclin D and CDK4/6 as essential drivers of cell cycle entry and progression has been firmly established, research carried out over the past two decades provides increasing evidence for additional non canonical functions of these proteins. Among the different activities ascribed to CDK4 and 6 complexes exhaustively reviewed by Hydbring and colleagues, either dependently or independently from their kinase activity,^52^ we would highlight their important contribution in regulating FOXO transcription factors. Both CDK4 and 6 controls the activity of FOXM1 thereby regulating G2/M transition and cellular senescence.^65^ Conversely, CDK6 but not CDK4 could phosphorylate FOXO3a eventually regulating DNA damage response to chemotherapy.^66^ These observations open the way to differently think to the possible roles of CDK4/6 inhibitors in the therapies of human diseases.

As mentioned above, cell cycle entry regulated by CDK4 and CDK6 activity is tightly regulated by environmental stimuli. Most of these stimuli impact on the regulation of cyclin Ds or CKIs expression, that in turn modulates complex formation and activity. Sensing these stimuli, the cell could regulate cyclin Ds expression either transcriptionally^67^ or post-transcriptional level by ubiquitylation and proteasome degradation^68^ through the activation of ERK/MAPK and the PI3K signaling pathways that act downstream of RAS. The same pathways also regulates the expression of CKIs, especially p27^Kip1^ (hereafter referred as p27), therefore coordinating G1/S phase transition.^69^ Recently, it has been proposed that a consistent degree of cross regulation exists between cyclin and CDK inhibitors in response to extracellular stimuli, explaining the complexity of feed-forward loop regulation in the control of G1 phase progression in normal cells.^70^

Antimitogenic signals more prominently act on CDK inhibitors. For simplicity here we will refer to other excellent reviews reporting how CDK inhibitors of INK4 family, that specifically bind CDK4 and CDK6 in the non-catalytical region, are mainly regulated at transcriptional level.^71,72^ Conversely, CIP/KIP family inhibitors, had multifaced roles and could act both as activator or repressor of cyclin D-CDK4/6 kinase activity depending their status and localization. In sensing extracellular stimuli, their expression is more regulated at post-transcriptional levels although transcriptional regulation has been also observed.^69,71^

##### The G1-S phase transition, the S phase progression and beyond: role of CDK2

CDK2 (298 aa, 12q13.2) is a ubiquitous protein predominantly expressed in the gastrointestinal tract, lymphoid tissues, placenta, and testis (data from the Human Protein Atlas). Along with CDK3, it is part of the CDK1-related kinase family, although CDK2 and CDK3 do not share the same mitotic functions as CDK1. CDK2 orchestrates several cell cycle events, such as the G1 to S transition, DNA replication, and progression into G2, and is essential for meiosis but dispensable for mitosis.^49,73,74^

CDK2 could bind cyclins of the E (cyclins E1/2) and A (cyclins A1/2) families.^75^ Cyclin E1 and E2 are expressed in proliferating cells with overlapping functions in CDK2 activation.^76^ Conversely, cyclin A1 and A2 are expressed in germinal and somatic cells, respectively.^77^ The cyclin E-CDK2 and cyclin A-CDK2 complexes are formed sequentially during cell cycle progression and control not only G1/S phase transition and progression through S and G2 phases but are also crucial for activating the pre-replication complex and preventing DNA re-duplication.^76^

CDK2 activation follows a four-level cascade schematically described above. During the G1 phase, the cyclin D-CDK4/6 complex phosphorylates RB family members, promoting the expression of CDK2 activators like cyclins E and A. Upon cyclin A/E -CDK2 complex formation, the CAK trimeric complex phosphorylates CDK2 Thr-160 in the activation loop, exposed by the conformational change induced by cyclin binding.^78^ Proper orientation of the activation segment and binding to specific cyclins (A or E) confer substrate specificity, highlighting CDK2’s widespread role in cell cycle progression (Fig. 3).

Cyclin E’s role is primarily restricted to late G1 and entry into the S phase. The most relevant cyclin E-CDK2 cell cycle substrates are RB proteins, the CIP/KIP inhibitor p27, and Histone H1, briefly discussed below. The cyclin E-CDK2 complex phosphorylates RB, activating an autoregulatory positive feedback loop that promotes cyclin E expression and initiates a transcriptional program crucial for DNA synthesis.^79^ The cyclin E-CDK2 complex phosphorylates all RB family members, but the consensus site on RB is distinct from that of cyclin D-CDK4/6, with only partial overlap between CDK4/6 and CDK2 phosphorylation sites.^80^ This step is vital for completely inactivating RB proteins, allowing the cell cycle to proceed independently of cyclin D-CDK4/6 activity.^81,82^

Cyclin E-CDK2 phosphorylates p27 to initiate its ubiquitin-dependent degradation via the proteasome removing the barrier that restrains the activity of cyclin E and A containing CDK complexes. This allow to reverses the block of cell cycle ensured by p27 expression, allowing G1/S phase transition and progression through S phase^69,83^ (Fig. 3).

Phosphorylation of Histone H1 by CDK2-cyclin E is conversely necessary to relaxes chromatin structure and to stimulate DNA transcription.^84^

Another fundamental role of CDK2 in complex with cyclin E and cyclin A is the regulation of DNA replication during the S phase. Cyclin E- CDK2 complex regulates beginning of DNA replication during S phase by phosphorylating CDC6, a key component of DNA pre-replication complexes, and cooperates with DBF4-dependent CDC7-kinase (DDK) to recruit DNA replication proteins.^85,86^

During DNA replication, CDK2 binds to cyclin E and A, ensuring efficient initiation and elongation of DNA synthesis and inactivating pre-replication complexes to prevent reduplication. Cyclin A-CDK2 phosphorylates proteins such as RPA, DNA polymerase α, DNA polymerase δ, and PCNA in early S phase, sharing this activity with cyclin E-CDK2 or cyclin B-CDK1 complexes.^87^ Later in S phase, cyclin A-CDK2 drives additional factors like CDC6, MCM5, and ORC1 to chromatin or centrioles, prolonging centrosome reduplication inhibition. At the end of S phase, cyclin A-CDK2-mediated E2F phosphorylation impairs E2F’s DNA binding, inactivating it in a feedback loop that ensures transcription control post-DNA replication.^87,88^

Although cyclin A-CDK2’s functions in the S phase are well documented, its involvement in the G2 phase is less understood. Cyclin A-CDK2 promotes cyclin B-CDK1 complex activation by stimulating PLK1 through BORA phosphorylation, inducing CDC25 proteins. Cyclin A-CDK2 thus drives CDC25B and CDC25C expression, promoting CDK1 dephosphorylation and activation in mitosis.^89,90^ During mitosis, cyclin A is degraded after nuclear envelope breakdown and before metaphase by the Anaphase-Promoting Complex/Cyclosome (APC/C). Moreover, CDK2 in complex with cyclin B, increases RB and Lamin B1 protein phosphorylation during S and M phases contributing to suppress RNA polymerase II activity during mitosis to block RNA replication^91^ (Fig. 3).

CDK2, as noted for CDK4/6, targets non-cell cycle-associated substrates that could contribute to regulate cell cycle progression. For example, FOXM1 phosphorylation by cyclin E-CDK2 and cyclin A-CDK2 activates its transcriptional activity, linking early cell cycle regulation to mitosis control.^92^ Similarly, CDK2 phosphorylates SMAD3, inhibiting its transcriptional activity, reducing p15^INK4^ levels, and indirectly promoting c-MYC transcription, facilitating G1 to S phase progression.^93^

A less expected role for CDK2 in the control in DNA damage response and repair came from the studies of knockout mice and derived MEF cells. CDK2 targets several DNA repair proteins involved in HR and NHEJ pathways, such as BRCA1, BRCA2, p53, and Ku70, contributing to ensure that DNA repair is tightly linked to cell cycle progression.^94^

CDK2, CDK4, and CDK6 are critical for cell development, as demonstrated by combined gene ablation studies.^56,74,77,95^ CDK2 knockout mice are viable and develop normally, although embryos exhibit delayed S phase entry, similar to CDK4 knockout mice, suggesting a role in proper DNA replication timing.^56,77,96^ Cyclin A1 is essential in somatic cells while cyclin A2 is expressed in early embryo stages and meiosis, highlighting their distinct yet complementary roles.^97^ Accordingly, cyclin A2’s ability to bind and activate CDK1 likely compensates for CDK2 loss, has been proposed as a mechanism embryonic viability in CDK2 null mice.^97^

Adult CDK2 KO mice exhibit a specific phenotype primarily characterized by loss of body weight and sterility in both males and females, with complete penetrance, suggesting a crucial role in gametogenesis and meiosis.^74,77^ Similar effects are observed in cyclin E knockout mice, which partially resemble the phenotype of CDK2-deficient mice, supporting the involvement of the cyclin E-CDK2 complex in spermatogenesis. Mice harboring two kinase-dead mutant forms of CDK2 (D145N and T160A) also showed abnormalities in reproductive organs, impaired function, and defects in meiotic cell division, leading to infertility.^98^

Studies on CDK4 and CDK2 double mutants indicate that the loss of both kinases does not affect embryonic development or organogenesis in newborns. However, these mice die shortly after birth, primarily due to decreased cardiomyocyte proliferation, which leads to heart failure. Experiments on mouse embryonic fibroblasts (MEFs) with the same double mutant background provide further evidence of the dispensability of CDK4 and CDK2 in cell cycle progression, as these fibroblasts become immortal with a normal proliferation rate.^96^

Despite CDK2’s dispensability during embryonic development, its activity is higher in embryonic cells than in somatic cells, particularly when complexed with cyclin E, and decreases during the dissolution of pluripotency. The cyclin E-CDK2 complex phosphorylates several pluripotency regulators, including NANOG, OCT4, and SOX2, preventing their degradation. Consistent with this, abrogation of CDK2 activity results in loss of pluripotency and initiation of cell differentiation.^76,99,100^

Based on the above-described multiple activities of CDK2-contining complexes, observed in vitro and in vivo, is not surprisingly that CDK2 expression and activity is tightly controlled by non-canonical mechanisms other than the activation of the cyclin D-CDK4/6-RB pathway, which induces the transcription of cyclin E and A partners and activator of CDK2 in the G1/S transition.^55^ ERK pathway inhibition results in abrogation of cyclin E-CDK2 nuclear translocation, downregulation of its phosphorylation at the Thr160 site, and thus inhibition of its activity, suggesting a role for RAS/MEK/ERK signaling in CDK2 activation.^101,102^ Additionally, AKT binds to and phosphorylates CDK2. This phosphorylation occurs at a specific stage of the cell cycle, during late S and G2 phases, and results in the transient relocation of CDK2 to the cytoplasm, which is required for normal cell cycle progression from S to G2 phase.^103^

The CDC25A has traditionally been assigned the role of promoting entry into the S phase by dephosphorylating and activating the CDK2-cyclin E and CDK2-cyclin A complexes.^104^ However, studies on CDC25B and CDC25C inhibition suggest that all three members of the CDC25 family are involved in the control of CDK2 activity and S-phase initiation.^105,106^

As mentioned above, the kinase activity of CDK is negatively controlled mainly through binding with CIP/KIP family members, with some specific difference among the three members of the family. p21^Cip1/Waf1/Sdi1^ (hereafter referred as p21), mainly involved in blocking the cell cycle upon DNA damage (as it is a transcriptional target of p53), promotes tumor suppressor activities by inhibiting CDK2 in response to various cellular and environmental signals. It exerts its inhibitory activity by disrupting the interaction between CDKs and their substrates, such as RB proteins and CDC25 phosphatases.^107^

p27, mainly triggered by antiproliferative stimuli, is likely the most specific CDK2 inhibitor that preferentially bind CDK2 when is already bound to its cognate cyclins although it could bind also isolated cyclin A/E and CDK2. p27 interaction with cyclin A/E-CDK2 complexes, induced a conformational change near the CDK2 catalytic cleft that impairs ATP binding and CDK2 phosphorylation by the CAK complex. As mentioned above, p27 not only is the preferential inhibitor of CDK2 complexes but also one of its preferential substrates making the regulation of p27-CDK2 interaction a central point in the G1 to S phase transition^69,108^ (Fig. 3).

Finally, p57^Kip2^, distinguished from p21 and p27 by its unique structure, is expressed in a tissue-specific manner and preferentially binds to cyclin E-CDK2, acting mostly in the G1 phase.^109^

##### The G2/M phase transition and the progression through Mitosis: Role of CDK1

CDK1 (297 amino acids, located at 10q21.2) is a ubiquitous protein found in both the nucleus and cytoplasm, expressed in various tissues such as the testis, respiratory tract, gastrointestinal tract, lymphoid tissues, and female reproductive organs (data from Human Protein Atlas). CDK1, initially discovered in budding and fission yeast is the master mitotic kinase, activated at the end of interphase to promote the onset of mitosis. It interacts with cyclins A and B, forming substrate-specific complexes that function at specific cell cycle phases.

The cyclin A and B families are the mainly activating partners of CDK1. Cyclin A, particularly cyclin A2, is highly expressed in the nucleus of somatic cells and activates CDK1 during the S and G2 phases.^110^ Cyclin B family members (B1, B2, and B3) are mostly expressed in the cytoplasm, with cyclins B1 and B2 binding to CDK1 during mitosis. Cyclin B1 localizes near microtubules, cyclin B2 associates with the Golgi apparatus. While cyclin B1 and B2 are ubiquitous, cyclin B3 expression is restricted to developing germ cells and adult testis.^111^

Early studies indicated that cyclin A is a more potent activator of CDK1 than cyclin B, likely because the cyclin A-CDK1 complex is necessary to activate cyclin B-CDK1 at the beginning of mitosis.^112^ During late S/G2, cyclin A binds and activates CDK1 to regulate mitosis entry, after which the complex is degraded via the ubiquitin-proteasome system.^112,113^ Conversely, while cyclin B accumulates in G2 and persists until mid-mitosis, cyclin A-CDK1 inhibits DNA polymerase α primase in late S phase to prevent DNA re-replication.^112,114^

Before mitosis, CDK1 binds cyclin B following phosphorylation by the CAK complex on the T161 residue, promoting kinase activation.^115^ CDK1 also binds to WEE1 and MYT1, which inhibit CDK1 through phosphorylation of T14 and Y15, blocking ATP binding.^116,117^ The trimeric cyclin B-CDK1-WEE1 complex maintains low CDK1 activity in G2 until CDC25 family members remove inhibitory phosphorylations, triggering mitosis entry.^118^ Cyclin B-CDK1 also activates CDK7 in G2, sustaining CDK1 phosphorylation on T161 through a positive feedback loop.^119^

Fully activation of cyclin B-CDK1 complexes starts during prophase, with cyclin B accumulating at the centrosome to activate CDK1 in a cyclin A-independent manner. These complexes then translocate to the nucleus, where WEE1 is inhibited and CDC25 phosphatases are activated to complete mitosis entry.^118^ All three CDC25 family members regulate cyclin B-CDK1 activation, with CDC25A involved in both G1/S and G2/M transitions, and CDC25B and CDC25C mainly functioning in mitosis entry (Fig. 3).^120^

Quantitative mass spectrometry and chemical inhibition studies have identified over 300 potential CDK1 substrates, with gene ontology analysis revealing a strong enrichment in proteins related to mitosis and cell cycle regulation.^121^

As a detailed explanation of each of CDK1 targets is beyond the scope of this review, only the fundamental pathways and proteins targeted by CDK1 will be reported here.

At the beginning of mitosis, CDK1 promotes chromosome condensation, spindle formation, and chromosome attachment to the mitotic spindle also preventing dissolution of chromosome cohesion. CDK1 also regulates spindle pole body (SPB, analog of mammalian centrosome) duplication and separation and stabilizes mitotic spindle positioning and elongation. During cytokinesis, CDK1 phosphorylates proteins necessary for Golgi vesicle fragmentation and separation.^122,123^

Inactivation of mitotic CDK1 is essential for exiting mitosis, including spindle disassembly, chromosome de-condensation, cytokinesis, nuclear envelope reformation, transcription reactivation, and Golgi apparatus rebuilding. Inhibition of CDK1 in late mitosis occurs through SIC1-mediated cyclin B ubiquitination and degradation by the APC/C complex, and through WEE1 and MYT1-mediated phosphorylation.^124,125^

Besides its critical role in controlling mitosis progression ensuring chromosome and genome stability, cyclin B1-CDK1 complex participates in several other necessary cellular function. By relocating to mitochondria, it enhances mitochondrial bioenergetics and phosphorylates mitochondrial proteins to increase respiration.^126^ By binding and phosphorylating BRCA2, preventing RAD51 recruitment to DNA damage sites this complex also plays a critical role in double-strand break (DSB) repair.^127,128^ Finally, CDK1 phosphorylates the catalytic subunit of PRC2, involved in gene expression regulation, and modulates the activity of RNA polymerase II and III, thus having a role also in transcription regulation.^129^

Taking into the account these multiple roles of CDK1 is not surprising that ablation of CDK1 in mice is not compatible with life early during embryogenesis since mice harboring an inactive mutant form of CDK1 did not yield homozygous mutant CDK1 nor midgestation embryos (E10.5-E13.5); moreover, no embryos at morula stages (E2.5) or 2-4 step of cell division (E1.5) were detected.^130^ These results clearly indicate that CDK1 is a kinase essential for proper cell survival and development. Similar evidences were observed in mice lacking cyclin A2 and cyclin B1, CDK1 partner in G2/M transition and mitosis.^131,132^ On the other side, combined knockdown of all interphase CDKs as CDK2, CDK4 and CDK6, results in late embryonic lethality (E13.5). In these models the embryos show various abnormalities, mainly at the expense of liver, heart and hematopoietic tissues, reminiscing phenotypes partially observe in CDK4-CDK6 and CDK2-CDK4 coupled knockout mice.^130^ Biochemical analyses confirmed that CDK1 could interact with cyclins D1, D2, and E. Additionally, immunoprecipitation of cyclins D and E from CDK2, 4 and 6 null cells resulted in RB phosphorylation. Mouse embryonic fibroblasts (MEFs) derived from these triple knockout embryos displayed partially compromised growth in early culture stages; however, they became immortal after a few passages. These analyses suggest that CDK1 is essential for proper cell division and may compensate for the absence of other cell cycle-related kinases.^130^

##### Contribution to cell cycle progression: the mostly unexplored roles of CDK3

CDK3 (305 amino acids, located at 17q25.1) is a protein predominantly localized in the cytosol, with high expression in the respiratory tract according to the Human Protein Atlas. Along with CDK2, CDK3 is part of the CDK1-related kinase family.^49^

CDK3 interacts with and is activated by cyclins E, A, and C, with the cyclin C interaction being the most well-characterized. CDK3 increases RB phosphorylation at sites different from those phosphorylated by CDK4, likely contributing to RB hyperphosphorylation and activation similarly to CDK2.^133,134^ Studies on CDK3 dominant-negative mutants revealed a G1 block in transfected cells, a phenomenon rescued by wild-type CDK3 but not by CDK2. Dominant-negative CDK3 preferentially inhibited E2F1, E2F2, and E2F3 transcriptional activity in an RB-independent manner.^135^

CDK3 activation starts in G0 and peaks in mid-G1, indicating involvement with other partners. Ren and Rollins demonstrated a role for cyclin C and CDK3 in the G0-G1 transition. Upon mitogenic signals, cyclin C and CDK3 form complexes with RB, stimulating its phosphorylation on Ser 807/811 without promoting S phase entry, necessary for efficient G0 to G1 transition.^134,136^

Few other CDK3 substrates are identified, including Cables1, c-JUN, ATF1, ERα, and NFAT3. Cables1 is phosphorylated by both cyclin A-CDK3 and cyclin E-CDK3 complexes.^137,138^ CDK3 is also identified as an upstream activating kinase of AP-1 transactivation in response to EGF stimuli, enhancing cell transformation and proliferation via c-JUN phosphorylation.^139^ Overexpression of CDK3 and RAS G12V induces ATF1 activation, suggesting a role in the EGFR-RAS pathway. In vitro experiments suggest also that CDK3 and ERα bind in the presence and absence of estrogen.^139^ Overall, collected data suggest that CDK3 in complex with cyclin C, E or A is principally involved in the activation of cell cycle genes’ transcription to promote G1-S transition and entry into G2/M phase.

#### Transcriptional CDKs

**Fig. 4:**
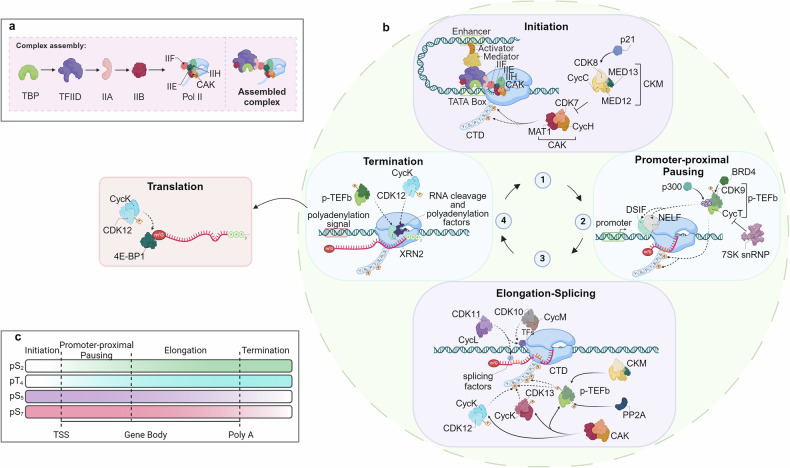
**CDK/cyclin complexes roles in the regulation of the transcriptional cycle. a** Stepwise Assembly of the Pre-Initiation Complex (PIC) and RNA Polymerase II (RNA pol II) recruitment. The assembly of PIC and the recruitment of RNA pol II is a highly coordinated process essential for initiating RNA transcription. The process begins with the TATA-binding protein (TBP) subunit of TFIID binding to the promoter region of the DNA. This binding is stabilized by the interaction with TFIIA. Next, TFIIB is recruited, which subsequently engages with the RNA pol II-TFIIF complex. Following this, TFIIE associates with RNA pol II, facilitating the binding of the TFIIH complex. The TFIIH complex includes the cyclin-dependent kinase-activating kinase (CAK) complex, which is composed of CDK7, MAT1, and cyclin H. This sequential assembly of the PIC is critical for DNA melting and the phosphorylation of the C-terminal domain (CTD) of RNA pol II, both of which are crucial steps for initiating RNA transcription. **b** The Role of Transcriptional CDKs in the different stages of transcription: **1**. PIC Assembly and Initiation: the phosphorylation of RNA Pol II CTD at Ser5 and Ser7 by CDK7 promotes initiation, promoter clearance, and co-transcriptional 5’-end capping. The CDK8 kinase module (CKM, composed of CDK8, cyclin C, Med12, and Med13) associates with the core Mediator complex, to associate activators to RNA Pol II. When not associated with RNA Pol II, CKM can negatively modulate CDK7 activity by phosphorylating cyclin H, inhibiting transcription initiation. Additionally, CDK8 function can be positively regulated by p21. **2**. Promoter-Proximal Pausing and Pause Release: this process involves the exchange of TFIIE for the elongation factor DSIF, which recruits NELF to establish a pause 50-100 bp downstream of the Transcription Start Site (TSS). CDK9/cyclin T, also known as positive Transcription Elongation Factor b (P-TEFb), phosphorylates components of the paused complex to relieve pausing. The interaction of CDK9 with BRD4 is enhanced by acetylation of cyclin T1 by p300 and phosphorylation of CDK9, facilitating the release of P-TEFb from the inhibitory factor 7SK snRNP. **3**. Elongation and Splicing: Pol II CTD Ser2 is phosphorylated by CDK9 and/or CDK12/13 to promote productive elongation and splicing events. CAK mediates CDK9 and CDK12/13 activation through T-loop phosphorylation. Additionally, CKM recruits P-TEFb during elongation, and its dephosphorylation by PP2A further enhances elongation. CDK11/CycL and CDK10/CycM can also phosphorylate transcription and splicing factors to promote splicing. **4**. Termination and translation: RNA cleavage and polyadenylation factors are phosphorylated by CDK9 and/or CDK12/13, facilitating cleavage, polyadenylation of the pre-mRNA, and Xrn2-dependent termination. CDK12/CycK phosphorylates 4E-BP1, promoting translation of specific genes in the cytoplasm. **c**. Regulation of Pol II CTD Phosphorylation During Transcription. The phosphorylation state of the RNA pol II CTD is dynamically regulated throughout the transcription cycle. As transcription progresses from initiation at the TSS, through elongation in the gene body to termination at the polyadenylation site (Poly A), distinct phosphorylation marks are added or removed to modulate specific transcriptional functions. This regulation is crucial for coordinating the various stages of transcription, including initiation, elongation, RNA processing, and termination. See the text for detailed references. (Created with BioRender.com and Adapted from “Eukaryotic Gene Regulation - Transcriptional Initiation”, by BioRender.com (2024). Retrieved from https://app.biorender.com/biorender-templates)

CDKs have essential roles not only in the cell regulation of cell cycle division, but also in the RNA-polymerase II (Pol II)-dependent transcription.^8^ A central hub for Pol II regulation is the carboxy-terminal domain (CTD) of the largest subunit of Pol II, Rpb1, which includes heptad repeats (52 in humans) of the consensus sequence Y_1_S_2_P_3_T_4_S_5_P_6_S_7_. Multiple positions within the heptads are phosphorylated by CDKs and other kinases, modulating the binding of factors required for transcript elongation, RNA processing and chromatin modification,^140^ leading to the so-called “transcription cycle”.^141^ A useful way to visualize the role of specific Pol II regulatory kinases is to schematize the different stages of transcription: 1) pre-initiation complex (PIC) assembly and initiation; 2) promoter-proximal pausing and pause release; 3) elongation and splicing, and 4) termination (Fig. 4). Within this scenario, CDK7, CDK8/19, CDK9 and CDK12/13 are the best characterized actors in controlling the distinct stages of Pol II transcription. In contrast to cell-cycle CDKs, transcriptional CDKs (tCDKs) usually present a single cyclin partner and are recruited to the transcriptional machinery as a part of larger protein complexes (Fig. 4).

##### From transcriptional regulation to CDKs activation: the multifaced role of CDK7

CDK7 (also known as CDKN7 located at 5q13.2) is a 346 amino acid protein and was among the first CDKs to be implicated in Pol II transcription. CDK7 directly controls initiation and indirectly affects promoter-proximal pausing,^142^ elongation and splicing.^143^ Differently from canonical CDKs, some non-canonical CDKs require additional subunits for their full activation. One of them is CDK7, which is integrated into large transcriptional CDK-activating kinase complex (CAK). The cyclin partner of CDK7 is cyclin H, which requires an additional subunit, the RING finger protein MAT1, to form the active ternary CAK complex, as firstly described in 1995.^144^ CAK phosphorylates cell cycle-related CDKs (such as CDK1, CDK2, CDK4 and CDK6) and transcriptional CDKs (CDK9, CDK12 and CDK13) in the T-loop, participating in their activation.^145^

Having a dual functionality, CAK is involved in the early steps of RNA Pol II transcription, assembling into the pre-initiation complex (PIC) at Pol II promoters, as a component of the general transcription factor TFIIH (Fig. 4a). In particular, CDK7, in complex with cyclin H and MAT1, phosphorylates Ser5 and Ser7 of the Pol II C-terminal domain (CTD),^145^ promoting co-transcriptional 5’-end capping of the nascent transcript.^140^ This CTD mark is related predominantly on Pol II transcribing the promoter-proximal and upstream regions of genes, reinforcing the notion that CDK7 fundamental functions are executed early in the transcription cycle^142^(Fig. 4b, point 1).

However, CDK7-dependent events occur throughout the Pol II cycle. Chemical genetics approaches revealed a role for CDK7 in establishing the promoter-proximal pause of Pol II elongation. This pause regulates genes expression by two mechanisms: 1) by maintaining the genes in an inactive state, responsive for rapid activation concomitantly to appropriate signals and, 2) by reinforcing the coupling between transcript elongation and co-transcriptional processes.^146^ Paused Pol II is stabilized by the exchange of an initiation factor, TFIIE, with the two factors DSIF (DRB-sensitivity-inducing factor) and NELF (negative elongation factor) (for a more accurate description refers to^147^), an observation confirmed by chemical genetics with the identification of a small inhibitor molecule of CDK7 (THZ1).^148^ Paused polymerase is released by the positive transcription elongation factor b (P-TEFb), which contains the kinase CDK9 and its predominant cyclin subunit cyclin T1. CDK7 might also trigger Pol II for subsequent phosphorylation by CDK9, CDK12 and CDK13, which all prefer CTD-derived peptides previously phosphorylated at the Ser7 position of the heptad repeat.^145,147,149^

The effects of CDK7 are not restricted to the early stages of the Pol II transcription cycle: alterations in chromatin modification patterns, impairment in 3’-end formation and delayed termination have all been observed after CDK7 inhibition. CDK7 activity is crucial for histone modifications, such as H2B monoubiquitination^147^ and H3K4 and H3K36 trimethylation, facilitating recruitment of histone methyltransferases SETD1A/B and SETD2 and affecting transcription and elongation (Fig. 4b, point 3).^147,150^

Interestingly, trimethylated H3K4 modifications play important roles in pre-mRNA splicing, suggesting a possible involvement of CDK7 in RNA processing.^151^ Indeed, CDK7’s involvement in transcription extends to RNA processing and splicing, with substrates including different components of the splicing machinery (e.g. SF3B1 and U2AF2).^152^ Beside CTD phosphorylation, CDK7 phosphorylates several transcription factors including p53, E2F1, Ets1 and multiple nuclear hormone receptors (retinoic acid receptor alpha and gamma, RAR-α and RAR-γ, androgen receptor, AR, estrogen receptor alpha, ER-α) to promote their full activation and subsequent degradation.^153^

TFIIH core and associated CAK, could also mediate the response to DNA damage and structural interactions between the CAK and TFIIH core subunits, in particular during nucleotide excision repair (NER) processes.^143^ The XPA subunit catalyzes the detachment of the CAK from the core, together with the involvement of the other NER-specific factors. The release of the CAK from the core TFIIH promotes the incision/excision of the damaged oligonucleotide and thereby the repair of the DNA. Following repair, CAK re-associates with TFIIH, enabling transcription to proceed. Accordingly, CAK inhibition improves repair efficiency, suggesting that CAK could be a negative regulator of NER.^143,154^

Considering that cell cycle CDKs have profound influence on transcription, it is reasonable to consider the reciprocal effect of transcriptional CDKs on the cell cycle. CDK7 is a good example of “dual kinase”, which is being traditionally classified as transcriptional CDK, but it was initially identified as metazoan CAK, required for the activation of both CDK1 and CDK2, by phosphorylating the T-loop region, to promote cell cycle progression.^155^ In multicellular organisms, CDK7 is fundamental for cell proliferation and development and its deficiency leads to early embryonic lethality in mice and premature aging of adult tissues with high proliferative ability, such as skin or intestinal epithelium.^156^ In human cells, CDK7 activity is essential for, mitotic entry, DNA replication and G1 phase progression through activation of CDK1, CDK2 and CDK4/6, respectively. Similarly, selective inhibition of CDK7 by pharmacological approaches, primarily inhibits E2F-driven gene expression causing G1-S cell cycle arrest.^157^

Cell cycle CDKs require to be fully active of both cyclin binding and T-loop phosphorylation, to achieve a fully active state. This classical two-step mechanism does not explain the activation of some transcriptional CDKs. The first crystal structure of human CDK7 bound to ATP was reported in 2004, revealing a typical CDK kinase fold with the catalytic pocket positioned between the N-terminal and C-terminal lobes.^158^ Yet, despite several progresses have been made regarding the structure definition of CAK across different species, the structure of cyclin H-CDK7 dimer remains still unavailable.^159,160^ The CDK7-cyclin H conformation can be positioned in between the “closed” form of CDK2-cyclin A and the “open” form of CDK9-cyclin T.^154^ Indeed, within the CAK complex, CDK7 is in an active state even though the conserved threonine residue (T170) in the T-loop remains unphosphorylated.^161^ Human CDK7 contains an additional phosphorylation residue, Serine 164 (S164), located within its T-loop. S164 is located in proximity to a positively charged pocket formed by three arginine residues, from each subunit of the CAK complex (R137, R295 and R165 from CDK7, MAT1 and cyclin H, respectively). This interaction hub facilitates the assembly of the complex, potentially stabilizing CDK7 T-loop. In line with this observation, a highly conserved tyrosine residue (Y190 in human CDK7), in the Y-loop of CDKs, plays an important role in the stabilization of the T-loop in an active conformation, establishing extensive contacts with MAT1 subunit and releasing the hindrance in the catalytic cleft.^159,160^ Considering large complexes, these precise activation processes favor the accessibility of the ATP binding pocket and substrate binding site, allowing the kinase to proficiently phosphorylate its substrates. Finally, the CAK complex is not only activated, but can also be inactivated through phosphorylation of S5 and S304 in Cyclin H, by the kinase module CDK8-cyclin C of the Mediator complex.^162^

##### Transcriptional co-activators or co-repressors: the role of CDK8 (and its paralog CDK19)

CDK8 (gene located at 13q12.13) is 464 amino acid protein firstly identified as human protein kinase (K35) in 1995.^37^ It associates with cyclin C, MED12 and MED13 to form a 600-kDa complex, also known as CDK8 kinase module (CKM) (Fig. 4B, point 1). CDK8 can associate with the Mediator complex, which is a multi-subunit (25-30 proteins) transcriptional coactivator complex that transmits regulatory signals from transcription factors to the Pol II transcription machinery.^37,163^ Additionally, vertebrate paralogs of MED12, MED13 and CDK8 have been identified and defined as MED12-like (MED12L), MED13-like (MED13L) and CDK8-like (CDK19), with 59%, 53% and 83% of sequence homology, respectively. These paralogs can form part of the CKM, but are mutually exclusive of each other. As a consequence 8 different cyclin C-CDK modules could be assembled, suggesting that each complex has potentially distinct functions.^164^

Multiple studies have indicated that CDK8 can function as either a repressor or an activator of a subset of genes, depending on cell type or transcription-cycle stage or in response to different stimuli.^8,140^ It could be hypothesized that this cell-type specificity is due to chromatin structure and histone modifications, which give rise to cell-type specific enhancers and transcription factors (TFs) binding patterns. The indirect predominant function of CDK8 and CDK19, in the control of Pol II transcription process, is related to the phosphorylation of several transcription factors (Fig. 4b, point 1). To support this notion, a large-scale proteomics study revealed that many proteins phosphorylated by CDK8 and CDK19 are DNA-binding, general TFs (e.g. the TFIID subunit TAF10, the Super Elongation Complex SEC, NELFA) or chromatin remodelers and modifiers (SETD1A, CHD4, KDM3A).^165^ CDK8/CDK19 knockdown has minimal impact on basal gene expression and is well tolerated in cell under normal growth conditions. However, in response to stress or developmental cues, activation/repression of several gene sets largely depend on CDK8 or CDK19 expression, likely for the need to establish new transcriptional programs mediated by TF phosphorylation.^164^

CDK8 can negatively regulate transcription through at least two mechanisms. First, CDK8 can phosphorylate the free Pol II CTD at Ser2 and Ser5 in vitro, to prevent its recruitment to the PIC. CDK8 can impede, by competition with Pol II, Pol II-Mediator interaction, necessary for the formation of the PIC.^166,167^ Then, cyclin C-CDK8 complex phosphorylates cyclin H-CDK7, repressing its ability to promote co-transcriptional 5’-end capping of the nascent transcript (Fig. 4B, point 1).^162^

Subsequent investigations have unveiled a positive regulatory role for CDK8 in transcription. In particular CDK8-Mediator complex is considered a positive regulator of transcriptional elongation, promoting the recruitment of the bromodomain protein BRD4 and(P-TEFb, to trigger transcription elongation on hypoxia-inducible genes^168^ and serum-responsive immediate early genes (Fig. 4b, point 3).^169^

Similar to CDK7, also CDK8 could be ascribed as a dual kinase, participating both in transcriptional processes and cell cycle regulation. One of CDK8 targets is the CDK inhibitor p21.^107^ Inhibition of CDK8 induces a decrease in p21 mRNA accumulation, whereas CDK8 overexpression stimulates p21 transcription, suggesting that CDK8 promotes cell cycle arrest stimulating the p53-p21 pathway. Interestingly, the p21 protein directly interacts with CDK8 and stimulates its kinase activity, creating a potential positive feedback loop.^169,170^

Phosphorylation of cyclin H-CDK7 is another mechanism through which CDK8 indirectly controls the cell cycle since CDK7 (the CAK kinase) phosphorylates and activates CDK1/2/4/6 in the T-loop to regulate cell cycle progression. Although there are no direct evidences for a direct CDK8 influence on the mammalian G2/M transition, it has to be noted that the fission yeast CDK8 homolog Fkh2, controls a cluster of genes expressed at the onset of the mitosis.^145^

In contrast to many other cyclins affecting cell cycle progression, the levels of cyclin C do not oscillate during the cell cycle and cyclin C-CDK8 is a relatively stable complex. In addition, differently from cell cycle CDKs, CDK8 is not regulated by T-loop phosphorylation, since its T-loop lacks a Ser/Thr acceptor site. This suggest that CDK8 presents different activation and regulation mechanisms.^171^

Together with cyclin C, the kinase activity of CDK8 and CDK19 is regulated in particular by MED12 interaction with both T- and Y-loops contributing to their stabilization. Since cyclin C ablation prevents the association of CDK8 with MED12 and MED1 it is possible to speculate that a regulatory loop govern CDK8 activation: the interactions with Mediator subunits not only stimulates gene transcription, but also initiates a CDK8-dependent process that may stabilize or destabilize the activator itself.^164^ This regulatory loop is likely similar although not identical, for CDK19.

CDK8 and CDK19 present high sequence conservation in the cyclin C binding domains, but diverge significantly in C-terminal tail, indicating possible different interactions and functions.^172^ The overlap and extensive redundancy between CDK8 and CDK19 complicate the comprehension of the single effect of their CDK activity. However, emerging evidences suggest that CDK8/19 are not completely functionally redundant, in the control of the transcriptional program.^168^ Genetic studies in flies, mice and cultured cells, have revealed unique roles of CDK8 in embryonic development, differentiation and expression of glycolysis-pathway genes,^140^ whereas evidences for a unique physiological role of CDK19 are still in their infancy. For example, loss of CDK8, cyclin C, or Mediator kinase module subunits (as MED12), but not of CDK19, is lethal in mice during embryo development (E2.5-3), likely due to transcriptional de-regulation.^145,164^ Quite surprisingly, even if single knockout of CDK8 and cyclin C results in embryonic lethality before implantation (E2.5-3), conditional deletion of CDK8 in adult mice is well tolerated.^173^ On the other side, preliminary studies of CDK19 KO mice illustrated no significant behavioral or phenotypical abnormalities and CDK19 seems to be dispensable for embryonic development and adult tissue homeostasis.^174^ Of course, further studies are necessary to clarify the specific physiological cellular functions of CDK19.

##### Transcriptional elongation and termination: the role of CDK9

CDK9 (located at 9q34.11), a 372 amino acid protein, first described as PITALRE based on its conserved cyclin-binding peptide motif, is the most extensively studied transcriptional CDK. It was firstly cloned in 1994 by Graña et al., using approaches aimed to identify Ser/Thr protein kinases possibly involved in cell cycle regulation.^38^ The major cyclin partner of CDK9 is cyclin T1, but it can also associate with cyclin T2a and T2b, which have a high degree of identity (81%) in their cyclin box region. Additionally, CDK9 can also form complexes with cyclin K. The cyclin T-CDK9 complex is canonically referred as positive transcription elongation factor b (P-TEFb), a potent general transcription factor, which regulates the elongation phase of transcription by RNA polymerase II.^175^ Two CDK9 isoforms, CDK9_42_ and CDK9_55_, are produced from different transcription start sites in the first exon of the CDK9 gene. Their functional significance have yet to be elucidated.^176^ P-TEFb activity is necessary to overcome promoter-proximal pause and continue elongation process during transcription. In particular, P-TEFb phosphorylates one of the four NELF (negative elongation factor) subunits and the DSIF (DRB-sensitivity inducing factor) factors, thus releasing Pol II from pausing (Fig. 4, point 2).^177^ At the same time, P-TEFb phosphorylates Ser 2 (but also Ser 5 and 7 and Thr 4) on the Pol II CTD, efficiently coupling transcription elongation and pre-mRNA processing.^178^ Beyond pause release, some studies attribute to CDK9 a possible role in transcriptional initiation by regulating the frequency of any new round of transcriptional initiation (defined as “pause-initiation” limit) and suggesting a way by which cells could maintain the appropriate quantity of RNA transcribed from specific genes.^179^

Emerging evidences suggest a role for CDK9 in transcription termination. Similar to promoter-proximal pausing, an additional major elongation checkpoint, dependent on P-TEFb, has been identified near the terminal poly(A) sites (Fig. 4B, point 4).^180^ A chemical genetics approach used to identify additional putative substrates of CDK9, demonstrated that they are enriched for factors involved in transcription and RNA processing, including the 5’-3’ exoribonuclease XRN2. Down-modulation of CDK9 leads to decreased chromatin localization of XRN2 and increased read-through transcription, clearly involving CDK9 in efficient transcription termination (Fig. 4B, point 4).^181^ Finally, CDK9 phosphorylates and inhibits protein phosphatase 1 (PP1) activity on DSIF and Pol II, leading to transcription termination.^182^

Dissimilar to cell cycle CDKs, the expression of CDK9 and its cyclin partners, and the kinase activity of the complex, does not change in a cell cycle-dependent manner. In this light, the possible cell cycle functions of CDK9 remain elusive. However, depletion of CDK9 in cancer cell lines, lead to cell cycle delay with an accumulation in G1 and corresponding decrease in S phase.^145^ Moreover, knockdown of CDK9 in Drosophila cells induces cell cycle arrest in G1.^183^ Additionally, CDK9 phosphorylates the RB protein in vitro and in vivo, which can influence Pol II dependent gene transcription.^184^ Interestingly, independently from canonical interaction with cyclin T, CDK9-cyclin K activity seems to be necessary for cell cycle recovery after replication stress. Loss of CDK9 activity causes an increase in spontaneous levels of DNA damage signaling in replicating cells couple to a decreased ability to recover from a transient replication arrest.^185^

These observations shed light on possible roles of P-TEFb complex also in DNA repair. In particular the function of cyclin K-CDK9 complex emerged with the identification of cyclin K as a transcription target for p53 in the response to DNA damage.^186^ Moreover, CDK9-cyclin K seems to play a direct role in the repair of damaged DNA by interacting with member of the ATM and ATR pathway.^185^

The activity of P-TEFb is highly regulated by numerous transcriptional, translational and posttranslational mechanisms. More than half of cellular P-TEFb is reversibly maintained under stringent negative regulation by the large inhibitory ribonucleoprotein complex 7SK snRNP. In this complex, P-TEFb is mainly sequestered by HEXIM1/HEXIM2 proteins, and represents a major reservoir of transcriptionally inactive P-TEFb as a source to facilitate Pol II escape.^175^ The exact molecular mechanism for the release of P-TEFb from 7SK snRNP, in response to cellular signals or stress conditions, remains to be elucidated. Of note, a small fraction of active P-TEFb can be found in the Super Elongation Complex (SEC), a multicomponent transcription activator. Alongside BRD4 and SEC, other transcription factors, such as NF-κB, might directly or indirectly bring P-TEFb to target genes.^177^

The activity of P-TEFb is further controlled by extensively post-translational modifications toward CDK9 and cyclin T1. CDK9 follows the classical two-step activation mechanism, initially interacting with cyclin T and then undergoing Thr186 phosphorylation in the T-loop, induced by autophosphorylation and by CDK7/CAK complex activity.^147,177,187^ After P-TEFb release, CDK9 is also phosphorylated on a second highly conserved T-loop residue, Ser175, which promotes the binding with BRD4. Conversely, CDK9 activity is reduced by Thr29 phosphorylation, which mainly occurs in the pre-initiation complex and it is necessary for limiting CDK9 function during transcription initiation. Moving to elongation, PP2A dephosphorylates pThr29 favoring the positive elongation function of CDK9.^177^ Beside phosphorylation, acetylation of four lysine residues of cyclin T1 by histone acetyl transferase p300 (Lys380, Lys386, Lys390 and Lys404), determines the detachment of P-TEFb from 7SK snRNP and is found only in the active P-TEFb complex.^188^

##### Less studied transcriptional CDKs: the roles of CDK10

Cyclin-dependent kinase 10 (CDK10 located at 16q24.3), previously known as PISSLRE, is encoded by 14 exons and contains regulatory residues, such as Thr196, typical of the CDK protein kinase family.^189,190^

Cyclin M (also known as cyclin Q) is the primary binding partner of CDK10. The binding between cyclin M and CDK10 is necessary for CDK10 activation.^191^ Moreover, cyclin M expression prevents CDK10 proteasome-mediated degradation, enhancing its stability.^191^ Cyclin M gene (*FAM58A*, located at Xq28 locus) loss-of-function mutations have been identified in STAR-syndrome, an X-linked rare genetic disorder characterized by syndactyly (fusion of two or more digits together), telecanthus (increased distance between the inner corners of the eyelids with a normal the inter-pupillary distance) and anogenital and renal malformations.^192^ STAR-associated cyclin M mutations compromise cyclin M-CDK10 complex formation and CDK10 kinase activity, leading to the pathological onset of the syndrome.^191^

Different CDK10 transcripts are generated by alternative splicing processing. Two main isoforms have been described: the full-length sequence CDK10-P1 and CDK10-P2, harboring a 29 amino acid truncation at the N-terminus and a modified C-terminus.^189^ Notably, CDK10-P2 fails to form a functional complex with cyclin M.^191^

CDK10 stability and regulation is likely driven by phosphorylation and dephosphorylation events, which are crucial for efficient activation and inactivation processes. Indeed, CDK10 kinase activity is achieved by phosphorylation at the conserved Thr196 in the T-loop and its phosphorylation at Thr133 is required for Pin1, leading to CDK10 ubiquitin-proteasomal degradation.^193,194^

CDK10 is principally involved in transcription elongation and splicing through the phosphorylation of transcription factors (Fig. 4B point 3). Among these, the most extensively studied is ETS2, a transcription factor involved in cellular proliferation and development.^195^ ETS2 phosphorylation by CDK10 reduces its transactivation and enhances its proteasomal degradation.^196^ Furthermore, mutations in cyclin M associated with STAR syndrome have been correlated with elevated ETS2 protein levels.^191^ Moreover, CDK10 could directly associates with RNA polymerase II CTD, c-MYC, and RB, thereby controlling transcription and cell proliferation.^193^

Genetic inactivation of CDK10 in mouse models shows severe post-natal effects, including growth retardation and skeletal abnormalities that resemble human growth deficiencies related to CDK10 germline mutations.^197^ However, CDK10 expression has also been identified in non-proliferative tissues, suggesting that its roles can go beyond cell cycle progression and growth regulation.^189^

##### Transcription elongation and splicing regulation: the role of CDK11

CDK11, formerly identified as PITSLRE, is encoded by two distinct human genes, *CDK11A* and *CDK11B*, located at 1p36.3 locus and encompassing 20 exons each.^198–200^ CDK11 is highly related to CDK1 and CDK2, especially in the catalytic kinase domain.^8^ The two primary isoforms, CDK11^p110^ and CDK11^p58^, are distinguished.^201^ CDK11^p110^ contains a large C-terminal kinase domain and an N-terminal domain that harbors nuclear localization signals (NLS), caspase cleavage sites, and an arginine-glutamate (RE) rich domain, which is crucial for protein-protein interactions and localization to nuclear speckles, the subnuclear structures enriched in pre-messenger RNA splicing factors.^198^ The mitosis-specific CDK11^p58^ isoform retains the C-terminal kinase domain and the caspase cleavage sites while lacks the CDK11^p110^ N-terminal extension. This highlights the possibility of different substrate specificity and functional roles deriving from these structural differences. CDK11^p110^ and CDK11^p58^ expression levels are ubiquitously detected in all human tissues, with particularly high levels observed in the heart, brain, placenta, skeletal muscle, kidney, and pancreas.^198^

Other CDK11 isoforms, namely CDK11^p46^ and CDK11^p60^, are produced through caspase cleavage. In addition, it has been shown that serine phosphorylation of the CDK11^p110^ protein, mediated by the apoptotic Fas signaling activation, is fundamental for caspase cleavage. CDK11^p46^, generated during TNF- and Fas-mediated apoptosis processes, retains the C-terminal kinase domain necessary for the phosphorylation of proteins involved in apoptotic pathways.^202^

The primary binding partners of CDK11 isoforms are L-type cyclins, encoded by two genes, (*Ania-6b* cyclin L1 and *Ania-6a* cyclin L2) that generate multiple transcripts, translated into six distinct proteinscyclins (L1α, L1β, L1γ, and cyclins L2α, L2βA/B). All three primary isoforms of CDK11 interact with both α and β isoforms, while L1γ does not form complexes with CDK11.^203^

CDK11^p110^ is involved in both transcription and splicing (Fig. 4b point 3), suggesting a potential interconnection between these two processes. CDK11^p110^, L1α, and L2α colocalize in nuclear speckles where they bind both splicing factors, (e.g. SF2/ASF and 9G8), and RNA polymerase II.^203^

Further evidences indicate that CDK11 interacts and phosphorylates Ser 2 of RNA Polymerase II, thus modulating proper chromosome segregation during mitosis^204^ and replication‐dependent histone (RDH) mRNAs transcription elongation and 3′-end processing during the S phase.^205^ However, CDK11-dependent phosphorylation of RNA pol II CTD in vitro is less efficient compared to the one of CDK12 or CDK9, the two transcriptional CDKs mainly involved in Pol II CTD phosphorylation at Ser2.^205^

CDK11^p110^ is also involved in the regulation and promotion of pre-mRNA splicing events, likely through the interaction with the RNPS1 and 9G8 factors. RNPS1 (RNA Binding Protein with Serine Rich Domain 1) is a component of the exon-exon junction complex (EJC) involved in mRNA splicing and quality control, while 9G8 is implicated in pre-mRNA splicing and RNA nuclear export. Interestingly, CDK11^p110^- dependent phosphorylation of 9G8 enhances its splicing activity in vitro.^206^ Additionally, CDK11 involvement in splicing processes is supported by the observation that overexpression of CDK11p^110^ or cyclin L1/2 increases intron splicing events in vitro. On the contrary, the expression of CDK11^p58^, CDK11^p46^ or the absence of functional CDK11^p110^, resulted in decreased splicing efficiency.^207^ Additionally, CDK11^p110^ or cyclin Lα depletion from the nuclear extract altered in vitro splicing of a β-globin pre-mRNA substrate, reinforcing the role of this complex in splicing regulation.^206^

Checkpoint kinase 2 (CHK2), a crucial kinase involved in the response to several genotoxic cellular stress, has been identified as an upstream activator of CDK11 in pre-mRNA splicing processes and phosphorylates CDK11^p110^ in a DNA damage-independent manner, thereby promoting CDK11 homodimerization and fostering alternative splicing.^208^ Finally, a direct association between CDK11 depletion and intron retention enrichment has been established by identifying the spliceosome component SF3B1 as a binding partner and substrate of CDK11. Phosphorylation of SF3B1 by CDK11 enhances its binding to U5 and U6 snRNAs during the formation of the active B spliceosomal complex.^209^ As a consequence selective chemical inhibition of CDK11 resulted not only in the accumulation of non-functional B complexes on pre-mRNAs, resulting in intron retention, but also in altered occupancy and phosphorylation of RNA polymerase II at Ser2.^209^ These observations underscore the interplay between transcriptional and RNA processing events, wherein increasing evidence suggests CDK11 involvement.

##### Regulation of transcription, splicing and genome stability: role of CDK12 and its paralog CDK13

CDK12 (also known as CRK7/CrkRS located at 17q12), encoded by the *CDK12* gene consisting of 14 exons, is a serine/threonine kinase composed of 1490 amino acids.^210^ Its paralogue gene CDK13, (also known as CDC2L5 or CHED, located on 7p14), encodes for a 1512 amino acid protein.^211^ CDK12 and CDK13 are fundamental kinases to transcriptional regulation and genome stability maintenance.^212,213^ Cyclin K plays a crucial role in modulating CDK12 and CDK13 kinase activity and substrate recognition, by forming distinct complexes with either CDK12 or CDK13.^212,214^ In vivo genetic inactivation of cyclin K, CDK12, or CDK13 resulted in early embryonic lethality, emphasizing the essential roles of these complexes in gene expression regulation and cellular survival.^215,216^

CDK12 and CDK13 exhibit significant sequence homology (43%) and possess a highly similar central kinase domain ( > 93% identity), which features a conserved PITAIRE motif, crucial for maintaining kinase domain structure and catalytic function.^214^ It also contains N- and C-lobes, forming the ATP-binding pocket necessary for substrate recognition and phosphorylation. Within the conserved domain, certain amino acids are key for kinase activity regulation by post-transcriptional modifications.^217^ For instance, mutation of Thr893, located in the activation T-loop, and of Arg882 impair CDK12 kinase activity and are implicated in ovarian cancer pathogenesis. Phosphorylation at Thr893 by CAK (CDK7 complex), enhance CDK12 kinase activity beyond the basal level achieved by the formation of cyclin K-CDK12 complex.^217^

CDK12 exhibits high expression in the G1 phase, decreases during the S phase, and then rises again in the G2/M phase, suggesting an important role also in cell cycle progression. CDK12 activity is essential for an optimal G1/S progression and its depletion results in G2/M phase accumulation.^218^ However, understanding the regulation of critical phosphorylation sites on CDK12 and their consistency across cell cycle stages requires further investigation.

In contrast to other proline-directed kinases, such as CDK1, CDK2, or CDK9, CDK12 and CDK13 exhibit unique substrate specificity. Their preferential targets are sequences surrounded by negatively charged aspartate and glutamate residues, which are phosphorylated independently of a proximal C-terminal proline or a + 2 arginine/lysine residue.^217^

CDK12 and CDK13 play pivotal roles in transcriptional elongation, manifesting both shared and distinct molecular functions (Fig. 4b, point 3 and 4). One of their main common targets is RNA pol II CTD that both CDK12 and CDK13 could phosphorylate at Ser2 and Ser5.^213,218,219^ CDK12/13 modulate the transcriptional elongation of RNA Pol II through dynamic phosphorylation of its Ser2 CTD only in a small subset of genes, while leaving the global transcription rate unaltered.^215^ Dual chemical inhibition of CDK12 and CDK13 alters RNA Pol II Ser2 phosphorylation significantly, affecting its processivity and elongation rate, leading to alterations in the 3’ polyadenylation site (PAS) selection of long genes with intronic alternative polyadenylation sites.^220,221^ The usage of alternative PAS results in shorter transcripts, affecting particularly DNA Damage Response (DDR) genes and resulting in deficiency in the Homologous Recombination (HR) DNA repair pathway, tightly linking RNA transcription regulation with DDR.^220,221^ This activity is more associated to CDK12 than CDK13 expression and CDK13 depletion has a minimal impact on the expression of DDR-related genes while mostly affecting the expression of genes belonging to cell growth and metabolic signaling pathways.^220^

Although the precise mechanisms governing CDK12 and CDK13 regulation and activation via cellular pathways or epigenetic modifications are not yet fully understood, such regulatory mechanisms may involve cellular localization or intra-molecular interactions, potentially mediated by N-terminal arginine/serine (RS) elements.^214^ CDK12 has twenty and CDK13 seventeen RS motifs in their N-terminus and localize to nuclear speckles, sub-nuclear, membrane-less organelles where splicing factors accumulate.^214^ RS domains, commonly found in splicing factors, regulate splicing factors’ function by facilitating protein-protein interactions and acting as docking sites and their phosphorylation can modify the RNA-binding specificity, thereby influencing alternative splicing mechanisms.^222^

CDK12 links transcriptional elongation to RNA processing in several ways, including post-transcriptional functions in RNA splicing, transcription termination, and RNA export (Fig. 4b). Mass spectrometry analysis of the CDK12 interactome has revealed associations with various RNA-binding proteins, including those in the exon junction complex (EJC), SR splicing factors (SRSFs), and constituents of the spliceosome involved in pre-mRNA splicing processes.^223^ The N-terminal SR domains of CDK12 is essential for interaction with SRSFs, facilitating the recruitment of RNA cleavage and polyadenylation factors at the 3′ end, required for processing, for instance, of MYC transcript.^224^

CDK12 plays a critical role in the maturation of long mRNAs containing cryptic intronic polyadenylation sites (PAS), acting to suppress intron premature polyadenylation (IPA), thus preventing the formation of truncated mRNA isoforms.^220,221^ This function is likely achieved through dual mechanisms: by enhancing the processivity of RNA Polymerase II via Ser2 phosphorylation and by directly phosphorylating factors involved in 3’-end processing. CDK12 implication in IPA suppression is supported by the analyses of CDK12-deficient cells and of CDK12 chemical inhibition. In both cases CDK12 inhibition results in high of levels prematurely cleaved and polyadenylated mRNAs of DDR genes.^220^ The impact of CDK12 on alternative splicing has also been shown by an in-depth bioinformatic examination of the CDK12 transcriptome, which particularly linked CDK12 to both gene-specific and cell-type-specific Alternative Last Exon (ALE) splicing.^225^

Beside these prevalent roles of CDK12 and CDK13 in RNA transcription and splicing, recent observations suggest their possible involvement also in the control of mRNA translation and nuclear export (Fig. 4b, point 4). Genome-wide analysis revealed that CDK12 is essential for the translation of approximately 200 select genes, predominantly involved in mitosis.^226^ CDK12 can directly phosphorylate Ser65 and Thr70 residues of the eukaryotic translation initiation factor 4E-binding protein 1 (4E-BP1), thereby regulating the translation of a specific subset of mitotic genes.^226^ Consequently, cells deficient in CDK12 display multiple mitotic anomalies, such as chromosome misalignments, spindle pole detachments, and a significant increase in metaphase-arrested cells, highlighting a critical role for CDK12 in maintaining mitotic fidelity.^226^

Recent observations highlighted a CDK13 specific role in RNA surveillance to specifically control prematurely terminated RNAs (ptRNA) derived from intronic polyadenylation sites. Altered RNA surveillance in CDK13^mut^ melanoma cells results in the stabilization and translation of aberrant protein-coding RNAs, thereby highlighting CDK13 distinct roles in RNA metabolism compared to CDK12.^227^

In summary, CDK12 and CDK13 are implicated in several steps of RNA metabolism, which include RNA transcription, processing, maturation, surveillance and translation. Even if both CDK12 and 13 are still categorize as understudied kinase and included in the DKK database, it is clear that their multifaceted activities underscore their essential roles in both gene expression, translation and genome stability regulation.

##### The understudied transcriptional CDK: which role(s) for CDK20?

CDK20, (located at 9q22.1 and also known as CCRK, PNQALRE, or p42) is a 42 kDa protein exhibiting a 43% sequence homology with CDK7.^228^ Its function entails promoting cell growth during normal tissue development in both cell cycle-dependent and cycle-independent manners.^229^ While predominantly localized in nuclear and perinuclear regions, CDK20 has also been detected in the cytoplasm, with the mechanisms governing this nuclear-cytoplasmic shuttling still to be elucidated.^229^ The cytoplasmic fraction of CDK20 has been linked to the regulation of ciliogenesis, modulating the morphology and length of primary cilia by regulating the Hedgehog pathway, necessary for neuron patterning and eye development.^230^ Mice lacking CDK20 expression present a wide spectrum of developmental abnormalities, including exencephaly (rare malformation of the neural tube with a large amount of protruding brain tissue and absence of skull), preaxial polydactyly (thumb duplication), skeletal malformations, and microphthalmia (one (unilateral) or both (bilateral) eyes are abnormally small with anatomic malformations).^230^

Regarding its nuclear functions, similar to CDK7, CDK20 has been acknowledged as a CAK, capable of phosphorylating Thr160 of CDK2, when in complex with cyclin H.^228^ Accordingly, knockdown of CDK20 induces cell cycle arrest in the G1 phase by diminishing levels of phosphorylated CDK2 and hindering its kinase activity.^228^ Opposing results have been reported in other models where CDK20 knockdown did not induced cell cycle arrest nor any appreciable change in CDK2 phosphorylation.^231^ These differences may imply that CDK7 is the main CAK in controlling cell cycle progression.

The human *CCRK* gene, encoding for CDK20, is in general widely expressed throughout human tissues, with a prominent presence in the brain, lung, kidney, and gastrointestinal tract.^232^ In human hepatocellular carcinoma (HCC) cells where *CCRK* transcription and protein expression are increased by direct binding of the androgen receptor (AR) to the androgen-responsive element within the *CCRK* promoter. Hyperactivated CDK20 phosphorylates and activates Enhancer of zeste homolog 2 (EZH2), a component of the Polycomb repressive complex 2 (PRC2) responsible for catalyzing repressive histone H3 lysine 27 trimethylation (H3K27me3), thereby activating transcription.^233^ We might conclude that, with the data available, although CDK20 might be functionally categorized as a transcriptional CDK for its similarity with CDK7, the mechanisms driving its expression and activation and role(s) in epigenetic or transcriptional processes, have yet to be fully understood.

#### Atypical CDKs

A simple literature search using Pubmed or analog research engines, immediately clarify that while cell cycle-related CDKs and some of transcriptional CDKs have been extensively studied, most of the others, namely “atypical CDKs”, are quite unexplored. Among them CDK5 (PSSALRE) is the most studied and represents the ancestor of the two subfamilies named, based on the sequence of their amino acid motif found in prototypic CDK1 kinase, as PFTAIREs (CDK14 and CDK15) and PCTAIREs (CDK16, CDK17 and CDK18) (Fig. 2).

##### CDK5: the neuron specific CDK

**Fig. 5:**
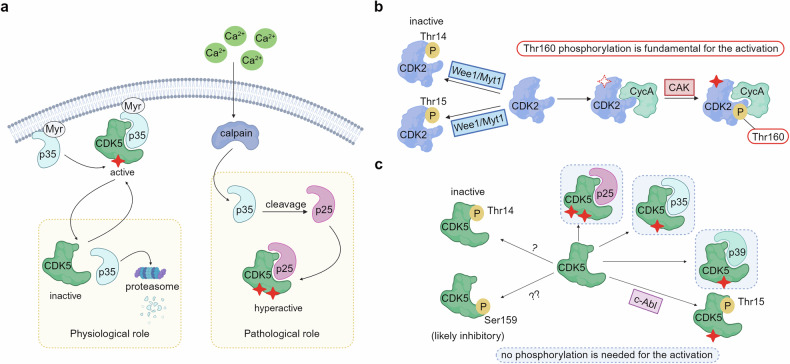
**Dynamics of CDK5 activation in comparison with cell-cycle CDKs. a** In a physiological context, p35 is membrane-bound due to its myristoylation signal, which recruits and activates CDK5. The binding of CDK5 to this co-factor is essential for its kinase activity. In various pathologies, the proteolytic cleavage of p35 into p25 by calpain is dysregulated, increasing the stability of p25 and leading to CDK5 hyperactivation. **b**, **c** The regulation of CDK5 (**c**), an atypical CDK, differs significantly from the classic regulation of cell-cycle CDKs (**b**), despite its high similarity to CDK1 and CDK2. For cell-cycle CDKs, the binding of cyclins confers low activity, which requires CAK phosphorylation for full activation. Unlikely, the activation of CDK5 does not require cyclins or phosphorylation upon co-factor binding. The inhibitory phosphorylation on Thr14 inside the binding cleft, which in CDK2 is mediated by WEE1/MYT1, has been poorly investigated in CDK5. Conversely, phosphorylation on Thr15, which inactivates CDK2 (also mediated by WEE1/MYT1 for CDK2 and not well studied for CDK5), activates CDK5. Additionally, while Thr160 phosphorylation is crucial for CDK2 activation, the corresponding phosphorylation site in CDK5 (Ser159) is likely inhibitory. CDKs full and partial activation are indicated by red and dashed red star respectively. Created with BioRender.com

Cyclin-dependent kinase 5 (CDK5 located at 7q36.1) was discovered thanks to its high amino acidic similarity with mouse CDK1 (58%) and human CDK2 (61%) as a CDK expressed in terminally differentiated neurons, and no more in the cell cycle.^33,34^ While most of the CDK family member’s activity relies on the interaction with specific cyclins, CDK5 activating subunits do not belong to cyclins family of proteins. The main regulatory subunits of CDK5 are p35 (encoded by *nck5a*),^234^ a protein with a neural cell-specific expression, and its truncated form, p25^nck5a^, with the full protein determined as the main interactor in crude brain extract (Fig. 5a).^235^ Further screenings of human hippocampus libraries identified another 39-kDa protein subsequently named p39 able to activate CDK5,^236^ suggesting that they may define a distinct family of CDKs activating proteins. Structural data, mainly from Musacchio lab, indicate that p35 and p25 lack the tandem copies of the cyclin-box fold (CBF) to bind CDKs and have instead a single CBF-like motif (CBFL) that is necessary for their interaction with CDK5.^237,238^ Additional cell fractionation experiments demonstrated that p35 and p39 are enriched in the membrane-bound fraction of cultured neurons due to the presence of a myristoylation signal, which is responsible for the recruitment of CDK5 to the membrane.^239^ Accordingly p25, the cleaved form of p35, lacks the myristoylation signal which in turn results in cytoplasmic hyperactivation of CDK5 that is associated with neurodegeneration^240^ (Fig. 5a).

As noted for cell cycle and transcriptional CDK also CDK5 kinase activity is tightly regulated not only by the binding with the regulatory subunits but also by specific post-translational modifications. As previously described, in cell cycle CDKs phosphorylation of Tyr and Thr at the N-terminus represents inhibitory modifications while phosphorylation of Thr in the T-Loop is necessary for CDKs fully activation. For CDK5 these phosphorylations are present but have a quite different effect on the regulation of kinase activity. The phosphorylation at Tyr15 is responsible for the stimulation of CDK5 activity, while the ones at Thr14 and Ser159 are responsible for the inhibition of the kinase. Accordingly, Ser159 is not a target of and CDK5 is not activated by the CAK complex.^241^ Another difference between CDK5 and cell cycle CDK regulation resides in the fact that CDK5 could be modified on Lys/Cys residues by acetylation (Lys33)^242^ and S-nitrosylations (Cys83 and Cys157).^243^ While acetylation reduces CDK5 activity, S-nitrosylations increase it, adding a different layer of CDK regulation. Of note, also p35 could be S-nitrosylated at Cys92 and this modification favors its degradation via proteasome, consequently reducing the CDK5 kinase activity.^244^ Therefore, CDK5 activity regulation represents a unique example among the CDK family reinforcing the idea that it could be considered evolutionarily and functionally the prototype of atypical CDKs (Fig. 5). Whether these unique ways of regulation are shared also by the other members of atypical CDKs, is something that could be worth to investigate in the future.

As mentioned above, CDK5 expression was found in neurons having minor effects on cell cycle progression and most studies focused their attention on the possible roles of CDK5 in regulating nervous system development. Early results demonstrated that knock out of CDK5 in mice resulted in perinatal mortality associated with unique lesions in the central nervous system. These phenotype were likely due to the ability of CDK5 to modulate neurons migration during development through the regulation of microtubules and cytoskeleton structure and organization.^245^ A relevant mechanism of CDK5-dependent neuron migration might be related to the ability of CDK5 to phosphorylate CRMP2 (Collapsin Response Mediator Protein 2). CRMP2 activity and interaction with microtubules are fundamental for neuron migration during cerebral cortex formation, as demonstrated by knock-in of not phosphorylable CRMP2 (together with knock-out of CRMP1 and CRMP4) in mouse models.^246^ Further experiments, using ferrets as in vivo models, demonstrated a role for CDK5 in radial migration of cortical neurons, which should be perfectly positioned to lead cortical folding. CDK5 knockout in the cerebral cortex of ferrets induces defective migration in cortical neurons, strongly impairing cortical folding.^247^

Kodani and collaborators analyzed the migration of neurons to the posterior cortex and discovered that loss of CEP85L (Centrosomal Protein 85) impairs CDK5 localization, thus causing centrosome and microtubule disruption.^248^

In the context of postsynaptic density, it has been proved also that CDK5 phosphorylation of Lipirn-α1 (a membrane phosphatase important for axon guidance) is fundamental for the maturation of excitatory synapses by regulation of PSD-95 (PostSynaptic Density protein 95), an anchor protein that plays an important role in synaptic plasticity and the stabilization of synaptic changes during long-term potentiation (LTP = a persistent increase in synaptic strength).^249^

Multipolar newborn neurons undergo migration toward the developing cerebral cortex, where they become bipolar and the involvement of CDK5 in this process has been proven in the past. More recently it has been proven that the expression of dominant-negative CDK5 in the embryonic brain stopped neurons migration and the cells remained round and multipolar.^250^ In this context the role of CDK5 is to phosphorylate N-Cadherin and the proposed combined activity of N-Cadherin and CDK5, in driving multipolar neuron migration and bipolar transition, has been proposed as a conserved role in neuronal migration.^251^

The position of neurons in the inferior olive and facial motor nucleus is also CDK5-dependent, and more recently Sasamoto and colleagues demonstrated that CDK5 knockout mouse embryos present aberrant formation of the olfactory tubercles, nucleus accumbes and ventral striatum.^252^ Conditional knockout of p35 and p39 in GABAergic neurons leads to the same phenotype, confirming that CDK5 activity is required for the correct formation of these structures.^253^ CDK5 takes part in the regulation of another process strictly associated to the nervous system: the circadian rhythm. It has been demonstrated that CDK5 phosphorylates PER2 and CLOCK, regulating their activity.^254^ Overall, vast amount of literature points to multiple roles for CDK5 in the normal development of the nervous system in particular by regulating neurons motility and differentiation. It is therefore not surprising that it is also deeply implicated in neurodegenerative diseases^255^ (see section “Roles of CDKs in Neurodegenerative diseases”).

Beside its importance in the neural compartment, CDK5 has been implicated in the regulation of several other processes. The deregulation of CDK5 activity in dopamine neurons disrupts autophagy, leading to hyperactivation of innate immunity and ultimately causing age-dependent neuronal death.^256^ Other studies have indicated an indirect role for CDK5 in the regulation of immune response regulating the expression of PD-L1 through FBXO22, or by stabilizing IRF2 and IRF2BP2, phosphorylation.^257,258^

Several studies have demonstrated the involvement of CDK5 in glucose homeostasis and a close relationship between insulin and CDK5. Insulin activates CDK5, which in turn regulates glucose-stimulated insulin secretion. Lalioti and colleagues showed that insulin-stimulated CDK5 activation plays a crucial role in GLUT4-mediated glucose uptake in 3T3-L1 cells through E-Syt1 phosphorylation.^259^ CDK5 also influences high glucose-stimulated insulin secretion by acting on Ca2+ channels, although no CDK5-dependent effects are observed under low glucose conditions.^260^

CDK5 activity is also crucial in regulating angiogenesis and lymphangiogenesis. Inhibition or downregulation of CDK5 reduces both endothelial cell motility and angiogenesis, primarily through the activity of the small GTPase Rac1.^261^ Similarly, endothelial-specific knockdown of CDK5 disrupts the normal pattern of lymphatic vessels and valve formation. This disruption leads to congenital lymphatic dysfunction and lymphedema through the regulation of Foxc2 (Forkhead Box C2) a member of forkhead family of transcription factors which may play a role in the development of mesenchymal tissues and that is associated with lymphedema development.^262^

Myogenesis, the process of skeletal muscle formation, is significantly influenced by CDK5 activity. In adult mouse muscle, CDK5 maintains stable expression, but its pattern varies during myogenesis. CDK5 expression peaks between 36 and 48 hours (early myogenesis) and decreases after 60 to 72 hours during myotube fusion. Overexpression of CDK5 in C2 myoblasts enhances differentiation, while its downregulation inhibits the onset of differentiation.^263^ During myogenesis, the Atypical protein kinase C zeta (PKCζ) phosphorylates p35 at Ser33, promoting calpain-mediated cleavage of p35 to its more stable form, p25. Inhibition of PKCζ reduces myotube formation and nestin reorganization in the cytoskeleton. Nestin, which determines the onset and speed of differentiation, also regulates the calpain-mediated cleavage of p35, thereby influencing CDK5 activity.^264^

Overall, although CDK5 is primarily implicated in the regulation of neurophysiology it seems to play also important roles in other adult tissues controlling for instance metabolism and myogenesis.

##### The PFTAIREs subfamily

The PFTAIRE family of cyclin-dependent kinases is characterized by its unique sequence motif and relatively recent evolutionary origin. With CDK14 being broadly expressed and CDK15 showing brain-specific expression, these kinases are still mostly understudied but could play important roles in cell cycle regulation and potentially specialized functions in the brain. More researches are needed to further elucidate their roles, regulatory mechanisms, and implications in health and disease.

**CDK14**

CDK14 (also known as PFTK1 located at 7q21.13),^265^ is a protein of 469 amino acids primarily expressed in the nervous system, as well as in the gastrointestinal, respiratory, and urinary tracts, with both nuclear and cytoplasmic localization (Human Protein Atlas).

Functional assays and two hybrid experiments suggested that CDK14 binds cyclin Y and cyclin D3 that could represent their activating binding partners.^266,267^ It also interacts with the CDK inhibitor p21 in a ternary complex with cyclin D3.^266^ Crucial for cyclin Y-CDK14 complex formation is the interaction with 14-3-3 proteins that could interact with phosphorylated cyclin Y, enhancing its interaction with CDK14,^268^ as observed also for the highly homolog CDK16 protein. Although the cyclin D3-CDK14 complex could phosphorylate in vitro the RB protein, its role in role in physiological cell cycle progression at G1/S transition remain elusive.^266^ Of note experimental evidence suggest that the cyclin Y-CDK14 complex acts upstream of Wnt signaling by phosphorylating the co-receptors for Wnt ligands LRP5/6 (Low-density lipoprotein receptor-related proteins 5 and 6) and increasing Wnt/β-catenin signaling seen at the G2/M transition of the cell cycle.^269^

**CDK15**

CDK15 (also known as PFTK2 located at 2q33.1) is a poorly characterized 384 amino acid protein highly homologous to CDK14 and CDK16. From an evolutionary perspective, the CDK15 appears to have a recent origin, as most animal species possess only one member of this family, which closely resembles CDK14. The emergence of CDK15 likely resulted from gene duplication events that allowed for functional diversification and specialization.^270^ Like other atypical CDKs, CDK15 is predominantly expressed in human adult organs and specifically in the gonads (both testis and ovary) and brain, with a cytoplasmic and membranous localization (Human Protein Atlas). The few data available on the possible biological roles CDK15 derive from initial studies in cancer models and suggest the involvement of CDK15 in the regulation of apoptosis and cell motility and will be reported in chapter 3.

##### The PCTAIREs subfamily

PCTAIREs, firstly cloned in 1992, are composed of roughly 500 amino-acid long proteins.^32,271^ Like other CDKs, PCTAIREs are well evolutionarily conserved and share high sequence homology with other CDKs, specifically to PFTAIRES (CDK14 and 15) (42-46%), CDK5 (58%) and 52-54% with canonical CDKs (CDK1, 2, and 3). Among PCTAIREs, only CDK16 structure is experimentally resolved and comparative analysis with CDK2 and CDK5, highlight the conservation of sequence level homology, also at structural levels.^272^ One of the main features of the CDK subfamily PCTAIRE is the replacement of the Serine residue by one of Cysteine in the consensus PSTAIRE motif, which is conserved in the early characterized CDKs. This observation suggest that this replacement could prevent the binding of PCTAIRE to cyclins or provide a unique binding site for other cofactors.^273^

**CDK16 an atypical CDK of differentiated adult tissue**

CDK16 (also known as PCTAIRE1, located at Xp11.3) was first identified in humans in 1992.^32^ It was the first member of the PCTAIRE family to demonstrate kinase activity toward MBP (Myelic Basic Protein).^270^ The kinase domain of CDK16 adheres to the classical bi-lobal architecture, flanked by unique N- and C-terminal extensions that contribute to the characteristic folding of the CDK family. CDK16 binds to cyclin Y (CCNY), which leads to its localization at the plasma membrane. This interaction alone may be insufficient to trigger full CDK16 kinase activity which might require the binding of an additional protein, likely a member of 14-3-3 protein family. Even if, several studies have shown that the interaction with 14-3-3 proteins can potentially increase CDK16 basal kinase activity, it remains uncertain whether this binding is stable, direct, and/or requires the 14-3-3 protein binding to a previously phosphorylated CDK16.^273–275^

More recently, cyclin Y-like 1 (CCNYL1) was identified as another cyclin capable of binding to and activating CDK16 and seems to have a higher binding affinity for CDK16 compared to CCNY, increasing the stability and kinase activity of the complex.^276^ Besides 14-3-3 proteins, CDK16 was shown to interact with p11 in the brain, although the functional implications of these interactions are not yet fully understood.^270,277^ Additionally, both PKA and CDK5/p35 complex can phosphorylate CDK16, thereby increasing its kinase activity.^273^

CDK16 is highly expressed in differentiated tissues such as the brain, testis, and skeletal muscles but displays a relatively ubiquitous expression profile compared to other members of the PCTAIRE family (Human Protein Atlas). Consistent with their expression patterns, the conditional knockout of CDK16 or CCNYL1 in male mice demonstrated that the CDK16-CCNYL1 complex is essential for the terminal differentiation of spermatocytes and proper spermatogenesis. Notably, no other obvious phenotypic differences were observed between the knockout mice and their control littermates.^274,276^

Beyond spermatogenesis, CDK16 has been implicated in various cellular functions mostly related to brain development, neuronal migration, and neurite outgrowth. This is evidenced by observations that forced nuclear targeting of CDK16 abolishes neurite outgrowth^278^ and that CDK16 regulates cytoskeletal rearrangement and neurite outgrowth through the modulation of CDK5 activity.^279^

CDK16 also participates in several cell death pathways, including apoptosis, senescence, and autophagy. Its roles in vesicular transport and actin cytoskeleton organization are relevant to autophagy, potentially positioning autophagosomes near lysosomes or balancing cellular fate between autophagy and apoptosis.^280^ Under autophagic stimuli, AMPK phosphorylates CCNY at Ser326, promoting the CCNY-CDK16 interaction and increasing CDK16 kinase activity.^281^ Through inducing autophagy, CDK16 could be associated with various diseases, including inflammatory, neurodegenerative, and tumorigenic conditions.^280,281^

In membrane trafficking, CDK16 interacts with the Sec23A protein subunit, part of the COPII complex involved in cargo transport from the endoplasmic reticulum (ER) to the Golgi apparatus.^282^ Both CDK16 and Sec23A interact with 14-3-3 proteins, which play a role in ER-to-Golgi transport.^283^ However, the exact mechanism of PCTAIREs in cargo transport remains unclear.

Lastly, CDK16 may regulate myogenic differentiation. Overexpression of CDK16 increases levels of known myogenesis markers, such as Myosin Heavy Chain (MHC) and Troponin C, while its silencing has the opposite effect.^278^

**CDK17: The least studied atypical CDK**

CDK17 (also known as PCTAIRE2, located at 12q23.1) is one of the least well-studied members of the CDK family. Similar to CDK16, CDK17’s kinase activity, when purified from the rat brain, is sensitive to high salt concentrations, suggesting the need for an activating subunit. However, no studies have definitively identified the cyclin binding partner of CDK17. It has been shown to interact with TRAP (Tudor repeat associated with PCTK2) and IK3-1/Cables1 (Cdk5 and Abl enzyme substrate 1), but none of these interactors could stimulate CDK17’s kinase activity toward the Histone H1 substrate in vitro.^273^

CDK17 is predominantly expressed in the cytoplasm and membrane of normal human tissues, although it can translocate to the nucleus in cultured cells. It is mainly found in terminally differentiated neurons, particularly in the hippocampal regions and olfactory bulbs.^273^ The physiological and pathological roles of CDK17 are poorly understood. Interestingly, CDK17, along with CDK18, has been identified as a potential inhibitor of the autophagic process, based on a screening of 354 human kinases that were constitutively activated through membrane recruitment by adding a myristylation signal.^284^ This supports the hypothesis that CDK17 could regulate vesicle internalization and trafficking in various contexts. Accordingly, experimental models show that CDK17 expression is strongly correlated with and significantly inhibits viral infections.^285^

**CDK18: a possible link between CDKs and water homeostasis**

CDK18 (also known as PCTAIRE3, located 1q32.1), is another underexplored CDK. It shares amino acid sequences in the helical α-C region of the kinase N-lobe with other PCTAIRE members, a region used by CDKs to recruit their respective cyclin partners. However, the specific cyclin partner for CDK18 remains elusive. The few available data suggest that CDK18 could interact with both cyclin A2 (CCNA2) and cyclin E2 (CCNE2).^286^ CDK18 kinase activity is promoted by interaction with cytoplasmic cyclin A2 for the phosphorylation of RB, which is further enhanced by PKA-mediated CDK18 phosphorylation at S127.^286^

CDK18 exhibits a limited pattern of expression, primarily observed in the brain but also present in the kidney, testis, heart, and intestine.^32,271^ Consistently, CDK18 was first described as a neuronal kinase that phosphorylates the TAU protein when overexpressed in the human brain. In mature tissues and cell lines, CDK18 predominantly localizes in the cytoplasm and at the plasma membrane and most information on CDK18 functions relates to neurological studies.^273^ CDK18 plays a role in oligodendrocyte precursor cells by promoting their differentiation through the activation of the mitogen-activated protein kinase ERK pathway in the remyelination process.^287^ Proteomic approaches have demonstrated that exposure to antidepressants in rats leads to upregulation of CDK18 in the hippocampal regions. Similarly, CDK18 expression is upregulated in the central nucleus of the amygdala region upon alcohol consumption.^273^ Its expression is further elevated in the Amyloid Precursor Protein (APP) Alzheimer mice model compared to non-transgenic mice, suggesting an important role in the pathogenesis of neuronal degeneration.^288^

Similar to CDK16, CDK18 interacts with the Sec23p subunit of the COPII complex, which is necessary for the export of secretory cargo from the endoplasmic reticulum. Silencing CDK18 or expressing a kinase-dead mutant dramatically impacts the function and regulation of the early secretory pathway.^282^

Recent studies have identified CDK18 as a crucial regulator of genome stability. Depletion of CDK18 increases endogenous DNA damage and chromosomal abnormalities. In response to replication stress, CDK18 promotes ATR-mediated signaling, regulating the phosphorylation level of RPA (Replication Protein A) proteins. CDK18 is suggested to interact with stress signaling proteins RAD9 and RAD17, contributing to their chromatin localization and preventing the accumulation of DNA damage by activating ATR-mediated genomic replication stress signaling.^289^

Finally, CDK18 controls the trafficking of the Aquaporin-2 (AQP2) water channel. It undergoes vasopressin-induced phosphorylation, finely regulating the abundance and localization of AQP2 in the plasma membrane, contributing to body water homeostasis. Perturbations in the vasopressin-AQP2 signaling pathway have been associated with nephrogenic diabetes insipidus (NDI). CDK18 also shows slightly higher expression in diabetic pancreatic islets compared to normal islets, supporting a possible link between CDK18 expression and the onset/development of diabetes.^273^

### CDKs in human pathologies

To describe the proposed roles and the observed deregulation of CDKs in human pathologies we divided this chapter in the following different paragraphs that summarizes the known and proposed roles of the three CDKs subfamilies in cancer onset and progression, neurodegenerative, cardiovascular and autoimmune diseases, metabolic disorder and inflammation and fibrosis.

#### Roles of CDKs in cancer

The role of CDKs, cyclins and CDK inhibitors in cancer has been widely addressed as expected by the fact that all six of originally identified hallmarks of cancer^290^ are directly or indirectly regulated by CDKs activity. For clarity we will refers to the most significant role of each CDKs following their membership to the three CDKs subfamilies (Table 2).

**Table 2:** The Role of CDKs in cancer

Tissue of origin	Oncogenic functions	Tumor-suppressive functions
Brain	CDK2-CDK3-CDK4-CDK6-CDK7-CDK14-CDK20	CDK10
Head & neck	CDK1-CDK3-CDK4-CDK7	
Thyroid	CDK13	
Lungs	CDK1-CDK2-CDK5-CDK7-CDK9-CDK20	CDK10
Breast	CDK1-CDK2-CDK4-CDK5-CDK6-CDK7-CDK8-CDK9-CDK11-CDK12-CDK13-CDK14-CDK19	CDK10-CDK12-CDK13
Liver	CDK1-CDK7-CDK9-CDK13-CDK14-CDK20	CDK10
Stomach	CDK2-CDK7-CDK8-CDK12-CDK14	CDK5-CDK8-CDK10-CDK12
Pancreas	CDK1-CDK5-CDK7-CDK8-CDK12-CD14	CDK8-CDK12
Colorectal	CDK1-CDK2-CDK3-CDK4-CDK7-CDK8-CDK10-CDK13-CDK19-CDK20	CKD8-CDK19
Reproductive and urinary system (female)	CDK1-CDK2-CDK7-CDK9-CDK11-CDK14-CDK20	CDK12-CDK13
Reproductive and urinary system (male)	CDK1-CDK2-CDK7-CDK8-CDK9-CDK12-CDK13-CDK14-CDK19	CDK8-CDK12-CDK13
Hematic and lymphatic circulation	CDK4-CDK6-CDK7-CDK8-CDK9-CDK11-CDK12-CDK13-CDK19	CDK8-CDK12-CDK13-CDK19
Bone and soft tissues	CDK2-CDK4-CDK6-CDK9-CDK11-CKD14	
Skin	CDK1-CDK3-CDK4-CDK6-CDK8-CDK9-CDK12	CDK8-CDK12-CDK13

Summary of the role of CDKs in cancer, categorized by the tissue of origin of the tumor and the oncogenic or tumor-suppressive functions of these kinases. CDKs have direct involvement in specific pathways and are often overexpressed or downregulated in tumors compared to healthy tissues. This table focuses exclusively on CDKs, excluding the various co-factors involved in their regulation. Detailed references related to the specific CDKs and their associations with different tumors are provided in the corresponding paragraph

Created with BioRender.com

##### Roles of cell cycle CDKs in cancer

**CDK1**. CDK1 plays essential roles in cell cycle progression, with its depletion resulting in cell death in vitro and embryonic lethality in vivo. In the context of cancer CDK1, by phosphorylating multiple factors, plays a major role in promoting cell transformation and tumorigenesis. It is fundamental in managing chromosomal instability, one of the hallmarks of cancer and below we report some typical examples of its involvement in different types of human cancers. CDK1 expression is significantly increased in several tumor types compared to normal tissues, particularly in breast, colon, lung, prostate, head and neck cancer, liver carcinoma, pancreatic adenocarcinoma, and glioblastoma.^291,292^ At genomic levels CDK1 is amplified in 7% of uterine carcinosarcoma cases and mutated in 3% of melanoma tumors. In tumor cells CDK1 phosphorylates numerous oncogene and tumor suppressor proteins, and several upstream modulators can positively or negatively influence CDK1 activation, amplification, transcription, and expression. A pan-cancer bioinformatic analysis demonstrated coherent expression between CDK1 and cellular pathways often dysregulated in cancer, including cell-cycle-related targets, the G2/M DNA damage checkpoint, mTORC1 signaling, MYC target genes, and the p53 pathway.^291^

Despite its multi-functional role in controlling cell cycle progression and division, CDK1 is considered the master regulator of mitosis. Blocking aberrant mitosis is critical to prevent chromosomal instability (CIN), a hallmark of cancer. Therefore, in general, chromosomally unstable tumors had a high presence of centrosomal and mitotic proteins, including CDK1, which is overexpressed together with cyclin B1 and B2. Centrosomal amplification, along with increased CDK1 activity, leads to aberrant mitotic spindle and multiple spindle pole formation, resulting in CIN due to abnormal cell division and aneuploidy, particularly in p53-defective cells.^293^

Genomic instability is tightly linked to the activity of telomeres length regulation by telomerase (hTERT) activity. Shortening of telomeres (telomere attrition) results in CDK1-mediated cell cycle arrest and operate as a tumor suppressor pathway.^294^ On the other hand CDK1 could directly phosphorylate hTERT and impact on tumor formation and tumor aggressiveness.^295^

Increased ability to invade is also another hallmark of cancer potentially controlled by CDK1 by modulating several members of the Hippo pathway, thereby regulating neoplastic transformation,^296^ EMT and cell migration and invasion.^297^

The cyclin B1-CDK1 complex may contribute to tumorigenesis by promoting cell proliferation and survival through direct inhibitory phosphorylation of FOXO1, blocking pro-apoptotic signals^298^ or by directly phosphorylating both pro-caspase 8 and 9, resulting in the abrogation of apoptotic signals and shielding cells from death.^299,300^ This phenomenon has significant implications for cell death upon chemotherapeutic drug treatment. For instance, in colorectal carcinoma (CRC) with the BRAF V600E mutation (8% of CRC cases), treatment with CDK1 inhibitors significantly enhances the therapeutic efficacy of MEK inhibitors by reactivating caspase 8 activity.^301^

Finally, CDK1 could be implicated in the progression of specific cancer as observed in prostate carcinomas where it phosphorylates Androgen Receptor (AR), which results in decreased transactivation upon androgen stimuli. CDK1-phosphorylated AR serves as a negative prognostic marker independent of known clinical parameters.^302,303^

**CDK2**. Genetic alterations in CDK2 are rarely identified in human cancers. However, the copy number loss or the mutation of its principal CKI p27 along with their decreased expression, and the overexpression of cyclin E1, frequently occur and correlate with increased CDK2 activity and poor prognosis.^293,304^ Cyclin E overexpression is observed in a variety of cancers, including breast, lung, cervix, endometrium, gastrointestinal cancers, lymphoma, leukemia, sarcomas, and adrenocortical tumors.^293,304^ Cyclin E gene amplification has been reported in primary endometrial, ovarian, colorectal, breast, and gastric cancers with frequencies ranging from approximately 2% to 20%.^305^ Mutations in p21 are rare, but this protein plays a crucial role in many dysregulated pathways in cancer, contributing to aggressiveness, stemness, drug resistance, and invasiveness. Studies have shown a strong correlation between low p27 expression levels and poor prognosis in various cancers.^305^ Overall, the data from primary tumors suggest that hyperactivation of CDK2 is strongly associated with tumor progression and is generally related to functional deregulation rather than its direct genomic abnormalities.

According to the above observation other CDK2 regulators are often altered in human cancer. Cables, which promotes WEE1-dependent inhibitory phosphorylation of CDK2 on Y15, is inactivated in 50–60% of primary colon and head and neck tumors. Additionally, CDC25A and CAK, which sustain CDK2 activation, are upregulated in a range of tumors, particularly in breast cancer.^306,307^ Cyclin E overexpression is a driving event in high-grade serous ovarian cancer (HGSOC) and define a subgroup of HGSOC constitutively resistant to platinum-based chemotherapy. In tumors with very high cyclin E1 expression, the cyclin E1-CDK2 complex mediates cell cycle progression, chromosomal instability, and genomic instability, resulting in chromosome mis-segregation, polyploidy, and genomic deletions.^308^ Inhibition of CDK2 in HGSOC tumors may be crucial to block tumor growth and prevent the development of platinum-resistant recurrences.

Yet, CDK2 dependency could be tumor dependent. For instance, colon carcinoma cells can efficiently proliferate without CDK2, while in other tumors, downregulation or inhibition of CDK2 in cell lines prevents proliferation.^309^ One possible explanation for their different dependency could be the presence of a fully functional RB protein. In tumors driven by MYC overexpression, CDK2 activity is vital for preventing senescence and enabling cancer cell immortalization. For instance, in MYCN-amplified but not in non-MYCN-amplified neuroblastoma cell lines, selective silencing of CDK2 leads to apoptosis associated with the upregulation of p53 target genes.^310^ Similar results were obtained in KRAS-mutated lung cancer, where inhibition of CDK2 results in anaphase catastrophe and apoptosis, reducing the growth of lung cancer xenografts.^311^

CDK2 plays a pivotal role in the development and progression of breast and prostate cancer through its ability to phosphorylate androgen, estrogen, and progesterone receptors. In prostate cancer, CDK2 overexpression is associated with recurrence risk, and its expression is more than double in metastases compared to primary tumors.^312^ Glioma tumors display high dependence on CDK2, with increased expression potentially due to copy number alterations, altered methylation, or enhanced transcriptional activation. In glioblastoma (GBM), CDK2 expression is significantly enriched and functionally required for tumor proliferation. High CDK2 expression is associated with poor prognosis in GBM patients, and CDK2 induces radioresistance in GBM cells, while its downregulation increases apoptosis in combination with radiotherapy.^313^

**CDK3**. CDK3 is poorly expressed in most human tissues, mainly controlling the G0-G1 transition and RB phosphorylation in early G1. Despite the limited literature on CDK3’s role in cancer compared to other cell cycle-related CDKs, emerging studies have unveiled intriguing functions. The overall picture emerging from these studies, suggests that CDK3 is mainly overexpressed in human tumors rather than genetically modified. The mechanisms behind this overexpression are not fully understood, but its involvement in metastasis,^314^ and cell transformation and tumor growth^315^ highlights CDK3’s potential as a target for cancer therapy.

An interesting observation that might merit to be confirmed is the association of CDK3 overexpression due to gene rearrangements with BRCA1 loss of heterozygosity (LOH) in breast cancer, a tumor in which CDK3 activity has been linked to tamoxifen resistance.^316^

**CDK4 and CDK6**. Several human tumors exhibit genetic or epigenetic alterations in the cyclin D-CDK4/6-RB pathway. Common events in cancer include the overexpression of cyclin D types cyclins and the loss of CDK inhibitors (especially p16INK4a, p15INK4b, p21 and p27), leading to CDK4 and CDK6 hyperactivation.^293,317^ Being the use of CDK4/6 inhibitors now approved for clinical use in some type of cancer, an enormous amount of data, also already reported in excellent specific reviews, have been produced in the last years and we refer to them for more comprehensive and detailed information (see for example^318–320^). Here we provide a schematic overview on their contribution in tumor progression, since CDK4 and CDK6 have distinct and overlapping functions in tumor development, affecting stemness, angiogenesis, transcription, metabolism, and immune modulation. We therefore have tried to summarize below the CDK4 and CDK6 shared activities in cancer progression and at the same time highlighting which are the functions that could be considered specific for each kinase.

**Common activities shared by CDK4 and CDK6** in cancer have been mostly identified and confirmed evaluating the roles of CDK4 and CDK6 specific inhibitors and, among them the principal is indubitably to promote cell proliferation by directly phosphorylating RB and indirectly through mTORC1 activation.^318,319^

Another highly relevant activity for cancer progression is the ability of CDK4 and CDK6 to suppress senescence by phosphorylating FOXM1, maintaining cell cycle progression and preventing tumor suppressive mechanisms. On the other side, however, the senescent phenotype induced by CDK4 and CDK6 inhibition, results in the presence of metabolically active senescent cells that produce proinflammatory cytokines and promigratory factors, collectively termed the senescence-associated secretory phenotype (SASP).^65^ As a consequence, CDK4 and CDK6 inhibition might modulate the tumor microenvironment enhancing immune cell infiltration through SASP. Of note, recent observations suggest that they also play a role in immune system activation, reducing regulatory T cells and promoting anti-tumor immune responses via the cGAS-STING pathway.^319,321^

**CDK4**. CDK4 is amplified or overexpressed in various epithelial malignancies, sarcomas, gliomas, breast tumors, lymphomas, and melanoma.^293,317^ A specific mutation identified in melanomas, CDK4 R24C, renders CDK4 insensitive to INK4 inhibition and correlates with tumor development. Mice with this mutation develop diverse malignancies (e.g sarcomas and pituitary adenomas) but require a carcinogen to develop melanomas.^322^

CDK4 is crucial for maintaining tumorigenic potential in breast cancer, and its absence or inhibition can prevent the development of mammary carcinomas triggered by the ERBB2 oncogene as also observed for the activating binding partner cyclin D1.^323,324^ These observations highlight the crucial role of cyclin D1/CDK4 complex as a sensor of oncogenic pathways activated by tyrosine kinases receptors. This concept is reinforced by the notion that CDK4 is able to counteract the tumor suppressor activities of TGF-β pathway activation by phosphorylating and inhibiting Smad3, contributing to uncontrolled cell proliferation.^93^

CDK4 participates in epigenetic regulation by phosphorylating MEP50, which enhances the activity of protein arginine-methyltransferase 5 (PRMT5).^325^ This mechanism is implicated in cancer progression and resistance to CDK4/6 inhibitors through the modulation of CUL4 expression, a component of the E3 ubiquitin-ligase complex, direct arginine methylation of p53 that suppresses the expression of antiproliferative and pro-apoptotic p53 target genes and by altered the pre-mRNA splicing of MDM4, a known negative regulator of p53.^326^ These data clearly link CDK4 to the epigenetic regulation of genes expression and functions in cancer.

The fact that CDK4 is strongly implicated in the regulation of protein stability is also supported by the observation that it regulates PD-L1 protein stability. This observation opened to important clinical application aiming at using combination treatment with CDK4/6 inhibitors and PD-1-PD-L1 immune checkpoint blockade to improve the therapeutic options for cancer patients.^327^

**CDK6**. CDK6 has been more frequently involved in the progression of hematological malignancy being altered in leukemia, lymphoma, and B-lymphoid malignancies due to chromosomal translocation involving the 7q21 locus and as critical a mediator of Notch signaling in T-cell acute lymphoblastic leukemia (T-ALL) and in MLL-rearranged leukemias, where it blocks myeloid differentiation.^328,329^ It is also hyperactivated or rearranged in various malignancies, including sarcoma, melanoma, breast cancer, glioma, and medulloblastoma.^330,331^

In human cancers the roles of CDK6 extend beyond cell cycle regulation, involving transcriptional regulation and interaction with various proteins. By phosphorylating the transcription factors NFY and SP1 and inducing the transcription of a number of genes such as PRMT5, PPM1D and MDM4, which negatively regulate p53 functions and overall promoting oncogenesis.^332^

CDK6 might also promote alteration of the local microenvironment by interacting in cancer cells with the transcription factor NF-kB, eventually stimulating the production of inflammatory-related cytokines and chemokines. On the other side, by binding and blocking the activity of the transcription factor RUNX1, CDK6 can prevent myeloid cells differentiation.^333^ CDK6 also impact on local microenvironment by regulating tumor angiogenesis in concert with STAT3 to induce p16^INK4a^ expression, or with AP-1 transcription factors and upregulate VEGFA, promoting tumor vascularization in a D-type cyclins independent manner.^334^

A relevant role of CDK6, likely in complex with cyclin D3, is its involvement in the DNA damage response through the regulation of ATR that opened the way to consider the use of CDK4 and CDK6 inhibitors association with chemotherapy.^66^ Also in complex with cyclin D3 CDK6 affects metabolic pathways in T-ALL by phosphorylating glycolytic enzymes, redirecting glycolytic intermediates, and increasing reactive oxygen species and apoptosis upon inhibition.^335^

Overall, the different activities of CDK4 and CDK6 described in human cancer directly link deregulation of G1-S phase transition to the control of DNA damage and DNA Damage Response and the modification of tumor microenvironment, giving a clear molecular explanation of the success of CDK4 and CDK6 inhibitors in the treatment of human cancers.

##### Roles of transcriptional CDKs in cancer

Similar to cell cycle CDKs, it is not surprising that transcriptional CDKs could also be altered and implicated in driving and maintaining cancer cell growth. Transcription cycles proceed through distinct steps: initiation, pausing, elongation, and termination all of which are tightly controlled by the coordinated action of transcriptional CDKs (tCDK) and their cyclins. Consequently, oncogenic alterations in tCDK can modify not only the expression of specific genes but also globally impact on transcriptional programs.^141^ Moreover, clusters of enhancers, known as “super-enhancers” (SEs), drive the expression of genes that define cell identity. Dysregulation of SEs is common in cancer, with super-enhancers often acquired at oncogene drivers (e.g., MYC) during tumor pathogenesis.^336^ These alterations lead to a transcriptionally dysregulated state in cancer, termed “transcriptional addiction,” resulting in a disproportionate dependency on various components of the core transcriptional machinery, including tCDKs.^337^ This phenomenon suggests that cancer cells may be more responsive than their normal counterparts to transcriptional inhibition. These dependencies provide important opportunities for developing new therapeutic approaches, using tCDKs both as pharmaceutical targets and biomarkers, as supported by the recent development of a large number of small-molecule inhibitors.^140,338^

**CDK7**. Genomic alterations in CDK7 have not been widely reported in cancer. However, a pan-cancer study across different epithelial tumor types revealed significant recurrent loss-of-function alterations in CDK7, including recurrent copy-number loss in four epithelial cancer types: ovarian serous, prostate and esophageal carcinoma and cholangiocarcinoma. Recurrent CDK7 deletion was also identified in triple-negative breast cancer (TNBC).^339^ On the other side, several studies have shown elevated CDK7 expression in tumor tissues compared to normal counterparts across various neoplasms, including but not limited to breast cancer, correlating with poor prognosis. Associations between CDK7 and reduced survival have been observed in gastric cancer, ovarian cancer, oral squamous cell carcinoma, hepatocellular carcinoma, and glioblastoma.^340^

Targeting CDK7 has shown dramatic effects on cancer cell proliferation, migration, stemness, and drug resistance across several malignancies such as breast cancer, lung cancer, colorectal cancer, leukemia, and many others.^341^ Inhibition or depletion of CDK7 primarily results in cell cycle arrest, reduced proliferation, and suppression of crucial cancer CDK7-dependent gene transcription, in accord with is double role in cell cycle progression (as CAK) and transcriptional regulation.^342^ CDK7 inhibition or silencing by different approaches suppresses cell proliferation, increases the G2/M cell population, and promotes apoptosis in different cancer models.^156,343^ Consistent with its CAK function, selective CDK7 inhibition (YKL-5-124 or THZ1) diminished CDK2 phosphorylation and/or reduced cell cycle and E2F-related gene expression in different models of human cancers, partially by impairing SE-associated gene expression.^344,345^

Based on the notion of “transcriptional addiction,” targeting transcription regulators, including CDK7, has emerged as a powerful strategy to attenuate cancer dependency, particularly in cancers that rely on SE-driven genes (e.g., MYC, RUNX2, SOX2, SOX9). Several studies have demonstrated that SE-associated genes are particularly downregulated in cancer cells treated with CDK7 inhibitors.^340^ For example, neuroblastomas driven by MYCN amplification, which induces aberrant SEs, are disproportionately vulnerable to CDK7 inhibition.^346^ Similarly, the oncogenic transcription factor RUNX1 in T cell acute lymphoblastic leukemia (T-ALL) is driven by SE and repressed by CDK7 inhibition. SE-driven genes, such as MYC, are particularly upregulated in cancer and are challenging to target directly, but this difficulty might be overcome by targeting CDK7.^347–349^ Studies in multiple types of cancer support this hypothesis.

In addition to transcription factors, CDK7 regulates multiple nuclear hormone receptors to promote their full activation. Metastatic castration-resistant prostate cancer (CRPC) is primarily connected to androgen receptor (AR)-dependent transcription and CDK7 by phosphorylating MED1 (a component of the Mediator complex that binds to multiple nuclear receptors) enhances its interaction with AR and AR-driven oncogenic transcription.^350^ Accordingly, selective CDK7 inhibition (CT7001) in CRPC cells, induces cell cycle arrest and activates p53-induced apoptosis also reducing the growth of CRPC xenografts and significantly augments the growth inhibition achieved by the antiandrogen enzalutamide.^351^

Similarly, in breast cancer CDK7 regulates Estrogen Receptor (ER) activity and the positive correlation between ER mRNA expression and upregulated levels of CDK7/cyclin H/MAT1 is associated with shorter Overall Survival (OS) of ER-positive breast cancer patients.^307^ Targeting CDK7 with specific small molecular inhibitors has shown efficacy in ER+ breast cancer cells by inhibiting proliferation, inducing apoptosis, and reducing the growth of breast cancer xenografts in murine models.^341^ ER mutations, especially in the ligand-binding domain (LBD), lead to its constitutive activation even in the absence of estrogen, contributing to endocrine therapy resistance. CDK7 inhibition (THZ1) impacts both wild-type and LBD-mutant ER, decreasing CDK7-mediated phosphorylation and expression of ER-dependent genes, thereby reducing cell proliferation in vitro and in vivo.^352–354^ Importantly, CDK7 inhibitors seem effective not only as monotherapy but also in combination with endocrine therapy (e.g. fulvestrant, tamoxifen) in vitro and in human breast cancer xenograft models.^343,354,355^

Interestingly, triple-negative breast cancers (TNBC), which display higher aggressiveness, advanced genetic complexity, and a characteristic gene expression program compared to hormone receptor-positive breast cancers, are exceptionally dependent on CDK7.^356^ TNBC shows a profound dependence on CDK7, which mediates this transcriptional addiction to a specific cluster of oncogenic genes, such as MYC, SOX9, FOXC1, EGFR, and FOSL1, promoting cell proliferation, migration, and stemness. CDK7 inhibition can limit these malignant features, as observed in patient-derived xenografts (PDXs) of TNBC.^341,356^ CDK7 expression is also increased in human epidermal growth factor receptor 2 (HER2) breast cancers, where it might act as a transcriptional cofactor, inducing dysregulated gene expression favoring resistance to HER2 inhibition. Dual treatment combining HER2-targeted therapy (Lapatinib) with the CDK7 inhibitor THZ1 strongly inhibits HER2+ breast cancer cell growth and increases apoptosis in cancer cells that exhibit resistance to HER2-targeted therapies.^357^

**CDK8 (and CDK19)**. As an important factor in transcriptional regulation, CDK8 dysregulation is closely related to cancer development. However, CDK8 possess both oncogenic and tumor-suppressive roles in different contexts, due to its function as modulator of transcription factor activity and as a regulator of Pol II- Mediator complex interactions.^141^ In contrast to CDK8, scarce information is available about unique CDK19 roles in cancer, and whether it compensates for loss of CDK8 remains largely unknown. As aforementioned, pharmacological inhibition or knockdown experiments have suggested that CDK8 and CDK19 control different sets of target genes.^165,358^ Several CDK8 pharmacological inhibitors have been developed,^171,359^ which are expected also to target CDK19, however reducing the capability to discern between CDK8 and CDK19 unique functions in cancer and leading to high toxicities at therapeutic dose levels as dual inhibitors.^360^

As reported for other tCDKs, with the exception of CDK12, mutations in CDK8 and CDK19 genes are rare. However, CDK8 is gained or amplified in 47% of analyzed colorectal adenocarcinoma (CRC) patient samples. This observation aligns with other studies reporting gains of the CDK8-associated 13q12.13 genomic region up to 60% of colorectal cancers.^361,362^ Further research showed that CDK8 levels increased in patients with advanced CRC stages (III/IV), suggesting the possible involvement of CDK8 in the advanced stages of CRC development and progression. Interestingly, in CRC CDK8 amplification is associated with the specific activation of the WNT-β-catenin pathway, rather than global transcription deregulation.^141,363^ High levels of CDK8 protect β-catenin/TCF-dependent transcription from repressive inhibition by E2F1 and correlates with high nuclear β-catenin localization and pathway activation.^363–365^ Consistently, CDK8 silencing or inhibition can reduce colon cancer cell proliferation, WNT target gene expression and tumor burden in xenograft models.^359,366,367^ Other reports suggest that CDK8 could also promote colon cancer metastatic growth to the liver and inhibition of CDK8 could suppress CRC metastatic growth, indicating the therapeutic potential of CDK8 inhibitors in CRC.^368^

As components of the Mediator complex, CDK8 (and CDK19) targeting disrupts Mediator-controlled transcription. SE-associated genes demonstrate a disproportionate sensitivity to CDK8 and/or CDK19 loss compared to genes associated with canonical enhancers. Unlike other tCDKs, CDK8/CDK19 act as potent repressors of SE-associated gene expression in CRC.^360^ Consistently, in CRC xenograft models, pharmacological inhibition of CDK8 and CDK19 resulted in gene expression profiles consistent with increased super-enhancer activity at MED1-marked associated genes.^360^

CDK8 is upregulated in breast cancers, associated with tumor progression and high expression of CDK8 predicts shorter relapse-free survival of breast cancer patients.^369^ In breast cancer Xu and colleagues identified CDK8 as a key regulator of G2/M transition, polyploidy, cell migration, and proliferation through the control of the ubiquitin ligase Skp2 and histone variant macroH2A1 (mH2A1) axis. CDK8 may control the cell cycle inhibitor p27 by driving its phosphorylation at the T187 residue, creating a priming site for Skp2-mediated p27 ubiquitination and degradation, thereby promoting cell cycle progression and tumorigenesis.^369^ Pharmacological (Senexin A and B) or genetic inhibition of CDK8 suppresses both the transcriptional (e.g., GREB1 and EGR3 genes) and mitogenic effects of estrogen in ER-positive breast cancer cells, partially suppresses HER2+ breast cancer tumor growth and potentiates the effects of HER2-targeting drugs, overcoming lapatinib resistance.^370^

CDK8 is an oncogenic driver also in hematological malignancies. As in CRC, CDK8 and its paralog CDK19 restrain the increased activation of key super-enhancer-associated genes, affecting global gene expression in acute myeloid leukemia (AML) cells that depend on precise super-enhancer-associated gene expression, and CDK8 inhibition has shown potent effects.^371^ In myeloproliferative neoplasms and JAK-mutated AML, CDK8 phosphorylates STAT1, leading to constitutive activation of its transcriptional program. CDK8 inhibition upregulates STAT1-target genes and decreases cell viability in JAK-STAT dependent myeloproliferative neoplasms and AML.^372,373^ CDK8 is indispensable for the maintenance of established leukemic cell lines, including BCR-ABL+ leukemias (B-ALL). Loss of CDK8 in leukemia mouse models significantly enhances disease latency and prevents disease maintenance, specifically deregulating the mTOR signaling pathway. A small molecule that combines mTOR inhibition and degradation of CDK8 (YKL-06-101) induced cell death in human leukemic cells, representing a potential therapeutic strategy for treating acute lymphoblastic leukemia (ALL) patients.^374^

**CDK9**. Genomic alterations in the CDK9 gene are relatively uncommon in human cancers. However, CDK9 (or globally the P-TEFb complex) is commonly overactive in many hematological and solid cancers through several mechanisms, aberrantly enhancing Pol II processing.^177^

The aberrant recruitment of P-TEFb complex frequently occurs through its interaction with dysregulated transcriptional activators, such as c-MYC, one of the most frequently amplified oncogenes in human tumors.^358^ As a general transcription factor, c-MYC can recruit CDK9/P-TEFb to transcription start site proximal regions through physical interactions, facilitating the transition from pausing to elongation processes. Overexpression of c-MYC can consequently lead to transcriptional amplification in cancer cells.^375^

c-MYC is frequently overexpressed in cancer either by gene amplification and copy number alterations or by the activity of cancer-specific super-enhancers, which require both BRD4 and CDK9/P-TEFb.^141,358^ In particular, P-TEFb and c-MYC dysregulation have been implicated in the development of hematological malignancies, such as large B-cell lymphoma where CDK9 inhibition induces apoptosis of lymphoma cells.^177^ Dysregulation of P-TEFb/CDK9 is also common in B-cell acute lymphoblastic leukemia (ALL) and acute myeloid leukemia (AML) in infant and adult myeloid leukemias, which carry rearrangement of MLL1 (Mixed-Lineage Leukemia 1) gene. MLL1 gene rearrangements often involve SEC complex partners (AF4, AF9, ENL, and AF10), which recruit P-TEFb and other elongation factors.^141^ MLL1 fusion chimeras continuously recruit P-TEFb to MLL1 target genes, such as HOXA clusters, which regulate hematopoietic stem cell identity and self-renewal. MLL1-rearranged malignancies exhibit specific CDK9-dependent gene expression features typical of hematopoietic progenitor cells.^376–378^ Accordingly, pharmacological inhibition of CDK9 reduces disease progression and improves survival in mouse models of MLL-driven and AML malignancies.^177^

P-TEFb dysregulation is also linked to oncogenesis, progression, and resistance in many solid tumors, including breast,^379^ prostate,^153^ and ovarian cancers,^380^ melanoma,^381^ and osteosarcoma.^382^ We will briefly illustrate some exemplifying reports in cancer settings.

Breast cancers, regardless of subtype, depend on continuously activated gene expression driven by P-TEFb. CDK9-mediated overexpression of c-MYC is associated with estrogen receptor (ER) independent growth in breast cancers resistant to endocrine therapy.^379^ High c-MYC and CDK9 levels correlate with multiple endocrine therapy-resistant MCF7 cancer cell lines. CDK9 inhibition induces apoptosis and inhibits cell growth in both hormone therapy-sensitive and resistant ER-positive breast cancer cell lines. CDK9 inhibition (e.g., AZD4573) synergizes with palbociclib in ER-positive models, suggesting a combination strategy to overcome endocrine therapy resistance.^383^

In prostate cancer, P-TEFb and BRD4 directly interact with the androgen receptor (AR) to mediate expression of important target genes, such as prostate-specific antigen (PSA). CDK9 phosphorylates AR at Ser81, influencing its transcriptional activity, stability, and nuclear localization. This phosphorylation enhances the recruitment of p300 and BRD4, releasing P-TEFb and maintaining AR target gene transcription.^384^ Inhibition of AR signaling, is standard treatment for advanced prostate cancer, but resistance often develops, leading to castration-resistant prostate cancer (CRPC). CRPC could express the constitutively active AR splicing variants, especially AR-V7, enable ligand-independent AR signaling and anti-androgen resistance.^153^ Blocking AR-V7 phosphorylation with CDK9 inhibitors impairs its pro-metastasis function.^385^

In ovarian cancer, high expression of CDK9 correlates with poor prognosis. CDK9 expression is significantly lower in primary cancer tissues compared to paired metastatic and recurrent tissues.^380^ CDK9 inhibition suppresses RNA transcription elongation, induces apoptosis, and reduces proliferation, spheroid growth, clonogenicity, and migration in ovarian cancer cells.^380^ CDK9 has a kinase-dependent role in DNA repair, including homologous recombination (HR) and replication stress response, which mediate resistance to therapies like chemotherapy and PARP inhibitors, commonly used in the treatment of ovarian cancer patients.^185^ ALK phosphorylates CDK9 at Tyr19, promoting HR repair and PARP inhibitor resistance. Phospho-CDK9-Tyr19 increases CDK9 kinase activity and nuclear localization, stabilizing P-TEFb and activating Pol II-dependent transcription of HR-repair genes. Phosphorylation of ALK is higher in PARP inhibitor-resistant ovarian and breast cancers compared to sensitive counterparts.^386^

**CDK10**. CDK10 is increasingly recognized to be implicated in either cancer progression. Although it has been identified as a potential oncogene in colorectal cancer (CRC), where its high expression is a prognostic factor of poor survival and correlates with tumor growth and reduced chemosensitivity by upregulating the antiapoptotic Bcl-2,^387,388^ it seems to act mainly as tumor suppressor in different type of cancer including breast,^389^ lung,^390^ and thyroid cancer,^391^ hepatocellular carcinoma (HCC)^392^ and glioma.^393^

Most of the available studies focused on the tumor suppressor role of CDK10 in breast cancer where it correlates with disease progression, poor survival, and lymph node metastasis.^394^ CDK10 reduced expression can be ascribed to copy number loss as observed in a Swedish cohort or to methylation of CpG islands in CDK10 promoter, which correlates with shorter overall survival in breast cancer patients.^389,395^ Low CDK10 expression leads to increased ETS2-driven transcription of c-RAF, resulting in MAPK pathway activation and loss of tumor cell sensitivity to endocrine therapy.^395^

Hypermethylation of CDK10 promoter was also confirmed with high frequency (57.5%) of nasopharyngeal carcinomas samples and cell lines and low CDK10 expression is also correlating with advanced TNM stages in HCC samples where its expression could regulate chemosensitivity to cisplatin and epidoxorubicin.^392,396^

Finally, tumor suppressor activity of CDK10 could be related to its ability to regulate epithelial-to-mesenchymal transition (EMT) eventually inhibiting metastasis formation.^390,391,393^ Indeed, CDK10 downregulation correlates with increased metastatic potential in different tumors and its reduced expression activates EMT driven by the transcriptional factor Snail, thereby facilitating glioma metastasis.^393^ Similarly, reduced expression of CDK10 could promote metastasis in lung and thyroid adenocarcinoma via EMT regulation and Matrix metalloproteinases-2 and -9 over production.^390,391^ These insights potentially elucidate a mechanism for CDK10’s role in tumor suppression.

**CDK11**. The upregulation of both CDK11 and cyclin L1 (CCNL1) expression is commonly detected across various cancer types including osteosarcoma, liposarcoma, multiple myeloma, esophageal squamous cell carcinoma, breast cancer, and ovarian cancer.^397^ Amplification of CCNL1 has been correlated with unfavorable clinical outcomes in head and neck squamous carcinomas.^397^ Additionally, CCNL1 has emerged as a substrate of SCF^FBXW7^, an E3 ligase complex involved in proteasome-dependent degradation.^397^ Mutations in FBXW7 lead to increased CCNL1 expression, consequently enhancing CDK11 activity and accelerating mitotic progression.^398^ These observations underscore the significance of CDK11/cyclin L1 complexes in cancer progression.

Large-scale cancer screenings have identified CDK11 as a pro-survival gene in cancer, highlighting the reliance of cancer cells on CDK11 expression for their viability.^399^ In osteosarcoma models with siRNA resulted in diminished proliferation and induced apoptosis, concomitant with a reduction in anti-apoptotic markers. The Core-binding factor subunit beta (CBFb), a transcription factor involved in osteogenesis, has been identified as a target of CDK11^p110^ in osteosarcoma.^400^ This interaction results in increased expression of CBFb, which is associated with worse outcomes in terms of disease-free survival and metastatic potential.^400^

In breast cancer, immunohistochemical analysis displayed a relevant high expression of CDK11 in TNBC tissues, compared to normal breast tissues and its downregulation diminished tumor growth and reduced cell proliferation.^401^ However, it is necessary to consider the specific roles played by distinct CDK11 isoforms in oncogenesis. Diminished expression of CDK11^p58^ has been correlated with poorer disease-free survival in breast cancer where CDK11^p58^ acts as a tumor suppressor.^402^ Its overexpression in ERα+ breast cancer hinders growth, angiogenesis, and invasion by facilitating ERα proteasome-dependent degradation.^402^ In contrast, CDK11^p110^ is highly expressed in breast cancer tissues where positively correlates with higher histological grade and TNM stage.^403^ Knockdown of CDK11^p110^ in vitro reduced breast cancer cell survival and proliferation through induction of apoptosis and G1 cell cycle arrest.^403^ Similarly, in pancreatic ductal adenocarcinoma, CDK11^p110^ protein is stabilized via N-glycosylation and this stabilization promotes gemcitabine resistance, as well as enhanced invasion and migration capabilities.^404^ The differences in CDK11 isoforms’ roles underscore that they can exert opposing effects on cancer cell growth and proliferation via distinct molecular mechanisms, that can be exploited for therapeutic purposes.

**CDK12**. Increasing evidence highlights CDK12’s significant role in cancer development.^405^ The concept of “transcriptional addiction” describes cancer cells’ reliance on specific transcriptional regulators to maintain altered transcriptional programs. CDK12 appears to be crucial in this context, given its role in controlling DDR signaling genes, mRNA splicing, and translation processes.^405^ Dependency analysis from the Cancer Dependency Map (DepMap) project revealed that 30% of the 1,070 cancer cell lines examined depend on CDK12 expression.^406^

CDK12 alterations include both loss-of-function and gain-of-function mutations, which can lead to either tumor-suppressive or oncogenic effects, depending on the cancer type.^405,407^ Alterations in CDK12 are prevalent in various malignancies, including breast, colorectal, hepatocellular, ovarian, prostate, melanoma, and gastric cancers.^405,407^ CDK12 involvement also extends to esophageal, bladder, pancreatic ductal carcinomas, and diffuse large B-cell lymphoma.^405,407^ Consequently, CDK12 can be evaluated not only as a potential target for anticancer therapies but also as a promising biomarker, depending on the specific pathological context.

CDK12 plays a pivotal oncogenic role in the advancement of various tumors by regulating several key pathways, including c-MYC expression, the WNT/β-catenin signaling pathway, the ERBB–PI3K–AKT signaling cascade, MAPK signaling, and the noncanonical NF-κB pathway.^408^ Conversely, compromised CDK12 functionality is positively associated with the initiation and progression of breast and ovarian cancers, where it functions as a tumor suppressor.^409^ CDK12 loss of function results in decreased expression of DDR genes, leading to an impaired DNA repair signaling pathway and increased genomic instability, a hallmark of cancer progression.^220^

CDK12 is among the 10 most recurrently mutated genes in high-grade serous ovarian carcinoma (HGSOC), the ovarian cancer subtype with the highest mortality rate.^410^ CDK12 inactivation correlates with a genomic instability pattern characterized by tandem duplications up to 10 Mb along the HGSOC genomes, affecting 10% of cases.^409^ Notably, somatic mutations in CDK12 primarily clustering in the kinase domain, were detected in 3% of 316 analyzed HGSOC cases.^410^ Chemical inhibition of CDK12 in EOC in vitro models impairs DNA damage repair gene expression, leading to hypersensitivity to DNA-damaging agents and PARP1/2 inhibitors.^411^ Furthermore, CDK12 deficiency sensitizes ovarian cancer cells and patient-derived organoids to DNA-crosslinking agents such as cisplatin, EGFR blockers like lapatinib, and mTORC1 inhibitors like everolimus.^412^

In prostate cancer, CDK12 plays a dual role as both a tumor suppressor and an oncogene. While CDK12 loss occurs in a minority (3–7%) of metastatic prostate cancer cases, it correlates with accelerated metastatic spread and heightened genome instability, characterized by increased focal tandem duplications in cancer-related genes.^409^ CDK12 has also been identified as a critical oncogenic protein for prostate cancer cell survival. Chemical inhibition of CDK12 with THZ531 leads to decreased cancer progression by downregulating AR signaling and suppressing super-enhancer-associated oncogene expression.^413^ Moreover, CDK12 loss mediates genome instability in prostate cancer and may lead to higher neoantigen levels and T-cell infiltration, suggesting it potential suitability as biomarker for immunotherapy.^409^

CDK12 alterations have also been marked in breast cancer. Subtype-specific analysis revealed that 53.9% of HER2-positive samples display high CDK12 levels, while Triple Negative (TN) samples showed CDK12 absence in 17% of cases.^414^ CDK12 can be considered a potential biomarker for patient stratification and treatment since its expression significantly correlates with reduced patient survival in these specific subtypes.^414^ Elevated CDK12 expression in HER2+ breast cancer is dependent on concurrent amplification of the chr17q12 region, predominantly affecting CDK12 and HER2 genes, correlating with disease recurrence and diminished survival rates.^414^ Conversely, ERBB2 amplification within the 17q12-q21 amplicons can disrupt the CDK12 gene in 13% of HER2+ breast cancers, resulting in its loss of function and increased sensitivity to PARP1/2 inhibitors.^415^ CDK12’s oncogenic function in HER2-amplified breast cancer cells has been associated with augmented invasiveness, mediated by CDK12-dependent alternative splicing of last exons (ALE).^225^ Within HER2-enriched cancers, oncogenic CDK12 promotes tumor initiation, cancer stem cell self-renewal, and resistance to trastuzumab therapy by activating ERBB-PI3K-AKT or WNT signaling pathways.^416^ Inhibition of CDK12 enhances the anticancer effects of trastuzumab in HER2+ breast cancers, while CDK12 overexpression has been associated to resistance to trastuzumab in HER2+ cancer. Moreover, overexpression of CDK12 promotes breast tumorigenesis by hyperactivating the serine-glycine-one-carbon network.^417^ Consequently, in vivo treatment of CDK12-overexpressing breast tumors with methotrexate chemotherapy yielded positive responses, underscoring the broader implications of CDK12 not only in DNA damage response mechanisms but also in metabolism.^417^

Triple-negative breast cancer (TNBC) is an aggressive and lethal subtype that lacks targeted therapies and relies on chemotherapy as a mainstay treatment. In TNBC CDK12 acts as a tumor suppressor and targeting CDK12 with SR-4835, a potent, non-covalent, and selective ATP-competitor CDK12/13 inhibitor, reduced cancer growth both in single and combined treatment with cisplatin or PARPi in vitro and in vivo models.^418,419^ Furthermore, CDK12/13 targeted inhibition with THZ531, a covalent and allosteric CDK12/13 inhibitor, sensitizes TNBC cells in vitro to the EGFR inhibitors gefitinib and lapatinib, an effect dependent on the cooperative action of EGFR and CDK12/13 in the 4E-BP1-regulated translation of key driver oncogenes, such as MYC.^420^ CDK12’s influence in promoting cancer progression is particularly linked to oncogenic MYC-dependent cancers, as MYC translation is dependent on CDK12.^348^ The CDK12 inhibitor THZ1 effectively suppresses MYC expression and impedes tumor progression.^348^ Additionally, CDK12 inhibition with THZ1 or THZ531 is synthetic lethal with PARP/CHK1 inhibitors in Ewing’s sarcoma, dependent on the EWS–FLI1 fusion oncoprotein.^421^

In conclusion, CDK12 plays diverse roles that highlight its potential as a prognostic marker and therapeutic target for a range of human cancer types. Developing a deeper understanding of its molecular mechanisms might lead to better cancer treatment strategies and improved patient outcomes.

**CDK13**. The specific contribution of the CDK12 paralog gene CDK13 in cancer remains inadequately understood. CDK13’s involvement in cancer proliferation has been explored in prostate, ovarian, colorectal, melanoma, and breast cancers, exhibiting both oncogenic and tumor-suppressive properties depending on the context.^227,422–424^

Analysis of the TCGA database reveals that CDK13 is among the most frequently altered genes in serous ovarian cancer (2.5% of the cases), where mutations in CDK12 and CDK13 are mutually exclusive.^412^ In general, CDK12 alterations are primarily characterized by inactivation, while CDK13 is more commonly amplified than deleted.^412^ Dual inhibition of CDK12/13 in ovarian cancer induces an antiproliferative effect, eliciting genome instability.^423^ Similar studies have demonstrated that dual inhibition of CDK12/13 suppresses oncogenic growth in leukemia and triple-negative breast cancer, where it synergizes with DNA-damaging agents and PARP inhibitors.^425,426^

In human prostate cancer, CDK13 is overexpressed, driving cancer cell proliferation, by the upregulation of the transcription factor E2F5.^422^ Recent findings have also linked the proliferation of prostate cancer cells and their resistance to therapy with increased fatty acid synthesis and lipid accumulation, processes in which CDK13 is implicated.^427^ Elevated levels of CDK13 lead to upregulation of acetyl-CoA carboxylase (ACC1) expression by recruiting the RNA-methyltransferase NSUN5, which stabilizes ACC1 mRNA through m5C modification.^427^ Increased ACC1 expression promotes lipid synthesis and accumulation, thereby accelerating prostate cancer progression.^427^

Additionally, the involvement of CDK12/13 in MYC-driven oncogenesis has been demonstrated in medulloblastoma.^428^ Compared to tumors with low MYC expression, medulloblastomas with high MYC expression are more sensitive to CDK12/13 inhibition, which synergizes with DNA-damaging agents like cisplatin and olaparib.^428^ These results highlight the potential therapeutic advantages of targeting CDK13 in cancer therapy, especially in MYC-driven cancers.

In contrast to its oncogenic properties observed in various cancers, CDK13 has been identified as a tumor suppressor in melanoma. Analysis of the TCGA database revealed that CDK13 is heterozygously deleteriously mutated in 4.6% of melanoma samples, with a notable enrichment in the ATP-binding site of the kinase domain.^227^ Patient-derived CDK13 mutations act as dominant-negative on wild-type CDK13, thereby accelerating oncogenesis in both in vitro and in vivo models of melanoma. CDK13 loss of function in melanoma has been linked to impaired RNA surveillance. Prematurely terminated RNAs (ptRNAs) accumulate in CDK13-mutated melanoma due to decreased CDK13-dependent S475 phosphorylation of ZC3H14, a component of the PAXT complex involved in normal nuclear ptRNA degradation.^227^ This accumulation results in the translation of truncated proteins, contributing to the oncogenic phenotype.^227^

In conclusion, while the exact role of CDK13 in oncogenesis is still being elucidated, it shows promise as a potential target for treatment across a variety of malignancies. Understanding the diverse functions of CDK13 could lead to more effective cancer therapies and improved patient outcomes.

**CDK20**. CDK20 (CCRK) is possibly implicated in cell cycle regulation and progression across various cancer types, including glioblastoma, liver cancer, ovarian cancer, and colorectal cancer.^232,429^ CDK20 is commonly overexpressed in cancer, correlating with poor prognosis and tumor staging, although mutations in its gene are infrequent.^232,429^ The exact mechanism underlying CDK20 upregulation and its oncogenic properties varies across different tissue types.^232,429^

In glioblastoma cells CDK20 plays a role in cancer growth as a CDK-activating kinase (CAK), phosphorylating CDK2 and regulating the G1/S cell cycle transition.^232^ CDK20 knockdown by RNA interference (RNAi) reduces phosphorylated CDK2 (pCDK2) levels, causing G1 arrest and suppression of proliferation.^430^ Similarly, in colorectal cancer models, CDK20 downregulation inhibits cancer cell proliferation by inducing G0-G1 cell cycle arrest. Additionally, high CDK20 levels contribute to ovarian cancer progression by upregulating cyclin D1 expression, thereby promoting cell proliferation.^431^ In lung cancer tissues, CDK20 overexpression enhances the KEAP1-NRF2 pathway, promoting tumor progression and radio-chemoresistance by mitigating oxidative stress and exerting a cytoprotective role.^432^

In hepatocellular carcinoma (HCC), the androgen receptor (AR) acts as a positive regulator of the CDK20 promoter. Overexpressed CDK20 promotes HCC progression by activating the Wnt/β-catenin/TCF/EZH2 signaling pathway, creating a positive transcriptional feedback loop that enhances AR signaling.^433^ Hepatic activation of EZH2, dependent on CDK20, stimulates IL-6 production and the accumulation of immunosuppressive T cells.^434^ Hence, inhibition of CDK20 may disrupt this immunosuppressive tumor microenvironment and enhance the therapeutic efficacy of immune checkpoint blockade. Recently, CDK20 has been implicated in the activation of the mTORC1/4E-BP1/S6K/SREBP1 cascades through GSK3β phosphorylation, sustaining a proinflammatory signaling pathway that promotes non-alcoholic steatohepatitis (NASH)-related hepatocarcinogenesis.^435^ In HCC patients with hepatitis B virus (HBV) infection, higher CDK20 levels are observed, which are dependent on the HBx viral protein.^436^ The involvement of CDK20 in liver carcinogenesis and its oncogenic activity in multiple other cancer types, render CDK20 a promising therapeutic target. However, identifying its interactors and upstream regulators is critical toward this possible achievement.

##### Roles of atypical CDKs in cancer

According to the mostly unexplored role of Atypical CDKs in eucaryotic cells physiology also the notion regarding their possible involvement in cancer onset and progression is still mostly understudied. Also in cancer, the majority of available notions are related to the roles proposed for CDK5. Here we will summarize the most relevant information for each atypical CDK in cancer onset and progression.

**CDK5**. CDK5 can function as either an oncogene or a tumor suppressor, depending on the type of cancer. As an oncogene, CDK5 often involves the phosphorylation of p21 at S130, leading to its degradation via the proteasome. This mechanism, particularly pronounced during the S-phase of the cell cycle, activates CDK2, enhancing its interaction with DNA polymerase δ.^437^ An oncogenic role for CDK5 has been observed in breast, lung and pancreatic cancer, while in gastric cancer it might act as tumor suppressor.

In breast cancer, CDK5 is often overexpressed and associated with poor prognosis. It mediates the epithelial-to-mesenchymal transition induced by TGF-β1 by regulating the phosphorylation of FAK at S732.^438^ Loss of CDK5 in breast cancer cells leads to mitochondrial depolarization, increased mitochondrial ROS, and fragmentation. These changes increase intracellular Ca2+ concentration, calcineurin activity, provoking mitochondria-mediated apoptosis.^439^

In lung cancer, high CDK5 expression predicts poor prognosis. Inhibition of CDK5 in the lung cancer cells suppresses cell proliferation and migration and reduces tumor growth and invasion.^440^ These effects could be due to the ability of CDK5 to affect cytoskeletal remodeling, leading to loss of cell polarity and impacts on proliferation, migration and vasculogenic mimicry formation.^441^

CDK5 or its partners p35 and p39 are amplified in 67% of human pancreatic ductal adenocarcinoma (PDAC) and highly expressed in 90% of PDACs. Inhibition of CDK5 reduces migration and invasion of PDAC cells. Of note the K-Ras G12D mutants, frequently present in PDAC, increases the expression of p25, thus enhancing CDK5 activity.^442^

In gastric cancer, CDK5 is downregulated compared to healthy tissue, with its reduction correlating with increased cancer aggressiveness, lymph node metastasis, and lower five-year survival rates. CDK5 interacts with PP2A, another protein downregulated in gastric cancer, and correlates with worse overall survival. In gastric cancer CDK5 promotes apoptosis and sensitizes cells and mice to oxaliplatin through the stabilization of DP1 and activation of the E2F1 pathway.^443^

**The PFTAIREs subfamily**

Few notions are available on the possible role of CDK14 and CDK15 in cancer progression showing some degree of variability among the different tumors and or the different studies resulting in a still unclear picture of their possible involvement in tumor progression. Below are reported the most compelling evidences.

**CDK14** has been implicated in pathobiology of many cancers, including liver, ovarian, breast, gastric, pancreatic, esophageal, glioblastoma and osteosarcoma,^444–454^ although most of these studies derived from the analyses of cell lines rather than strong evidences from primary tumor samples.

In invasive hepatocellular carcinoma (HCC) and pancreatic cancer, CDK14 overexpression promotes invasiveness and migration and activating mutations of CDK14 have been identified in HPV + HCC patients.^455^

In esophageal squamous cell carcinoma literature data indicated that CDK14 activation by the CAK complex correlated with platinum resistance and poor overall survival, prompting cell proliferation through RB upregulation.^448^

Negative regulation of CDK14 results from the activity of several miR (i.e. miR-26b, miR-29b-3p, miR-1825) or lncRNA (i.e. lncRNA MSC-AS1, lncRNA OIP5-AS1), which control its expression and inhibit cell proliferation.^447,449,456,457^

**CDK15** Also CDK15 has been associated with cancer progression, although its effects appear to be context dependent. In breast and colon cancer cells CDK15 can mediate resistance to TRAIL-induced apoptosis leading to cell survival.^458^ Yet other analyses, in breast tumor specimens indicated that CDK15 protein level is down-regulated in breast cancer and that CDK15 overexpression negatively modulates cell motility, repressing their migratory and invasive capabilities, leaving unclear its role(s) in this type of tumors.^459^

Epstein-Barr virus (EBV) integration can decrease the expression of CDK15 gene in nasopharyngeal carcinomas (NPCs), implicating that downregulation of CDK15 may contribute to tumor development. Lower expression of CDK15 correlated with dysregulated NF-κB pathway activity in the EBV-integrated NPC tumors.^460^

Finally, CDK15 was documented to be highly expressed in human colorectal cancer (CRC) and negatively correlated with patient progression free and overall survival. CDK15 reduction inhibited cell proliferation, anchorage-independent growth of CRC cells and tumor progression in patient-derived xenograft (PDX) model, through the regulation of β-catenin and MEK/ERK signaling cascades to drive oncogenic pathway of CRC cells, both in vivo and in vitro.^461^

**The PCTAIRE subfamily**

Recent research on PCTAIRE proteins, even if it is in its infancy, started investigating the role(s) of PCTAIREs’ in cancer biology. Here we report the most convincing evidences and refer to a recent more focused review for additional information retrieved also from the depth study of available publicly available databases.^273^

**CDK16** is reported to be widely overexpressed in several cancer types, including lung, breast, prostate, melanoma and liver cancers.^462^ Higher CDK16 expression levels were reported in lung adenocarcinoma (LUAD) and lung squamous cell carcinoma (LUSC) respect to matched normal tissues.^463^ Higher CDK16 levels correlated with poor clinical outcomes, significantly associating with low overall survival (OS). Based on Oncomine database, several studies reported that CDK16 is one of the most upregulated genes in breast cancer, compared to normal counterpart. In particular, CDK16 was found to be highly expressed in TNBC subtype and its high expression predicts low patients’ survival, correlating with metastasis and recurrence risks. Similar observations were reported also in other cancer types, including endometrial carcinoma, serous epithelial ovarian cancer, cervical cancer and colorectal cancer.^273^

CDK16 could phosphorylate TP53 at Ser315, which is trapped in the cytoplasm inhibiting its transcriptional activity, promoting cell proliferation, survival and radio-resistance.^464^ Moreover, the phosphorylation of Protein Regulator of Cytokinesis (PRC1) at Thr481 by CDK16 prevents its nuclear localization, and promotes cell proliferation.^465^ In accordance, CDK16 knockdown suppressed or reduced cell growth in several cancer types, including, among the others, breast cancer, hepatocellular carcinoma, renal cancer, and melanoma.^273,462^

CDK16 seems to play a role in cell survival by regulating apoptosis and/or autophagy.^280^ These double functions might be of particular importance in cancer biology, where autophagy could act as both as tumor-promoting or tumor-suppressive factor.^466^ Moreover, CDK16 regulates the extrinsic apoptosis pathway in prostate and breast cancer cell lines, through the stabilization of RIPK1, preventing caspase-8 cleavage making cancer cells resistant to TRAIL-induced apoptosis.^281^ These observations support the hypothesis that CDK16 can contribute in the balance between autophagy and apoptosis thereby driving cancer progression.^280,281,466^

**CDK17** has been reported to be overexpressed in pre-menopausal breast cancers patients, with gradual stage-dependent increase^273^ and a recent pan-cancer multi-omic analysis, indicates that CDK17 is generally overexpressed in malignant tumors where it seems to be associated with induction of epithelial-mesenchymal transition (EMT), estrogen receptor pathways, inhibition of apoptosis and DNA damage response.^273^ In an unbiased loss of function shRNA screening, CDK17 emerged as possibly involved in the response to platinum in epithelial ovarian cancer cells, although experimental studies are required to validate this hypothesis.^66^ However, CDK17 role(s) may differ in other cancer types and further research is needed to fully understand its potential roles in cancer progression.

**CDK18**. CDK18 has been reported to be overexpressed in gastric cancer, where it appears to promote cancer cell proliferation and reduce T-cell tumor infiltration, pituitary adenomas and breast cancer. In cutaneous T-cell lymphoma studies silencing of CDK18 inhibited the growth of T-cell lymphoma cells lines.^273^

CDK18 has been implicated in maintaining genome stability and DNA damage response. Depletion of CDK18, in colorectal cancer cells, increased endogenous DNA damage and chromosomal abnormalities (stalled replication forks), in response to replication stress mainly due to the activation ATR signaling pathway.^467^ Mechanistically, CDK18 interacts with RAD9, and RAD17, retaining them in the chromatin, a process involved in the regulation of replication stress signaling. Consistently, CDK18 allows ATR-mediated Homology-Directed Repair (HDR) activation in glioblastoma, making cells refractory to PARP inhibitors.^289^

CDK18 is also an important regulator of DNA replication stress signaling also in breast cancer where elevated protein levels of CDK18 were associated with increased sensitivity to replication stress-inducing chemotherapeutic agents. High CDK18 protein expression was linked to the basal subtype of breast cancer and improved patient survival in estrogen receptor (ER)-negative breast cancers, treated with chemotherapy. On the contrary, breast cancers with increased CDK18 mRNA was associated with poor response to chemotherapeutic agents, inducing DNA replication stress, suggesting that CDK18 mRNA and protein levels are differently regulated with hypothetically distinct predictions on patients prognosis.^468^

#### Roles of CDKs in Neurodegenerative diseases

**Fig. 6:**
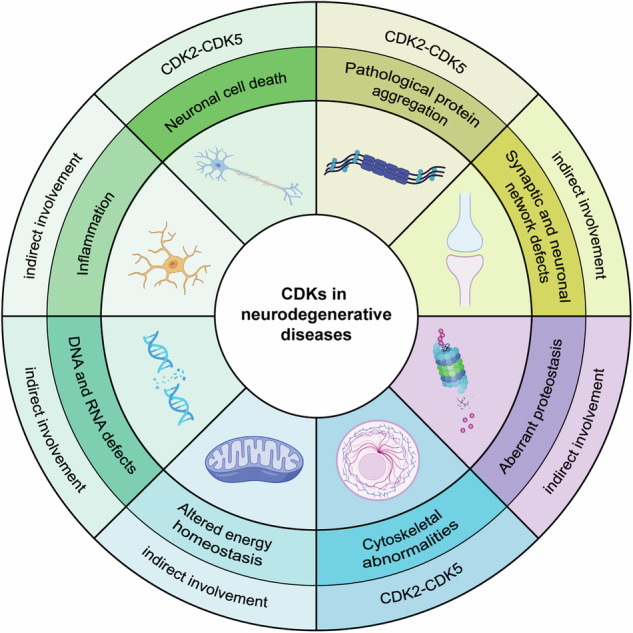
**The Role of CDKs in neurodegenerative diseases**. Involvement of various CDKs in neurodegeneration, categorized by the different stages that lead to the onset and progression of neurodegenerative diseases. The figure specifies whether the role of each CDK is direct or indirect. CDK2 and CDK5 are the only CDKs directly involved in neurodegenerative diseases by regulating neuronal cell death through alteration of ROS species and accumulation of aggregated proteins,^240,470,472,474–476,716–718^ being involved in the pathological aggregation of proteins through the regulation of tau phosphorylation^240,471–473,717,718^ and participating in the appearance of cytoskeletal abnormalities.^240,717,718^ Many other CDKs are indirectly involved in upstream processes, which subsequently contribute to the progression of neurodegenerative diseases. This distinction helps in understanding the specific and broader impacts of CDKs on the development and advancement of neurodegenerative pathologies.^240,717–730^ Created with BioRender.com

Neurodegenerative diseases represent a heterogeneous group of pathologies that leads to progressive neuronal loss in the central nervous system and or peripheral nervous system.^469^ Since many CDKs are involved in neurogenesis and neural development, it’s not surprising that they play their part also in neurodegeneration. (Fig. 6).

**CDK5** is probably the most studied CDK in neurodegenerative pathologies since its involvement in neural development. Bajaj and colleagues studied the localization of CDK5 in different familial cases of amyotrophic lateral sclerosis (ALS), observing accumulation of CDK5 in perikaryon (the cell body, non-process portion of a neuron, containing the cell nucleus), of degenerating neurons and co-localization with lipofuscin, which is associated to oxidative stress as part of the ALS pathogenesis.^470^ Furthermore, a mutant murine model expressing the superoxide variant SOD1(G37R) linked to ALS was used to investigate the role of CDK4 and CDK5 in this pathology. In comparison to wild-type animals, the mutant mice show upregulation of CDK5, and this increased activity is associated with increased phosphorylation of tau and neurofilament proteins. In these animals, neurofilament proteins show inclusion in the perikaryon, where they might act as sponge for CDK5 activity possibly ameliorating ALS symptoms.^471^

In addition, this murine model display upregulation and increased nuclear localization of **CDK4** in the motor neurons. These abnormalities are also accompanied by increased cyclin D levels (co-activator of CDK4) and increased RB phosphorylation at CDK phosphorylation sites. Interestingly, phosphorylated RB immunoprecipitate only with CDK4, not CDK5, pointing to a key role for CDK4 in the phosphorylation of RB in this context. Moreover, the overexpression of the human neurofilament H, which act as sponge to CDK5, reduced nuclear levels of CDK4 and the phosphorylation of RB.^472^

CDK5 and especially its partner p25 play a role in the pathogenesis of Alzheimer’s disease. The calpain-mediated cleavage of p35 to p25 leads to its accumulation in the brain of patients, thus leading to constitutive activation of CDK5. This over-activation causes the hyperphosphorylation of tau protein, reducing its ability to associate with microtubules and probably correlating with cytoskeletal abnormalities and death of neurons. Takahashi and colleagues investigated the localization of CDK5 and its co-factor p67 in the brain of 12 Alzheimer type dementia (ATD) patients, specifically in the hippocampus and temporal lobes.^473^ Both proteins are present in the brain of ATD patients, while they are almost absent in the controls. The co-localization of the two molecules is present in some pyramidal neurons, suggesting their involvement in the formation of neurofibrillary tangles. On the contrary, where only CDK5 is present, probably other co-factors are responsible for its activation.^473^ CDK5 immunoreactivity was present also in some astrocytes near the neurons interested by neurofibrillary tangles formation, suggesting a relationship between neurofibrillary tangles and reactive astrocytes. Investigation of Alzheimer’s disease unveiled also a new interaction between CDK5 and the p38 pathway. Both proteins are upregulated in Alzheimer patient, and CDK5 deregulation increases the amount of ROS in neural cells and cortical neurons, thus activating p38 pathway. Furthermore, p38 activation leads to upregulation of c-Jun, which is higher in Alzheimer patients, contributing to neurodegeneration.^474^

Besides ALS and Alzheimer’s disease, CDK5 might be involved also in the pathogenesis of Parkinson. In fact, CDK5 expression and activity increase after administration of a toxin able to damage the nigrostriatal dopaminergic pathway. The use of CDKs inhibitor and the overexpression of a dominant-negative CDK5 attenuates the loss of dopaminergic neurons, thus supporting the possible involvement of CDK5 in this pathology.^475^ CDK5 plays an important role in the progression of cognitive defects in diabetes. The expression of this kinase is upregulated in diabetic mice of 16 weeks of age. Moreover, in vitro experiments demonstrated that glucose administration increases CDK5 activity in a time-dependent manner due a p25-dependent aberrant activation. Inhibition of CDK5 reduced neuronal apoptosis as well as the aberrant phosphorylation of MAPK, p38 and c-Jun and more interestingly alleviated the cognitive defects in mice.^476^

Centrosome duplication has been implicated in the pathology of Parkinson’s disease, and it is initiated by the cyclin E/**CDK2** complex.^477^ In addition, CDK2 has been involved in the pathogenesis of Alzheimer’s disease due to its ability to phosphorylate tau protein.^478^

Both **CDK10** and **CDK11** are overexpressed in the encephalic tissue samples of Alzheimer’s patients, in comparison with controls especially in the cerebellum. A deeper investigation showed that CDK11 deposition is increased in the cytoplasm of Alzheimer’s processes, while it is more nuclear in controls. Additionally, they proved that the overexpression amyloid precursor protein increase CDK11 levels, promoting the cell-cycle re-entry in Alzheimer’s neurons.^479,480^

**CDK17** and **CDK18**, although expressed at high levels in differentiate neurons, are poorly investigated in neural development and neurodegeneration. Their expression is increased in neurons treated with Aβ amyloid, in samples from mild cognitive impaired and Alzheimer’s patients, possibly mediating neurodegeneration.^481^ **CDK19** involvement in neurodegeneration is limited to few case-reports. The first investigated de novo CDK19 variants in three unrelated individuals who presented hypotonia, global development delay, epileptic encephalopathy, and dysmorphic features.^482^

Overall, these data support the primary role of CDK5 in neurodegenerative diseases. Whether this is due to the lack of information about the other CDKs or to the fact that during the evolution CDK5 has become to most relevant CDK in neuronal tissue is something that should be addressed in the future.

#### Roles of CDKs in Cardiovascular diseases

Cardiovascular diseases represent the leading cause of death globally, and in particular in developed nations. Most of them result from complications associated with atherosclerosis, a chronic and progressive inflammatory condition in which vascular smooth muscle cells (VSMCs), endothelial cells, and inflammatory cells proliferate in uncontrolled way, resulting in vasculature blockage, followed possibly by myocardial infarction and stroke. Tissue remodeling within the cardiovascular system is a finely tuned balance between molecules that promote and inhibit cellular proliferation, so recent studies have revealed the important role of the CDKs also in this context. Cyclins and CDKs expression is strongly repressed in cardiomyocytes after birth to arrest their proliferation whereas the levels of negative cell-cycle regulators, such as p21, p27 and RB are increased.^483^

After myocardial infarction, the re-activation of cardiomyocytes proliferative potential could be exploited as a strategy to achieve the regeneration of damaged tissue. Overexpression of a combination of four cell-cycle regulators including the **CDK1**-CCNB and **CDK4**-CCND complexes, effectively promoted cardiomyocytes proliferation and subsequent cell survival following acute or subacute myocardial infarction, both in vitro and in vivo.^484^ Interestingly, the sustained improvement in cardiac function was observed also four months after treatment.^485^ CDK1 is potentially involved in the repair mechanisms of infarcted hearts.^484^ The cyclin D1/CDK4 complex is crucial for triggering cell cycle reentry in numerous cardiomyocytes within the adult heart. An increased amount of cyclin D1/CDK4 complexes has been associated with cardiac hypertrophy.^486^ Moreover, following cardiac injury, targeting the cell cycle inhibitor p16^INK4a^ leads to CDK4/6-mediated proliferation, resulting in myocardial regeneration and repair both in vitro and in vivo.^487^ Furthermore, CDK4 has been identified as a direct transcriptional target of GATA4, a gene known to regulate cardiomyocyte differentiation and proliferation.^488^

Similar to CDK4, numerous studies have reported a role for **CDK6** in activating cardiomyocyte proliferation and inducing hypertrophy. One potential regulatory mechanism for CDK6 involves its association with microRNAs (miRNAs). Specifically, miR-1 expression is significantly reduced in hypertrophic myocardium and hypertrophic cardiomyocytes, while CDK6 protein levels are elevated. These findings suggest that miR-1 suppresses CDK6 expression, leading to the dephosphorylation of RB and resulting in the attenuation of the hypertrophic phenotype in cardiomyocytes.^489,490^

**CDK7** and **CDK9** like most cell cycle regulators, play crucial roles in catalyzing hypertrophic responses during the embryonic phases of cardiac development. However, their enzymatic activity significantly declines in adulthood. During chronic cardiac hypertrophy this decline is reversed, leading to elevated levels of both CDKs 7 and 9.^491^ Further studies have demonstrated that a dominant-negative form of CDK9 effectively inhibits cardiac hypertrophy, suggesting its potential utility as a therapeutic tool for managing this condition and addressing long-term heart diseases.^491^ Additionally, evidence shows that miR-1 can reduce the expression of CDK9 and other growth-related target genes, including CDK6.^492^ Reports on the mechanism of action of CDK7 in cardiovascular diseases are limited. However, simultaneous inhibition of CDK7, CDK12, and CDK13 has been found necessary for potent and consistent antihypertrophic responses, suggesting that CDK7 could also play a pivotal role in cardiac hypertrophy development.^493^

Regarding the **CDK13** gene, several de novo missense variants have been identified in individuals exhibiting cardiac anomalies. Congenital heart defects, facial dysmorphism, and intellectual developmental disorder (CHDFIDD) is a newly described syndrome caused by these de novo variants in CDK13.^494^ Among the affected individuals, 81% displayed some form of cardiac anomaly, with 44% having additional structural cardiac issues, ranging from left pulmonary artery defects to bicuspid aortic valves.^494^ In a large cohort of 610 patients with congenital cardiac diseases, seven patients with de novo missense mutations in the CDK13 gene were identified, suggesting an involvement of CDK13 in these conditions.^495^

Whole-exome or whole-genome sequencing analysis has also identified different mutations altering the kinase activity of **CDK8**, specifically within the ATP-binding pocket. Individuals affected by distinct missense CDK8 mutations exhibit overlapping phenotypes, including congenital heart disease or dilated cardiomyopathy (DCM).^496^ CDK8, as part of the kinase submodule of the Mediator complex (MED12, MED13, CDK8, and cyclin C), plays a role in maintaining normal cardiac function and is involved in cardiovascular diseases.^497^

Limited information is available concerning the involvement of CDK20 in human diseases. Nonetheless, a distinct CDK20 variant has been identified within cardiomyocytes, displaying exclusive localization in cardiac tissue.^498^ A correlation has been established between cardiac CDK20 and heart diseases, with overexpression of cardiac CDK20 demonstrating a cytoprotective effect on cardiomyocytes, conferring resistance against cell death and mitigating stress-induced heart failure.^499^

The use of CDK inhibitors in cardiovascular diseases is limited, with few studies focused on animal models of neointimal thickening after balloon angioplasty, a condition that contributes to restenosis. Both synthetic CDK inhibitors, such as flavopiridol, and antisense oligodeoxynucleotide strategies against CDK1/2 have shown promising results in reducing neointima formation after intervention.^500^ However, the lack of correlation between animal studies and clinical trials has decreased the possibility of using CDK inhibitors in clinical settings. Moreover, the observation that cancer patients treated with CDK4/6 could have increased death rates due to the development of atrial fibrillation/atrial flutter or heart failure, raises some concerns for their use in cardiovascular disease and suggests that further research is needed to determine cardiovascular risks associated with the use of CDKs inhibitors in clinic.^501^

#### Role of CDKs in Inflammatory, metabolic and autoimmune conditions

##### Metabolic disorders

**Fig. 7:**
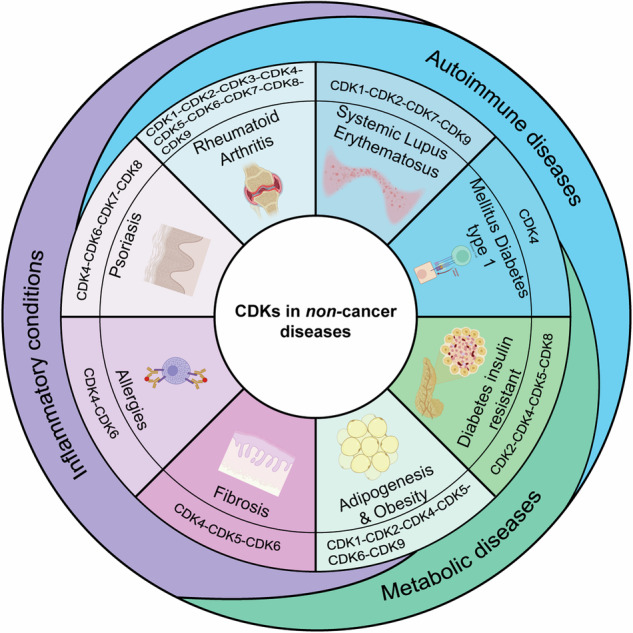
**The Role of CDKs in non-cancer diseases**. Involvement of CDKs in various non-cancer pathologies, categorized into three macro-categories: inflammatory conditions, autoimmune diseases, and metabolic diseases. While these categories provide a structured overview, there is some overlap among them, reflecting the complex interplay of CDKs in these conditions. The CDKs involved in the pathogenesis of rheumatoid arthritis mainly drive the release of metalloproteinases from fibroblast-like synoviocytes (FLSs) and the proliferation of FLSs.^532,731–740^ In the systemic lupus erythematosus the inhibition of certain CDKs ameliorates the immune components of the disease.^536,741–743^ CDK4 is the only CDK involved in the pathogenesis of mellitus diabetes type I, influencing the development and the proliferation of pancreatic β-cells.^744–746^ Instead, many CDKs play a role in the pathogenesis of type II diabetes, regulating β-cells proliferation, metabolism and apoptosis.^517,519,525,526,747,748^ Different CDKs participate in the regulation of adipogenesis and obesity, regulating the differentiation of preadipocytes in mature adipocytes, tuning the expression of adipogenic genes, consequently leading to fat accumulation.^61,506–508,749–755^ Regulation of the fibrotic process by CDKs relay on their ability to regulate fibroblasts behavior and ECM remodeling, that have been investigated in CDK4, CDK5 and CDK6.^556,557,559^ Only few reports link CDKs to the pathogenesis of allergies.^756,757^ In psoriasis CDKs, by altering the homeostasis of several pathogenic cytokines, promote inflammation and proliferation of different cells types.^548,549,758,759^ Created with BioRender.com

Accumulating evidence suggests that CDKs could be involved in metabolic regulatory mechanisms. The processes of metabolism and cell growth are closely connected and mutually supportive. Specifically, adaptive anabolic biosynthesis pathways are triggered by stimuli that support cell development and provide the energy and metabolites needed to sustain cell proliferation. This interaction is fundamental in physiological regulation, but it is also involved in the development of related alterations and dysregulations. Beyond cancer-related metabolic alterations (i.e. enhanced glycolysis and de novo fatty acid synthesis), cyclin/CDK complexes have been implicated in several metabolic disorders, mainly obesity and diabetes, which are finely interconnected.^502^ This involvement has been confirmed by genetically inactivated CDK and cyclin mouse models, which phenotypically exhibit reduced body size and metabolic abnormalities (Fig. 7).^502^

**Obesity and Adipogenesis**

Obesity is featured by abnormal fat accumulation, which in turn can lead to insulin resistant phenotype and type II diabetes. Hyperinsulinemia induces cell cycle progression and senescence in adipocytes, contributing to adipose tissue inflammation. Dysregulated CDK activity can impair insulin sensitivity in insulin-responsive tissues such as adipose tissue, liver, and skeletal muscle.^503^ In particular, CDKs are involved in the differentiation of preadipocytes into mature adipocytes, regulating the expression of adipogenic genes, thus promoting fat accumulation in adipocytes.^502^ Genetic data supports this involvement, as combined deletion of the CDK inhibitors p27 and p21 in mice induced an increase in adipocyte number and fat pad weights, leading to hypercholesterolemia, glucose intolerance and obesity.^504^ In particular, cell cycle CDKs, such as **CDK4** and CDK6, might act as a mediators of homeostatic signals, potentially orchestrating metabolic activation.^61,502^ The cyclin D/CDK4-E2F1-RB axis drives cell cycle progression, supporting the proliferative phase, crucial for preadipocyte expansion and differentiation.^61^ In non-proliferative, differentiated adipocytes, CDK4 interacts with PPARγ (peroxisome proliferator-activated receptor γ), a master regulator of adipogenesis and adipose-specific gene expression, enhancing its transactivation.^61^ Consistently, mice lacking cyclin D3 or CDK4 exhibit fewer adipocytes and less adipose tissue mass.^505^ Inhibiting CDK4, or its associated cyclin D3, in adipocytes, lowers insulin sensitivity, glucose uptake, and gene expression related to lipogenesis and insulin signaling.^61,505^ Recent investigations highlight that **CDK6** kinase activity negatively regulates the conversion of fat-storing cells into fat-burning cells by suppressing RUNX1, dissecting how adipose tissue deposition can contribute to the development of metabolic diseases.^506,507^ Mice lacking CDK6, or its kinase domain, exhibit significant increases in fat cell formation in beige adipose tissue, enhanced energy expenditure, better glucose tolerance, improved insulin sensitivity, and greater resistance to high-fat diet-induced obesity.^506,507^ CDK6 has also been found to inhibit de novo lipogenesis (DNL) in white adipose tissue.^507^ CDK6 phosphorylates AMPKα, leading to the inactivation of acetyl-CoA carboxylase, a key enzyme in DNL. Treatment of mice with a CDK6 inhibitor increased DNL in visceral white adipose tissues, recapitulating the phenotypes observed in mice with a kinase-inactive allele of Cdk6.^507^ Given the role of DNL in obesity, these findings suggest that inhibiting CDK6 kinase activity or its downstream effectors could be a valid strategy to alleviate obesity and metabolic syndromes.^506,507^ Moreover, also **CDK9** seems to be involved in lipogenesis, by phosphorylating PPARγ at S112, enhancing its transcriptional activity.^508^ Elevated CDK9 expression has been observed in obese mice, indicating that it may be a potential therapeutic target for obesity.^508^ CDK9 expression is also elevated in the livers of individuals with non-alcoholic steatohepatitis (NASH). Specifically, CDK9 phosphorylates and regulates the nuclear/cytosolic translocation of Wilms’ tumor 1-associating protein (WTAP) and methyltransferase-like 3 (METTL3), contributing to ectopic lipid accumulation and inflammation as NASH progresses. Therefore, targeting CDK9-mediated pathways could offer therapeutic options for managing NASH and its associated complications.^509,510^

As mentioned, obesity often leads to insulin resistance and Type 2 Diabetes. In vivo models of obesity induced by a high-fat diet (HFD) showed **CDK5** hyperactivation in adipocytes, driven by elevated levels of circulating cytokines, such as IL-6 and TNFα.^511^ In turn, CDK5 phosphorylates the nuclear receptor PPARγ at Thr273, promoting fibroblast conversion into fat cells.^511^ This modification alters PPARγ functionality, affecting genes involved in metabolic regulation and adipokine production, including the insulin-sensitizing hormone adiponectin, ultimately contributing to insulin resistance.^511^ Several anti-diabetic PPARγ ligands have been shown to inhibit CDK5-mediated phosphorylation of PPARγ, thereby restoring insulin sensitivity.^511,512^ Interestingly, selective CDK5 inhibition in adipose tissue enhances PPARγ phosphorylation through a compensatory ERK activation mechanism involving reduced CDK5-dependent MEK1 phosphorylation.^513^ Additionally, also the cyclin C/**CDK8** complex has been proposed to link the insulin pathway with lipid homeostasis.^514^ Insulin stimulation activates Sterol Regulatory Element-Binding Protein 1 isoform c (SREBP-1c), initiating hepatic lipogenesis.^515^ However, CDK8 phosphorylates SREBP-1c at the T402 residue, leading to its proteasome-dependent degradation and thus negatively regulating de novo lipogenesis in mouse models.^514^ Elevated SREBP-1c levels are associated with various cancers, obesity-related insulin resistance, and non-alcoholic fatty liver disease (NAFLD).^516^ These findings indicate that CDK8 dysregulation, resulting in altered SREBP-1c degradation, could disturb hepatic lipid balance, potentially leading to severe outcomes.^514^

**Type 2 Diabetes**

Type 2 diabetes (T2D) is characterized by elevated blood glucose levels due to insulin resistance and impaired functionality and apoptosis of pancreatic islet β-cells. CDKs regulate β-cell proliferation and insulin production, suggesting their involvement in T2D pathogenesis.^502^ Consistently, the loss of **CDK2** has been linked to impaired β-cell function, leading to a gradual decline in β-cell mass and culminating in diabetes.^517^ Mice with pancreatic CDK2 knockout exhibit glucose intolerance, characterized by altered insulin secretion, disrupted β-cell metabolism, and impaired mitochondrial architecture.^517^ These effects may be due to dysregulated phosphorylation of Forkhead box protein O1 (FOXO1), due to CDK2 deficiency.^517,518^ However, genetic inactivation of CDK2 in adult pancreatic tissue, rather than in embryonic deletion models, conferred a protective benefit, enhancing glucose tolerance and insulin secretion from adult β-cells.^519^ Additionally, **CDK4**, in conjunction with cyclin D1 and D2, is involved in pancreatic β-cell proliferation, differentiation and insulin sensitivity.^57,520,521^ Mice lacking CDK4 expression present smaller body sizes and develop diabetes due to insulin resistance, caused by decreased pancreatic β-cell proliferation and function.^57^ The involvement of CDK4 in β-cell proliferation and metabolic response is closely tied to insulin signaling.^522^ High levels of insulin increase CDK4 activity, leading to the phosphorylation of RB and the subsequent activation of E2F1 transcriptional activity in response to glucose in β-cells, in both cell cycle-dependent and -independent manners.^522^ The insulin-induced CDK4-RB-E2F1 axis promotes the expression of glucose-induced genes and impacts the regulation of insulin secretion in pancreatic β-cells.^522^ Furthermore, β-cell proliferation relies on the activation of CDK4 by the PI3-kinase/Akt kinase signaling pathway, induced by insulin expression.^523^ Transgenic mice with constitutively active Akt, but lacking CDK4 expression, exhibit lower β-cell proliferation, indicating that Akt promotes β-cell proliferation in a CDK4-dependent manner.^523^ On the other side, in vivo studies demonstrated that activation of cyclin D1/CDK4 complex in the liver results in enhanced GCN5 acetyltransferase activity and inhibition of hepatic glucose synthesis, independent of cell cycle progression.^524^ In the T2D mice models, cyclin D1 protein levels are dysregulated due to hyperinsulinemia and insulin resistance, contributing to an inability to fully repress gluconeogenesis, a key pathological feature of T2D.^524^

T2D is also characterized by either complete or relative insufficiency of insulin secretion. In this context, CDK5 role in glucose metabolism and diabetes has been extensively studied.^260^ **CDK5** activation positively regulates insulin promoter transcription, thereby increasing insulin production under physiological conditions.^260^ However, dysregulation of CDK5 and its activating partner, p35, has been linked to β-cell failure in high-glucose conditions.^260^ Yet, CDK5 has also been implicated in promoting β-cell survival. Interestingly, CDK5 inhibition can mitigate diabetes-induced neuronal death, potentially offering a therapeutic strategy for diabetes-related cognitive deficits.^525^

On the other hand, under physiological conditions, **CDK8** acts as a negative regulator of insulin secretion within pancreatic islets.^526^ Conditional knockout of CDK8 in β-cells enhances glucose tolerance by increasing insulin secretion, which is mediated by the phosphorylation and activation of its substrate, oxysterol-binding protein-related protein-3 (OSBPL3).^526^ CDK8 also promotes β-cell survival during metabolic oxidative stress, by suppressing the expression of neuropeptide Y (NPY) in adult differentiated β-cells, thereby preventing apoptosis.^526^ Aberrant NPY expression in CDK8-deficient β-cells leads to apoptosis and subsequent diabetes development.^526^ Additionally, CDK8 suppresses β-cell differentiation through its regulation of the TGF-β/BMP and Wnt/β-catenin signaling pathways, which are established targets of CDK8.^361,527^

Overall, CDKs seem to play a significant role in metabolic diseases, by regulating several processes, such as lipogenesis, adipocyte differentiation, glucose uptake and insulin secretion. Inhibition or modulation of some CDK activity may open new perspectives in the management of metabolic diseases, such as T2D and obesity.

##### Autoimmune diseases

Autoimmune diseases are clinical syndromes characterized by the alteration of B and/or T cells selection, regulation or death, in the absence of an ongoing infection or other identifiable cause. Role of CDKs in the development of these diseases and their potential as therapeutic targets has emerged in different clinical syndromes (Fig. 7).

**Rheumatoid arthritis**

Rheumatoid arthritis (RA) is a complex autoimmune disease with an etiology influenced by environmental, genetic, and epigenetic risk factors. The pathogenesis of RA involves multiple cell types, including B and T lymphocytes, macrophages, osteoclasts, neutrophils and, in particular, fibroblast-like synoviocytes (FLS), whose activity is crucial to perpetuate inflammation and cartilage destruction. CDKs play a crucial role in RA FLSs-mediated pathogenesis, primarily by driving metalloproteinases (MMPs) release from FLSs, as result of **CDK3,** **CDK4/6** or **CDK9** activity^139,528,529^ and secondly, by promoting FLSs proliferation through the miR-124a-**CDK2** axis.^530^ CDKs are also involved in controlling several RA-neutrophils essential functions. These cells have the ability to release neutrophil extracellular traps (NETs) and exhibit delayed apoptosis, liberating pro-inflammatory factors. Treatment with CDK4/6 inhibitors impairs NET release,^531^ while inhibitors of CDK2, CDK5 and CDK9 promote apoptosis of RA-neutrophils.^532^

So far, several mouse models have been developed to test the efficacy of CDK inhibitors in reducing RA symptoms by blocking synovial hyperplasia.^533^ Together, these findings highlight the potential of CDK inhibitors (also in combination with anti-inflammatory therapy) as therapeutic agents in RA, targeting various pathways and cell types involved in the pathogenesis of the disease.

**Systemic lupus erythematosus**

Systemic lupus erythematosus (SLE) is a complex autoimmune disease affecting various organs, where activation of interferon (IFN) genes is indicated as one of key step. It has been demonstrated that **CDK1** positively regulates the type I IFN signaling pathway in SLE, facilitating the development of severe disease and contributing to the concept that CDKs impact on IFN response may pass through STAT1 phosphorylation, as already demonstrated for **CDK8**.^372,534^ However, adverse side effects and less efficacy represent relevant limitations for the application of CDK1 inhibitors in clinic. Yet, as the interphase CDK2, CDK4 and CDK6 are not absolutely required for cell cycle progression, targeting these kinases should have fewer side effects than targeting the mitotic kinase CDK1.^535^ A pilot study using seliciclib, a CDK2, CDK7 and CDK9 inhibitor, showed a remarkable amelioration of the immune components of SLE. Mice treated with preventive or therapeutic regimens of seliciclib demonstrated a downmodulation of anti-DNA antibodies, reduced IgG levels in sera, and decreased IgG deposition within glomeruli. Additionally, treated mice exhibited a reduction in the accumulation of activated/memory T and B cells.^536^

These findings highlight the potential for CDK inhibitors as therapeutic agents in SLE, offering new avenues for treatment that may reduce the immune system overactivity and alleviate disease symptoms, while minimizing side effects.

**Type 1 Diabetes Mellitus**

Type 1 diabetes mellitus (T1DM) is a heterogeneous chronic disease characterized by insulin deficiency due to the loss of pancreatic β-cells, resulting in hyperglycemia. The pathogenesis of T1DM involves prevalently macrophages and dendritic cells, which release IL-12, essential for activating CD4+ T cells and, in turn, CD8+ T cells differentiation into cytotoxic T cells. The disruption of insulin-producing pancreatic β-cells by CD4+ and CD8+ T cells leads to the release of autoantigens, which are processed and presented to T helper cells by antigen-presenting cells.^537,538^

While **CDK4** plays a central role in murine β islet development, CDK6 expression is marginal or undetectable in mice.^56,539^ However, in human β islets, the expression and function of CDK4 and CDK6 are similar and redundant. In vitro evidence indicates that overexpression of cyclin D-CDK4/6 complex leads to uniquely robust proliferation of human β-cells^540^ and CDK4 R24C knock-in NOD mice developed diabetes and insulitis, exacerbated by NOD immune repertoire background.^539^

While various interventions to preserve β-cells and improve clinical disease management have been tested, significant gaps still exist in our understanding of T1DM. Research on CDK4/6 activity and regulation represents a promising field for developing new therapeutic strategies. Thus, understanding the precise roles and interactions of CDKs could lead to significant advancements in preserving β-cell function and better managing the autoimmune aspects of this disease.

##### Inflammation

Inflammation is a complex biological response of body tissues to harmful stimuli, such as pathogens, damaged cells, or irritants. This response triggers the immune system to remove the injurious stimuli and initiate healing. During the spontaneous resolution of inflammation, neutrophils undergo apoptosis, leading to the cessation of their secretory activity, enabling their recognition and removal by macrophages. However, under certain circumstances, the inflammatory process can become excessive or chronic, causing tissue damage and contributing to various diseases, including cancer (Fig. 7). CDKs contribute to inflammation through various mechanisms, in different cells involved and during different steps of the process. Understanding these roles may lead to the development of novel strategies to counteract inflammatory diseases.

Much of the knowledge on the contribution of CDKs in the most common inflammatory conditions comes from the use of CDK inhibitors. Flavopiridol arrests cell cycle progression in the G1 or G2 phase by inhibiting all cell cycle CDKs, along with transcriptional CDK7 and CDK9, thereby eliciting anti-inflammatory activity, especially by the targeting of CDK9 and NFκB-dependent signaling.^541^ Since apoptosis is essential for timely clearance of inflammatory cells, flavopiridol inhibition supports an efficient resolution of inflammation. More recently, it was shown that **CDK9** inhibition, by suppressing IFN-γ and TNF-α production in both murine and human CD4+T cells, led to potent repression of the genes responsible for the pro-inflammatory signaling, in a model of anti-TNF-resistant Inflammatory Bowel Disease.^542^

Similar to flavopiridol, R-roscovitine, a pan CDK inhibitor, enhances the resolution of neutrophil-dependent inflammation.^543^ Using three different disease models in mice, i.e. acute pleurisy, bleomycin-induced lung injury, and passively induced arthritis, R-roscovitine enhanced the resolution of established inflammation in vivo. Although neutrophils are terminally differentiated and, therefore, CDK inhibitors would be predicted to have no effect, the authors demonstrated that R-roscovitine acted by reducing the concentrations of the anti-apoptotic protein Mcl-1, eventually promoting the apoptosis of neutrophilic inflammatory cells.^543^

The treatment of human monocyte-derived macrophages with R-roscovitine or with specific CDK9i, at concentrations that induce neutrophil apoptosis, had no significant effect on macrophages apoptosis and viability but significantly downregulated the expression and release of cytokines, as well as markers of pro-inflammatory macrophage polarization.^544^

Altogether, given their role in regulating inflammation, CDKs have emerged as potential therapeutic targets for inflammatory diseases and CDK inhibitors are being explored for their anti-inflammatory properties in preclinical and clinical studies. These inhibitors may offer a way to selectively modulate inflammatory responses without broad immunosuppression. In the next paragraphs, a description of CDKs involvement in some of the most frequent diseases characterized by chronic, excessive or inappropriate inflammatory response, will be provided.

##### Psoriasis

Psoriasis is a chronic immune-mediated inflammatory skin disorder, characterized by hyperproliferation of keratinocytes. CDKs play significant roles in the pathogenesis of psoriasis, by promoting proliferation and inflammation in different cell types, modulating the immune response and the release of pathogenetic cytokines, such as TNF-α, IL-17, IL-22 and IL-23.^545,546^

Dysregulated CDK activity leads to accelerated cell cycle progression and hyperproliferation of keratinocytes, contributing to the thickened, scaly plaques characteristic of the disease. The abnormal expression and activation of CDKs in psoriasis results in disrupted cell cycle checkpoints, allowing keratinocytes to bypass normal regulatory mechanisms. This unchecked cell division is a hallmark of the psoriatic epidermis, in which the expression of p16 is either decreased and can be induced by phototherapy, or, when psoriatic cells undergo a senescence switch, elevated.^545^

In a cohort of 24 psoriatic patients, it was reported that CDK2–cyclin E expression and activity were significantly increased in psoriatic epidermis compared with adjacent skin. In contrast, CDK4 was activity was inhibited, in accord with an increased expression of structural CDK inhibitors, mainly p21 and p16^INK4a^.^547^

CDKs also play a role in modulating the inflammatory response in psoriasis, by regulating immune cell proliferation and functions, affecting T cell activation and cytokine production, particularly in Th17 cells, overactive in psoriasis. Once activated, these cells produce high levels of IL-17 and IL-22, which promote keratinocyte proliferation and inflammation, driving the inflammatory cascade in psoriasis. CDKs also influence the activity of transcription factors such as NF-κB and STAT3, which are involved in the expression of pro-inflammatory cytokines and chemokines.^546^ Using different psoriasis-like mouse models, Muller and collaborators identified a novel proinflammatory signaling pathway driven by **CDK4** and **CDK6** that, through phosphorylation of EZH2 in keratinocytes, triggered a methylation-induced activation of STAT3. Repurposing of CDK4/6 or methyltransferase EZH2 inhibitors may thus serve as therapeutic target for patients with psoriasis.^548^ Accordingly, topical administration of palbociclib resulted in reduced erythema, inflammation, desquamation and scaling in a mouse model of psoriasiform dermatitis.^546,548,549^

Literature offers very limited information regarding the use of registered CDK inhibitors in patients with psoriasis. However, in accord with preclinical studies, CDK inhibitors might have promising potential as therapeutic agents, by reducing both the hyperproliferative and inflammatory aspects of the disease. It is conceivable that CDK4/6 inhibitors may slow down the rapid turnover of keratinocytes, while also modulating immune cell activity. However, since it is known that psoriatic keratinocytes enter a state of senescence, the process of evaluating the benefits of CDK4/6i, potent inducers of a senescent state, might reveal to be quite complicated. For instance, a case report showed two episodes of psoriasis relapse in a breast cancer patient treated first with ribociclib, then with palbociclib. Remarkably, the psoriatic plaques resolved soon, after each of CDK4/6i was stopped. This paradoxical effect might be related to the induction of abnormal terminal differentiation and senescence.^550^

##### Allergy

Roles played by CDKs in immune and inflammatory processes, as described above, are also relevant to the pathogenesis of allergies. Since CDKs regulate the cell cycle progression of various immune cells, including T and B cells, their dysregulated activity in the presence of an allergen may lead to excessive immune cell proliferation, eventually amplifying an allergic response.

Eosinophils are terminally differentiated cells and, as such, should not require cell-cycling machinery such as CDKs. Nonetheless, like other terminally differentiated cells, including neutrophils, they have measurable expression of CDKs. CDK inhibition drove eosinophil apoptosis by the mitochondrial pathway, via suppression of Mcl-1L expression, and then enhanced phagocytic clearance of eosinophils by macrophages.^551^ Since eosinophils are major players in inflammatory allergic diseases, particularly in conditions like asthma and allergic rhinitis, any strategy that decrease eosinophil recruitment and activation or drives eosinophil apoptosis and clearance could in principle ameliorate allergic symptoms.

In summary, very little literature is available linking CDKs to the pathogenesis of allergies, although their roles in immune cell proliferation, differentiation, cytokine production, mast cell and eosinophil function, seem to support their indirect involvement in the modulation of allergic responses.

##### Fibrosis

The pathogenesis of fibrosis may be viewed as the failure of a wound healing process, leading to excessive tissue scarring and organ dysfunction. It can be triggered by various injuries, such as toxins, hypoxia, or metabolic disorders, and is often due to a dysregulated cell cycle. Fibrosis is linked to many chronic diseases, causing significant morbidity, and mortality, but no specific anti-fibrotic drugs are currently available. From a pathological point, fibrosis involves excessive deposition of extracellular matrix (ECM). Fibroblasts, the key cells in ECM production, are regulated by cyclin-dependent kinases (CDKs), which influence their activation, proliferation, and differentiation into myofibroblasts. CDKs control both cell cycle progression and the expression of ECM components like collagen and fibronectin, primarily through transcriptional regulation rather than just cell cycle control.^552^

In kidney as well as in liver, the identification of factors that are specifically important for driving fibrosis without affecting general organ homeostasis or regeneration, would be crucial. Pan-CDK inhibitors like roscovitine have shown promise in blocking aberrant CDK activity in mouse models, affecting fibroblast activation, ECM turnover, and the balance between ECM synthesis and degradation.^553^ Using various kidney injury models, CDK inhibition prevented renal fibrosis by modulating cell cycle dysregulation and reducing the secretion of profibrotic cytokines, such as CTGF and TGF-β1.^553^

Similar results were also reported for liver fibrosis, in which roscovitine repressed the transcription of a broad set of pro-inflammatory genes involved in cytokine production and immune cell proliferation and migration, and inhibited the TGF-β signaling pathway and the biological process of tissue remodeling. This anti-inflammatory effect of roscovitine was accompanied with reduced liver fibrosis, in mice.^554^

However, single or concomitant genetic loss of cyclin E1, cyclin E2, or **Cdk2** in mice did not prevent proper liver regeneration after partial hepatectomy, suggesting high functional redundancy of cell cycle proteins.^555^ But liposome-based delivery of cyclin E1- specific small interfering RNA (siRNA) reduced the proliferation and infiltration of pro-fibrotic leukocytes in the challenged liver and attenuated the overall inflammatory response after a pro-fibrotic stimulation.^555^ The use of second-generation pan-CDK inhibitor CR8 restricted the pro-fibrotic properties of hepatic stellate cells, while preserving the regeneration capacity of hepatocytes under the same conditions, overall suggesting promising therapeutic translatability.^555^

Persistent fibroblast activation and myofibroblast phenoconversion underlying multi-organ fibrosis in systemic sclerosis have been associated with high expression and activity of **CDK5** and its CDK5R1 subunit p35.^556^ The authors demonstrate that CDK5 activity stimulates collagen production and myofibroblast markers, whereas CDK5 knockdown abrogated TGF-β fibrotic responses, suggesting that selective pharmacological targeting of CDK5 might represent a successful approach to treat fibrosis.^556^

Less is known regarding the involvement of CDKs in pulmonary fibrosis and their activity might be more controversial in this anatomical district. Case report studies show the rare possibility of severe lung injury elicited by the treatment with the CDK4/6 inhibitor palbociclib during the PALOMA 3 trial, especially in the Asian population^557^ or that palbociclib enhances pulmonary fibrosis in patients undergoing thoracic radiation therapy.^558^

The mechanisms underlying lung injury related to CDK4/6 inhibitors remain largely unclear. It is possible that the altered inflammatory environment, combined with the effects of a cell cycle inhibitor like palbociclib, could induce cell senescence and trigger a “senescence-associated secretory phenotype” (SASP), which is a known promoter of pulmonary fibrosis. Accordingly, in a preclinical model of bleomycin-induced lung fibrosis in mice, treatment with palbociclib significantly reduced collagen deposition in the lungs but failed to prevent the decline in lung function and increased inflammatory cell recruitment in bronchoalveolar lavage fluid.^559^

Overall, CDKs seem to play a significant role in fibrosis by regulating various cellular processes, including fibroblast activation, ECM remodeling, inflammation, and EMT. However, more studies are needed to clarify the possible controversial effects played by CDK inhibitors in different tissue contexts.

### CDKs as therapeutic targets

**Fig. 8:**
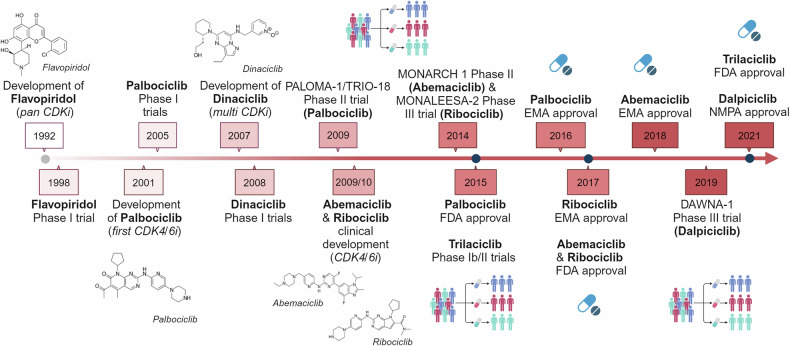
**Milestones in CDK inhibitors development: from generation to approval**. The timeline shows the discovery of the first pan-CDK inhibitor Flavopiridol, followed by the development of second generation multi-CDK inhibitors, here simplified by the sole representation of Dinaciclib. The development of the five CDK4/6 specific inhibitors approved for the use in clinics are reported (Palbociclib, Abemaciclib, Ribociclib, Trilaciclib and Dalpiciclib). For each of them, the key trials that have eventually led to their agency approval (FDA, EMA or NMPA) are indicated. More details on discovery, preclinical studies and clinical trials on these drugs can be found in the text. FDA: Food and Drug Administration; EMA: European Medicines Agency; NMPA: National Medical Products Administration. (Adapted from “Timeline (7 segments, Horizontal), by BioRender.com (2024). Retrieved from https://app.biorender.com/biorender-templates)

Since their discovery, CDKs have been attractive targets in various types of cancer.^560^ In the following section, we will discuss preclinical and clinical studies conducted with CDK inhibitors, starting with the first generation CDK inhibitors and providing evidence on why they failed to meet expectations in the clinics. Then, we will elaborate on the next-generation CDK inhibitors, their success, pitfalls in clinics and discuss which future directions may improve their clinical utility. We will then concentrate on the CDK4/6 inhibitors preclinical and clinical development that now are approved for the treatment of different types of cancers, alone or in combination with other drugs (Fig. 8).

#### Development of first generation CDK inhibitors

First-generation CDK inhibitors are characterized by their non-selective inhibition across multiple CDKs, which has led to their designation as pan-CDK inhibitors.^561^ These inhibitors are either flavonoid-based, such as flavopiridol,^562,563^ or pyrimidine-based, like roscovitine^564,565^ ATP-competitive inhibitors. Flavopiridol, for instance, inhibits multiple CDKs, including CDK1, CDK2, CDK4/6, and CDK7, with IC50 values ranging from 100 to 400 nM. In vitro studies on various solid tumor cell lines have shown that flavopiridol induces cell cycle arrest at both the G1 and G2 phases, consistent with the concomitant inhibition of CDK2, CDK4, and CDK6.^562,563,566^

Additionally, flavopiridol interferes with CDK7 and its CDK-activating kinase activity, thereby preventing the phosphorylation events necessary for the activation of other CDKs.^567^ This compound also inhibits the transcriptional CDK, CDK9,^568^ leading to impaired transcription of short half-life mRNAs, including those encoding cyclin D1 and anti-apoptosis proteins.^569,570^

The primary consequence of flavopiridol-mediated CDK inhibition is cytostatic growth arrest. However, prolonged treatment can lead to cell death and apoptosis, particularly in hematological cancers.^571–573^ In vivo studies support these findings, demonstrating significant antiproliferative activity in several human-derived xenograft models.^39,574,575^

Based on these preclinical studies, flavopiridol became the first CDK inhibitor to enter human clinical trials^576^ and has been evaluated in over 60 trials.^577^ In solid tumors, including gastric, pancreatic, prostate, renal, endometrial, and ovarian cancers, results were disappointing, typically associated with high toxicities and failing to achieve objective responses.^578–582^ Despite early clinical trials indicated potential effectiveness in hematological malignancies, follow-up trials reported frequent severe toxicities.^583,584^ As a result, flavopiridol was never tested in phase III trials and was eventually discontinued from manufacturing.^585^

Like flavopiridol, roscovitine is a broad-range inhibitor that targets CDK1, CDK2, CDK5, CDK7, and CDK9 (with IC50 values between 0.2-0.7 µM) but is a poor inhibitor for CDK4/6 and CDK8 (IC50 > 100 µM).^564,586^ Roscovitine has been tested across a wide variety of tumor cell lines, where it demonstrates two main effects: cell cycle arrest and initiation of apoptosis. By directly interacting with CDKs, roscovitine blocks the cell cycle at various phases (G0, G1, S, or G2/M), depending on the dose, duration, and cell line tested.^587,588^ Consequently, roscovitine also indirectly affects several signaling pathways, including Ras-MAPK, p53, estrogen receptor, and JAK-STAT. Additionally, it induces apoptosis by downregulating anti-apoptotic molecules (Bcl-2, Mcl-1, survivin, XIAP) and upregulating pro-apoptotic proteins (p53, p53AIP1).^589–592^

The anticancer activity of roscovitine has also been investigated in xenograft models, showing significant tumor growth reduction in various types of tumors, including colorectal, prostate, breast, and hematological cancers.^593–595^ Notably, roscovitine demonstrated efficacy in xenografts derived from breast cancer cells resistant to endocrine therapy.^596^

Encouraging preclinical results led to the testing of roscovitine in humans.^597^ In phase I dose-finding studies, roscovitine was found to be tolerable, but its anticancer efficacy was not prominent.^598,599^ In a follow-up blinded phase II trial (NCT00372073) involving 187 non-small cell lung cancer patients, roscovitine showed no improvement in progression-free survival (PFS),^577^ leading to the study’s termination without publication.

Besides malignant diseases, roscovitine was also tested in a phase II trial (NCT02649751) with 36 cystic fibrosis patients, but the study terminated without publication or public disclosure. Supported by preclinical evidence in animal models^600^ it was also tested in Cushing disease (hypercortisolism) (NCT02160730 and NCT03774446).^601^ Although limited by small number of participants (only 9 treated patients) due to the rarity of the disease, initial findings were promising and second arm of the multi-center study (NCT03774446) is still recruiting.

#### Development of second generation CDKs inhibitors

Dinaciclib is a second-generation CDK inhibitor (pyrazolo[1,5-a]pyrimidine-based purine analog) that selectively targets CDK1, CDK2, CDK5, and CDK9, with IC50 values in the nanomolar range. It is more specific, potent, and less toxic compared to the first-generation CDK inhibitor flavopiridol.^602,603^ Preclinical studies indicated that dinaciclib is equally effective as flavopiridol in inhibiting CDK1 and CDK9 but is a more potent inhibitor of CDK2 and CDK5 and a stronger inhibitor of DNA synthesis. In most tumor cells, dinaciclib blocks cell growth primarily by inhibiting CDK1 and CDK2 and promotes apoptosis by suppressing RB phosphorylation. Additionally, it affects CDK9 activation, leading to decreased gene transcription through RNA polymerase II. The consequences of CDK5 inhibition are less clear.^603–605^ The antitumor potency of dinaciclib was also confirmed in murine xenograft models, where it induced apoptosis and tumor regression across various tumor types.^603–605^

In phase I trials involving patients with advanced malignancies, dinaciclib demonstrated promising results, including tolerable toxicity and halted disease progression.^606–608^ However, an interim analysis of a randomized phase II trial with advanced breast cancer patients showed no superiority of dinaciclib over capecitabine, leading to the trial termination.^609^ Another phase II randomized study reported no benefit of dinaciclib treatment in non-small cell lung cancer.^610^ In melanoma, dinaciclib did not yield promising results, with no responses and common grade IV adverse events (NCT00937937).^611^

Some encouraging results were obtained in hematological diseases. In a phase I dose-escalation study with chronic lymphocytic leukemia (CLL), dinaciclib demonstrated clinical activity and manageable toxicities, including cytopenia and tumor lysis syndrome.^612^ In an accompanying phase Ib/II trial (NCT01515176), combining dinaciclib with ofatumumab (an anti-CD20 antibody) showed that prior exposure to ofatumumab prevented tumor lysis syndrome, allowing for the safe administration of dinaciclib.^613^ In another phase I trial (NCT01096342), dinaciclib showed encouraging results in relapsed multiple myeloma, with two out of 27 treated individuals achieving a deep response, and ten patients experiencing myeloma protein stabilization or decrease.^614^

In a randomized phase II trial involving patients with advanced acute leukemia, dinaciclib exhibited cytoreductive activity but did not achieve an objective response, frequently causing tumor lysis syndrome and diarrhea.^615^ Although the phase III randomized clinical trial (NCT01580228) was too immature for definitive conclusions due to the small number of participants (*n* = 42) and early termination, it suggested superiority of dinaciclib over ofatumumab in CLL. Dinaciclib improved progression-free survival (PFS, 13.7 vs. 5.9 months) and overall survival (OS, 21.2 vs. 16.7 months) compared to ofatumumab.^616^ Currently, there are no open phase III trials testing dinaciclib.

Another second-generation CDK inhibitor tested in clinical trials is AT7519, a pyrazole-3-carboxamide-based small molecule inhibitor of CDK1, CDK2, CDK4, and CDK6. An initial phase I study using a 1-hour intravenous infusion for 5 days showed that AT7519 was associated with cardiac polarization, evidenced by QTc prolongation.^617^ A revised administration schedule recommended intravenous injections on days 1, 4, 8, and 11, which demonstrated better tolerability and antitumor activity.^618^ Under this revised schedule, phase II trials were initiated with patients suffering from chronic lymphocytic leukemia (CLL) and mantle cell lymphoma (MCL). In these trials, AT7519 showed tumor shrinkage in both malignancies without causing tumor lysis syndrome. While no objective response was observed in CLL, a 27% response rate was noted in MCL.^619^ Another phase II study found that combining AT7519 with bortezomib was well tolerated and displayed a partial response in about 30% of patients.^620^

R547 is another small molecule inhibitor targeting CDK1, CDK2, CDK4, CDK7, and CDK9. It inhibits tumor cell proliferation by blocking the cell cycle at the G1 and G2 phases and inducing apoptosis.^621^ Its efficacy was observed independently of histotype, p53 status, and multidrug resistance status. In vivo, R547 demonstrated antitumor activity in a broad range of xenograft models. In a phase I trial with weekly intravenous infusion, R547 showed some antitumor activity with a manageable toxicity profile.^622^ Phase II trials in advanced solid tumors and hematologic malignancies were planned but not reported.

SNS-032 (also known as BMS-387032) is a potent and selective inhibitor of CDK2, CDK7, and CDK9, displaying suppression of cellular proliferation in several cell lines.^623^ In a phase I clinical trial with refractory solid tumors, patients treated with SNS-032 showed no signs of benefit, and treatment was discontinued in all patients due to relapse, disease progression, deterioration, and drug-related toxicities.^623,624^ The study was abandoned by Bristol-Myers Squibb but was later picked up by Sunesis Pharmaceuticals Inc. A phase I trial of SNS-032 in advanced CLL and multiple myeloma publicly disclosed minimal antitumor activity, with tumor lysis syndrome and a dose-limiting toxicity, leading to early closure of the study.^625^

The clinical development of AZD5438, an orally available CDK inhibitor with higher selectivity for CDK1, CDK2, and CDK9, over CDK5 and CDK6, was discontinued due to overwhelming intolerable side effects.^626^ Similarly, the development of AG-024322 was terminated prematurely as it failed to meet clinical endpoints and product profile expectations.

Despite the initial high hopes, pan-CDK inhibitors have largely failed to meet clinical expectations over approximately two decades. Several key factors contributed to this disappointing outcome. The high level of homology among CDKs made designing highly specific inhibitors challenging, leading to non-specific inhibition that hindered the understanding of which CDK inhibition drove therapeutic effects. Systemic administration of CDK inhibitors compromised vital physiological roles of CDKs, including cell cycle and transcriptional regulation, resulting in unintended consequences for normal tissues and numerous toxicities. None of the trials involving pan-CDK inhibitors were biomarker-driven, further complicating their clinical application. Commonly reported toxicities, such as nausea, diarrhea, and myelosuppression, consistently limited the prolonged use of these inhibitors.

NTogether, none of these inhibitors have obtained FDA approval, and only dinaciclib reached phase III trials. following

#### CDK4/6 inhibitors

While pan-CDK inhibitors were leaving the clinical stage, a new group of CDK inhibitors, specifically targeting CDK4 and CDK6 (hereafter CDK6/6), began to reshape the clinical practice, particularly for certain cancer types.^577,627,628^ The breakthrough in CDK inhibitor development came with achieving high specificity for CDK4/6. This selectivity was accomplished by incorporating a methyl group at the C-5 position of the pyridol[2,3-d]pyrimidin-7-one scaffold.^577^ Based on this finding, up to now, six CDK4/6 inhibitors were developed: palbociclib (PD-0332991, developed by Pfizer),^629^ ribociclib (LEE011, developed by Novartis),^630^ abemaciclib (LY-2835219, developed by Lilly),^631^ dalpiciclib (SHR6390, developed by Jiangsu Hengrui Medicine),^632^ trilaciclib (G1T28, developed by G1 Therapeutics Inc.)^633^ and lerociclib (GB491, developed by G1 Therapeutics Inc.).^634^

Currently, palbociclib, ribociclib, abemaciclib and trilaciclib have been approved by the FDA, dalpiclib has been approved by the NMPA but it is not available outside China, and lerociclib is undergoing clinical trials (Fig. 8).

Palbociclib and dalpiciclib have similar affinity for both CDK4 and CDK6.^629^ In contrast, ribociclib and abemaciclib display higher affinity for CDK4.^577^ Additionally, abemaciclib targets other kinases, including CDK9, PIM1, and HIPK2, and at higher doses, also CDK2.^635^ Trilaciclib and lerociclib have CDK9 as target, when administered at higher doses.^636^

##### Preclinical evidences of the activity of CDK4/6 inhibitors

The first CDK4/6 inhibitor to reach the clinic was palbociclib, originally described by Fry and Toogood in 2001.^629^ It took several years before phase II clinical trials began, in 2009.^637^ In their initial study, the authors demonstrated that palbociclib acted as an antiproliferative agent, causing cell cycle arrest in the G1 phase with a concomitant reduction in RB phosphorylation. Furthermore, in vivo experiments with mice bearing human colon carcinoma xenografts proved that inhibition of CDK4/6 alone was sufficient to induce marked tumor regression.^629^

The preclinical work that paved the way for testing palbociclib in humans was conducted by Finn and colleagues, who tested its growth inhibitory effects on a large panel of human breast cancer cell lines.^638^ They identified potent activity in estrogen receptor-positive cells, including those that were HER2-amplified, while basal-like cell lines were the most insensitive to the drug. Notably, they found that palbociclib had synergistic growth inhibitory activity when combined with the anti-estrogen drug tamoxifen and demonstrated efficacy in a model of acquired tamoxifen resistance.^638^ Further in vitro studies showed that palbociclib, as well as other CDK4/6 inhibitors such as ribociclib and abemaciclib, were effective in blocking cell proliferation in several RB-positive cancer cell lines, but not in cell lines that had lost RB expression. This is consistent with the understanding that the principal rate-limiting substrate of CDK4/6 is RB.^629,639^

CDK4/6 inhibitors were extensively tested in vivo, demonstrating antitumor activity in various xenograft models, including those of solid and hematological diseases.^629,633,640^ Both palbociclib and abemaciclib are capable of crossing the blood-brain barrier and inhibiting the growth of intracranial glioblastoma xenografts.^641^ Also in most mouse models, the therapeutic effect was dependent on the expression of RB protein.

Palbociclib sensitivity was found to increase with the loss of p16^INK4A^, p15^INK4B^, E2F1, or low cyclin E1 expression, while amplification of the CCNE1 gene or overexpression of E2F2 decreased sensitivity. The effect of CDK4 amplification on CDK4/6 inhibitor sensitivity remains unclear.^642,643^ Mechanistically, it was initially assumed that palbociclib and other CDK4/6 inhibitors act by directly inhibiting the cyclin D-CDK4/6 complex. However, recent evidence suggests that palbociclib inhibits only cyclin D-CDK4/6 dimers and not the trimeric cyclin D-CDK4/6-p27 complex.^644^ Additionally, it has been suggested that CDK4/6 inhibitors function in two ways: a non-catalytic role that indirectly inactivates CDK2 complexes via p21 redistribution and a catalytic role that directly inactivates CDK4 and CDK6 kinase activity.^645^ This dual mechanism requires further evaluation.

Another controversial observation is that palbociclib, ribociclib, and abemaciclib impair the binding of CDK4/6 to CDC37, thus preventing access to the HSP90-chaperone system.^646^ Since the HSP90-CDC37 complex stabilizes several kinases, this implies that CDK4/6 inhibitors should promote CDK4/6 degradation—a phenomenon not typically observed with these treatments.^643^

A well-documented effect of CDK4/6 inhibitors is the induction of tumor cell senescence, which depends on RB and FOXM11 activity. The role of RB in senescence is well known, and FOXM11 represents a CDK4/6 target with anti-senescence properties.^65,643^ This aspect is clinically relevant because senescence induction could trigger an immune response leading to tumor clearance. However, the release of the senescence-associated secretory phenotype (SASP) by senescent cancer cells could promote chronic inflammation in the tumor, leading to deleterious consequences.^647^ The determinants of the extent of senescence induced by CDK4/6 inhibitors are not fully understood. Some evidence suggests that CDK4/6 inhibition leads to an RB-dependent increase in reactive oxygen species (ROS) levels, resulting in the activation of autophagy and limitation of senescence in breast cancer cells.^648^

##### CDK4/6 inhibitors in Hormone Receptor positive (HR+) HER2 negative (HER-) advanced breast cancer

Preclinical results in breast cancer triggered the beginning of the randomized Phase II trial (PALOMA-1) involved 165 advanced-stage HR+ and HER2- postmenopausal breast cancer patients who had not received prior systemic treatment.^637^ The study demonstrated that combining palbociclib with the aromatase inhibitor letrozole significantly improved median progression-free survival (PFS) to 20.2 months, compared to 10.2 months with letrozole alone. Based on these striking results, the FDA granted accelerated approval for the combination treatment in the first-line setting, in 2015.^649^

This successful clinical experience led the development of several other CDK4/6 inhibitors and trials (Table 3) that resulted, up to now, in the accelerated approval by regulatory agencies of their use in HR+ breast cancer patients. Further preclinical and clinical researches are necessary to understand if CDK4/6 inhibitors could be successfully used also in other cancer types of cancer and/or other human diseases.

**Table 3:** Clinical trials using CDK4/6 inhibitors in human cancer

CDK4/6 inhibitors in Hormone Receptor positive (HR+) HER2 negative (HER-) advanced breast cancer							
Study name	Interventions	CDK-Inhibitor	Phase	Status	NCT	PMID/DOI	REF
Study Of Letrozole With Or Without Palbociclib (PD-0332991) For The First-Line Treatment Of Hormone-Receptor Positive Advanced Breast Cancer (PALOMA-1/TRIO-18)	**Arm A**: Letrozole (orally on a continuous regimen) + Palbociclib (orally for 3 out of 4 weeks in repeated cycles) **Arm B**: Letrozole (orally on a continuous regimen)	CDK4/6 - Palbociclib	II	Completed	NCT00721409	25524798 10.1016/S1470-2045(14)71159-3	^637^
A Study of Palbociclib (PD-0332991) + Letrozole versus Letrozole For 1st Line Treatment Of Postmenopausal Women With ER + / HER2- Advanced Breast Cancer (PALOMA-2)	**Arm A**: Letrozole (orally once daily, continuously) + Palbociclib (orally once daily on Day 1 to Day 21 of every 28-day cycle followed by 7 days off treatment) **Arm B**: Letrozole (orally once daily, continuously) + placebo (orally once daily on Day 1 to Day 21 of every 28-day cycle followed by 7 days off treatment)	CDK4/6 - Palbociclib	III	Completed	NCT01740427	27959613 10.1056/NEJMoa1607303	^650^
Palbociclib (PD-0332991) Combined With Fulvestrant In Hormone Receptor+ HER2-Negative Metastatic Breast Cancer After Endocrine Failure (PALOMA-3)	**Arm A**: Fulvestrant (orally continuously dosed for 3 weeks followed by 1 week off; repeated at each subsequent cycle) + Palbociclib (orally continuously dosed for 3 weeks followed by 1 week off; repeated at each subsequent cycle) **Arm B**: Fulvestrant (orally continuously dosed for 3 weeks followed by 1 week off; repeated at each subsequent cycle) + Placebo (orally continuously dosed for 3 weeks followed by 1 week off; repeated at each subsequent cycle)	CDK4/6 - Palbociclib	III	Completed	NCT01942135	26030518 10.1056/NEJMoa1505270 26947331 10.1016/S1470-2045(15)00613-0	^651,652^
A Study of Abemaciclib (LY2835219) In Participants With Previously Treated Breast Cancer That Has Spread (MONARCH 1)	Abemaciclib given orally once every 12 hours for 28 days (1 cycle)	CDK4/6 - Abemaciclib	II	Completed	NCT02102490	28533223 10.1158/1078-0432.CCR-17-0754	^653,654^
A Study of Abemaciclib (LY2835219) Combined With Fulvestrant in Women With Hormone Receptor Positive HER2 Negative Breast Cancer (MONARCH 2)	**Arm A**: Abemaciclib (orally every 12 hours on Days 1 to 28 of a 28-day cycle) + Fulvestrant (intramuscularly on Days 1 and 15 of Cycle 1, then on Day 1 of Cycle 2 and beyond) **Arm B**: placebo (orally every 12 hours on Days 1 to 28 of a 28-day cycle) + Fulvestrant (intramuscularly on Days 1 and 15 of Cycle 1, then on Day 1 of Cycle 2 and beyond)	CDK4/6 - Abemaciclib	III	Active, not recruiting	NCT02107703	28580882 10.1200/JCO.2017.73.7585 10.1158/1538-7445.SABCS22-PD13-11	^657,658^
A Study of Nonsteroidal Aromatase Inhibitors Plus Abemaciclib (LY2835219) in Postmenopausal Women With Breast Cancer (MONARCH 3)	**Arm A**: Abemaciclib (orally every 12 hours) + Anastrozole or Letrozole (orally once daily for 28 days, 28-days cycles) **Arm B**: placebo (orally every 12 hours) + Anastrozole or Letrozole (orally once daily for 28 days, 28-days cycles)	CDK4/6 - Abemaciclib	III	Active, not recruiting	NCT02246621	28968163 10.1016/j.annonc.2022.08.009	^655,656^
Study of Efficacy and Safety of LEE011 in Postmenopausal Women With Advanced Breast Cancer. (MONALEESA-2)	**Arm A**: Ribociclib (orally, 3 weeks on/1 week off) + Letrozole (orally once daily) **Arm B**: placebo (orally, 3 weeks on/1 week off) + Letrozole (orally once daily)	CDK4/6 - Ribociclib	III	Completed	NCT01958021	27717303 10.1056/NEJMoa1609709 35263519 10.1056/NEJMoa2114663 10.1158/1538-7445.SABCS22-PD13-11	^659,660^
Study of Efficacy and Safety of LEE011 in Men and Postmenopausal Women With Advanced Breast Cancer. (MONALEESA-3)	**Arm A**: Ribociclib (orally, 21 consecutive days within a 28-day cycle) + Fulvestrant (intramuscularly every 28 days starting on Day 1 of each cycle). Additionally, an extra dose of Fulvestrant was given on Day 15 of Cycle 1 **Arm B**: placebo (orally, 21 consecutive days within a 28-day cycle) + Fulvestrant (intramuscularly every 28 days starting on Day 1 of each cycle). Additionally, an extra dose of Fulvestrant was given on Day 15 of Cycle 1	CDK4/6 - Ribociclib	III	Completed	NCT02422615	29860922 10.1200/JCO.2018.78.9909 34102253 10.1016/j.annonc.2021.05.353	^661,662^
Study of Efficacy and Safety in Premenopausal Women With Hormone Receptor Positive, HER2-negative Advanced Breast Cancer (MONALEESA-7)	**Arm A**: Ribociclib (orally, 3 weeks on/1 week off) + NSAI or Tamoxifen (daily oral) and Goserelin (subcutaneous, once every 28 days) **Arm B**: placebo (orally, 3 weeks on/1 week off) + NSAI or Tamoxifen (daily oral) and Goserelin (subcutaneous, once every 28 days)	CDK4/6 - Ribociclib	III	Completed	NCT02278120	29804902 10.1016/S1470-2045(18)30292-4 10.1200/JCO.2019.37.18_suppl.LBA1008	^665,666^
A Study of SHR6390 in Combination With Fulvestrant in Patients With HR Positive and HER2 Negative Advanced Breast Cancer (DAWNA-1)	**Arm A**: SHR6390 (orally daily on day 1 to day 21 of every 28-day cycle followed by 7 days off treatment) + Fulvestrant (intramuscularly on day 1 and day 15 for the first cycle and then on day 1 for every cycle until progressive disease) **Arm B**: placebo (orally daily on day 1 to day 21 of every 28-day cycle followed by 7 days off treatment) + Fulvestrant (intramuscularly on day 1 and day 15 for the first cycle and then on day 1 for every cycle until progressive disease)	CDK4/6 - Dalpiciclib	III	Unknown	NCT03927456	34737452 10.1038/s41591-021-01562-9	^663^
A Study of SHR6390 in Combination With Letrozole or Anastrozole in Patients With HR Positive and HER2 Negative Advanced Breast Cancer (DAWNA-2)	**Arm A**: SHR6390 + Lestrozole or Anastrozole **Arm B**: placebo + Lestrozole or Anastrozole	CDK4/6 - Dalpiciclib	III	Unknown	NCT03966898	37182538 10.1016/S1470-2045(23)00172-9	^664^
GB491 Combined With Fulvestrant for HR+/HER2- Locally Advanced or Metastatic Breast Cancer (LEONARDA-1)	**Arm A**: GB491 (orally, twice a day) + Fulvestrant (intramuscularly, on day 1 and day 15 of the first cycle, and on day 1 of the second and subsequent cycles) **Arm B**: placebo (orally, twice a day) + Fulvestrant (intramuscularly, on day 1 and day 15 of the first cycle, and on day 1 of the second and subsequent cycles)	CDK4/6 - Lerociclib	III	Unknown	NCT05054751	10.1200/JCO.2023.41.16_suppl.101	^634^
**CDK4/6 inhibitors as adjuvant and neo-adjuvant therapies in breast cancer**							
Palbociclib CoLlaborative Adjuvant Study (PALLAS)	Arm A: Palbociclib (orally once daily, day 1 to day 21 followed by 7 days off treatment in a 28-day cycle for a total duration of 2 years) + standard adjuvant endocrine therapy for a duration of at least 5 years Arm B: standard adjuvant endocrine therapy for a duration of at least 5 years	CDK4/6 - Palbociclib	III	Active, not recruiting	NCT02513394	33460574 10.1016/S1470-2045(20)30642-2 34874182 10.1200/JCO.21.02554	^667,668^
A Study of Palbociclib in Addition to Standard Endocrine Treatment in Hormone Receptor Positive Her2 Normal Patients With Residual Disease After Neoadjuvant Chemotherapy and Surgery (PENELOPE-B)	Arm A: Palbociclib (orally daily, day 1 to day 21 followed by 7 days off treatment in a 28-day cycle) Arm B: placebo (once daily, day 1 to day 21 followed by 7 days off treatment in a 28-day cycle)	CDK4/6 - Palbociclib	III	Completed	NCT01864746	33793299 10.1200/JCO.20.03639	^669^
A Trial to Evaluate Efficacy and Safety of Ribociclib With Endocrine Therapy as Adjuvant Treatment in Patients With HR+/ HER2- Early Breast Cancer (NATALEE)	**Arm A**: Ribociclib (orally daily, days 1 to 21 of a 28-day cycle) + endocrine therapy according to the local clinical guidelines and current local prescribing information **Arm B**: endocrine therapy according to the local clinical guidelines and current local prescribing information	CDK4/6 - Ribociclib	III	Active, not recruiting	NCT03701334	10.1200/JCO.2023.41.17_suppl.LBA500 37275963 10.1177/17588359231178125	^670,713^
Endocrine Therapy With or Without Abemaciclib (LY2835219) Following Surgery in Participants With Breast Cancer (monarchE)	**Arm A**: Abemaciclib (orally, twice daily for up to 2 years or until evidence of disease recurrence or other discontinuation) + endocrine therapy (administered according to manufacturer instructions) **Arm B**: endocrine therapy (administered according to manufacturer instructions)	CDK4/6 - Abemaciclib	III	Active, not recruiting	NCT03155997	32954927 10.1200/JCO.20.02514 36493792 10.1016/S1470-2045(22)00694-5	^671,672^
Efficacy of Letrozole + Palbociclib Combination as Neoadjuvant Treatment of Stage II-IIIA PAM 50 ROR-defined Low or Intermediate Risk Luminal Breast Cancer, in Postmenopausal Women (NeoPAL)	**Arm A**: 3 cycles of FEC 100 followed by 3 cycles of docetaxel drugs (Fluorouracile, Epirubicine, Cyclophosphamide, Docetaxel) **Arm B**: Letrozole + Palbociclib	CDK4/6 - Palbociclib	II	Completed	NCT02400567	30307466 10.1093/annonc/mdy448 35337692 10.1016/j.ejca.2022.01.014	^673,674^
Neadjuvant Multi-agent Chemotherapy or Letrozole Plus Ribociclib in Luminal B/ HER2-negative Breast Cancer. (CORALLEEN)	**Arm A**: Ribociclib (flat-fixed dose days 1 to 21 of a 28-days cycle) + Letrozole (daily continuous) **Arm B**: four cycles of AC (Doxorubicin and Cyclophosphamide every 21 days) + weekly paclitaxel during 12 weeks	CDK4/6 - Ribociclib	II	Completed	NCT03248427	31838010 10.1016/S1470-2045(19)30786-7	^675^
Letrozole Plus Ribociclib or Placebo as Neo-adjuvant Therapy in ER-positive, HER2-negative Early Breast Cancer (FELINE)	**Arm A**: placebo (3 capsules/day 3 weeks on/1week off or 2 capsules/day continuous dosing) + Letrozole (daily) **Arm B**: Ribociclib (daily 21 days on/7 days off) + Letrozole (daily) **Arm C**: Ribociclib (continuous daily) + Letrozole (daily)	CDK4/6 - Ribociclib	II	Active, not recruiting	NCT02712723	10.1200/JCO.2020.38.15_suppl.505	^676^
A Phase II Randomized Study Evaluating the Biological and Clinical Effects of the Combination of Palbociclib With Letrozole as Neoadjuvant Therapy in Post-Menopausal Women With Estrogen-Receptor Positive Primary Breast Cancer (PALLET)	**Arm A**: Letrozole (orally daily for 14 weeks) **Arm B**: Letrozole (orally daily) + beginning 2 weeks after starting Letrozole + Palbociclib (orally daily for 1 week then 1 week off, then a 3-week on and 1-week off cycle for a total of 14 weeks from start of Letrozole therapy) **Arm C**: Palbociclib (orally daily, for a 3 weeks on and 1 week off cycle for a total of 14 weeks from start of Palbociclib) + beginning 2 weeks after starting Palbociclib, Letrozole (orally daily for a total of 12 weeks from start of Letrozole therapy) Arm D: Letrozole (orally daily for a total of 14 weeks) + Palbociclib (orally daily for a 3 weeks on and 1 week off cycle, for a total of 14 weeks from start of therapy)	CDK4/6 - Palbociclib	II	Completed	NCT02296801	30523750 10.1200/JCO.18.01624	^677^
PD 0332991 and Anastrozole for Stage 2 or 3 Estrogen Receptor Positive and HER2 Negative Breast Cancer (NeoPalAna)	**Arm A:** (**PIK3CA Wild Type Cohort), Arm B: (PIK3CA Mutant Type Cohort), Arm C: (Endocrine Resistant Cohort)**: Cycle 0 is 28 days of Anastrozole (orally daily) + Goserelin (subcutaneously, every 28 days) if premenopausal Cycles 1-5 are Palbociclib + Anastrozole (and Goserelin if premenopausal) is to be (4) 28-day cycles followed by a 5th cycle of 10-12 days duration consisting of Palbociclib (daily) + Anastrozole (last dose day before surgery) Standard surgery will be performed per institutional standards 2-4 weeks following the completion of Cycle 4 in those who did not receive Cycle 5. In pts who receive Cycle 5, surgery occurs on Day 11, 12, or 13 of Cycle 5 Pts who derived benefit from the therapy have the option of taking Palbociclib + endocrine therapy for 23 cycles after surgery and adjuvant chemotherapy and radiation if indicated. It should be re-started at least 4 weeks after the completion of chemotherapy and radiation therapy if these treatments were planned	CDK4/6 – Palbociclib	II	Active, not recruiting	NCT01723774	28270497 10.1158/1078-0432.CCR-16-3206	^678^
Neoadjuvant Letrozole and Palbociclib in Patients With Stage II-IIIB Breast Cancer, HR+/HER2 - (DxCARTES)	**Arm A (pts with recurrence score 18-25), Arm B (pts with recurrence score 26-100)**: pretreatment with Palbociclib + Letrozole; Letrozole (during 28 days of each Cycle) + Palbociclib (during 21 days of each cycle of 28 days) as neoadjuvant treatment during 6 cycles before surgery	CDK4/6 – Palbociclib	II	Completed	NCT03819010	10.1158/1538-7445.SABCS21-P5-16-12	^679^
A Neoadjuvant Study of Abemaciclib (LY2835219) in Postmenopausal Women With Hormone Receptor Positive, HER2 Negative Breast Cancer (neoMONARCH)	**Arm A**: Abemaciclib (orally every 12) + Anastrozole (orally daily) for 2 weeks. Loperamide was given as a prophylaxis for 4 weeks and then at physician discretion. All participants received Abemaciclib + Anastrozole for an additional 14 weeks. Total treatment duration was 16 weeks. **Arm B**: Abemaciclib (orally every 12) for 2 weeks. Loperamide was given as a prophylaxis for 4 weeks and then at physician discretion. All participants received Abemaciclib + Anastrozole for an additional 14 weeks. Total treatment duration was 16 weeks. **Arm C**: Anastrozole (orally daily) for 2 weeks. Loperamide was given as a prophylaxis for 4 weeks and then at physician discretion. All participants received Abemaciclib + Anastrozole for an additional 14 weeks. Total treatment duration was 16 weeks.	CDK4/6 - Abemaciclib	II	Completed	NCT02441946	31615937 10.1158/1078-0432.CCR-19-1425	^680^
A Study of Abemaciclib (LY2835219) in Participants With Non-Small Cell Lung Cancer or Breast Cancer	**Arm A (HR**+**, HER2- Metastatic Breast Cancer), Arm B (HR**+**, HER2- Locally Advanced or Metastatic Breast Cancer):** Abemaciclib (orally) on days 1 to 21 of each 21 days cycle in combination with Pembrolizumab given intravenously on day 1 of each 21-day cycle.	CDK4/6 - Abemaciclib	Ib	Active, not recruiting	NCT02779751	36335120 10.1038/s41523-022-00482-2	^683^
Pembrolizumab, Endocrine Therapy, and Palbociclib in Treating Postmenopausal Patients With Newly Diagnosed Metastatic Stage IV Estrogen Receptor Positive Breast Cancer	**Arm A:** Pts receive either Letrozole (orally daily) on days -28 to -1 and days 1-28, or Fulvestrant on days -28, -14, and day 1 of subsequent cycles. Pts also receive Palbociclib (orally daily) for 3 weeks. Cycles with Palbociclib, and Letrozole or Fulvestrant repeat every 28 days in the absence disease progression or unacceptable toxicity. Patients also receive Pembrolizumab intravenously on day 1. Cycles with Pembrolizumab repeat every 21 days in the absence of disease progression or unacceptable toxicity. **Arm B:** Pts receive Letrozole (orally daily) on days 1-28 and Palbociclib (orally daily) for 3 weeks. Cycles with Letrozole and Palbociclib repeat every 28 days in the absence of disease progression or unacceptable toxicity. Pts also receive Pembrolizumab intravenously on day 1. Cycles with Pembrolizumab repeat every 21 days in the absence of disease progression or unacceptable toxicity.	CDK4/6 – Palbociclib	II	Recruiting	NCT02778685	34217908 10.1016/j.ejca.2021.05.035	^684^
Palbociclib After CDK and Endocrine Therapy (PACE)	**Arm A:** Fulvestrant (intramuscularly) on Cycle 1 Days 1, 15, then monthly **Arm B**: Palbociclib (orally daily for 21 days on a 28-day cycle) + Fulvestrant (intramuscularly) on Cycle 1 Days 1, 15, then monthly	CDK4/6 – Palbociclib	II	Active, not recruiting	NCT03147287	38513188 10.1200/JCO.23.01940	^685^
**CDK4/6 inhibitors in other breast cancer types**							
Neo-Adjuvant Treatment With Palbociclib: Effect on Ki67 and Apoptosis Before, During and After Treatment (NA-PHER2)	**Arm A**: Trastuzumab, Pertuzumab, Palbociclib plus or minus Fulvestrant as neoadjuvant chemotherapy. Definitive surgery will be performed not earlier than 14 days and not later than 28 days after the last dose of any of the drugs in the combination. After completion of surgical treatment pts will receive irradiation as locally acceptable.	CDK4/6 – Palbociclib	II	Completed	NCT02530424	29326029 10.1016/S1470-2045(18)30001-9 35013314 10.1038/s41523-021-00377-8	^686,687^
Study of Palbociclib and Trastuzumab With Endocrine Therapy in HER2-positive Metastatic Breast Cancer (PATRICIA II)	**Arm A HER2**+**/HR-:** Palbociclib (orally, daily for 2 weeks, followed by 1 week off) + Trastuzumab (intravenously loading dose then every 3 weeks or subcutaneously every 3 weeks) **Arm B1 HER2**+**/HR**+**:** Palbociclib (orally, daily for 2 weeks, followed by 1 week off) + Trastuzumab (intravenously loading dose then every 3 weeks or subcutaneously every 3 weeks) **Arm B2 HER2**+**/HR**+**:** Palbociclib (orally, daily for 2 weeks, followed by 1 week off) + Trastuzumab (intravenously loading dose then every 3 weeks or subcutaneously every 3 weeks) + Letrozole (daily orally) **Arm C1 HER2**+**/HR+ Luminal intrinsic subtype determined by PAM50:** Palbociclib (orally for 3 weeks, followed by one week off, in 4-week cycles) + Trastuzumab (intravenously loading dose then every 3 weeks or subcutaneously every 3 weeks) + AI or Fulvestrant or Tamoxifen **Arm C2 HER2**+**/HR+ Luminal intrinsic subtype determined by PAM50:** treatment based on physician’s choice from the following options: TDM1 or chemotherapy (Gemcitabine, Vinorelbine, Capecitabine, Eribulin or a Taxane) + Trastuzumab or endocrine therapy (aromatase inhibitor, Fulvestrant or Tamoxifen) in combination with Trastuzumab	CDK4/6 – Palbociclib	II	Completed	NCT02448420	32938620 10.1158/1078-0432.CCR-20-0844	^688^
PAveMenT: Palbociclib and Avelumab in Metastatic AR+ Triple Negative Breast Cancer (PAveMenT)	**Arm A**: dose escalation of Palbociclib + fixed dose Avelumab **Arm B**: treatment with the maximum tolerated dose and schedule established in Arm A	CDK4/6 – Palbociclib	Ib	Recruiting	NCT04360941	10.1158/1538-7445.SABCS22-P4-01-02	^714^
Randomized, Open Label, Clinical Study of the Targeted Therapy, Palbociclib, to Treat Metastatic Breast Cancer (PATINA)	**Arm A:** Palbociclib (daily) + Trastuzumab or pertuzumab (every 3 weeks) + Letrozole, Anastrozole, Exemstane or Fulvestrant until confirmed disease progression **Arm B**: Trastuzumab or pertuzumab (every 3 weeks) + Letrozole, Anastrozole, Exemstane or Fulvestrant until confirmed disease progression	CDK4/6 – Palbociclib	III	Active, not recruiting	NCT02947685	10.1158/1538-7445.SABCS18-OT3-02-07	^689^
A Study of Abemaciclib (LY2835219) in Women With HR + , HER2+ Locally Advanced or Metastatic Breast Cancer (monarcHER)	**Arm A**: Abemaciclib (orally daily of a 21-day cycle) + Trastuzumab (intravenously on day 1 of the cycle then on day 1 of each subsequent cycle) + Fulvestrant (intramuscularly on day 1, 15 and 29 and then once every 4 weeks thereafter) **Arm B**: Abemaciclib (orally daily of a 21-day cycle) + Trastuzumab (intravenously on day 1 of the cycle then on Day 1 of each subsequent cycle) **Arm C**: Trastuzumab (intravenously on day 1 of a 21-day cycle then on Day 1 of each subsequent cycle) + standard of care single agent chemotherapy of physician’s choice administered according to product label	CDK4/6 - Abemaciclib	II	Completed	NCT02675231	32353342 10.1016/S1470-2045(20)30112-1 37906649 10.1158/1078-0432.CCR-23-1209	^690,691^
Palbociclib and Circulating Tumor DNA for ESR1 Mutation Detection (PADA-1)	**Step 1:** 1000 pts screened for circulating blood ESR1 mutation detection at regular intervals will be treated with Palbociclib (daily, or 21 days followed by 7 days off, 28-day cycle) + Letrozole, Anastrozole or exemestane administered (daily) in continuous scheme until tumor progression or ESR1 mutation detection **Step 2:** Up to 200 pts with a rising circulating ESR1 mutation and without tumor progression will be randomized (1:1): **Arm A**: no change in therapy until tumor progression or possibility of a cross-over (step 3) **Arm B**: Palbociclib + Fulvestrant (intramuscularly on day 1,15 and 29 and once monthly thereafter until tumor progression) **Step 3** (cross over): up to 80 pts who have been randomized in Arm A will be offered to be treated by Fulvestrant + Palbociclib, after having progressed under AI + Palbociclib until tumor progression (RECIST) under Fulvestrant + Palbociclib.	CDK4/6 – Palbociclib	III	Active, not recruiting	NCT03079011	36183733 10.1016/S1470-2045(22)00555-1	^715^
Trilaciclib (G1T28), a CDK 4/ 6 Inhibitor, in Combination With Gemcitabine and Carboplatin in Metastatic Triple Negative Breast Cancer (mTNBC)	**Arm A**: Gemcitabine + Carboplatin (intravenously on Days 1 and 8 of 21-day cycle) **Arm B:** Trilaciclib + Gemcitabine/ Carboplatin (intravenously on days 1 and 8 of 21-day cycle). Trilaciclib was administered prior to chemotherapy **Arm C**: Trilaciclib + Gemcitabine (intravenously on Day 2 and 9 of 21-day cycle). Trilaciclib was administered prior to chemotherapy.	CDK4/6 – Trilaciclib	II	Terminated	NCT02978716	34887261 10.1158/1078-0432.CCR-21-2272	^692^
Trilaciclib, a CDK 4/ 6 Inhibitor, in Patients Receiving Gemcitabine and Carboplatin for Metastatic Triple-Negative Breast Cancer (TNBC) (PRESERVE 2)	**Arm A**: Trilaciclib (intravenously on day 1 and day 8 of each 21-day cycle) + Gemcitabine (intravenously on day 1 and day 8 of each 21-day cycle) + Carboplatin (intravenously on day 1 and day 8 of each 21-day cycle) **Arm B**: placebo (intravenously on day 1 and day 8 of each 21-day cycle) + Gemcitabine (intravenously on day 1 and day 8 of each 21-day cycle) + Carboplatin (intravenously on day 1 and day 8 of each 21-day cycle)	CDK4/6 – Trilaciclib	III	Completed	NCT04799249	36135712 10.2217/fon-2022-0773	^693^
**CDK4/6 inhibitors in other tumors**							
A Phase 1 Study of LY2835219 In Participants With Advanced Cancer	**Arm A:** Abemaciclib (orally, daily for 28-day cycles for two planned cycles). For Part G only in addition to Abemaciclib as above, Fulvestrant is administered as specified in the label.	CDK4/6 - Abemaciclib	I	Completed	NCT01394016	27217383 10.1158/2159-8290.CD-16-0095	^694^
A Study of Abemaciclib (LY2835219) in Participants With Previously Treated KRAS Mutated Lung Cancer (JUNIPER)	**Arm A**: Abemaciclib (orally daily on Days 1 to 28, 28-day cycles) **Arm B:** Erlotinib (orally daily on Days 1 to 28, 28-day cycles).	CDK4/6 - Abemaciclib	III	Active, not recruiting	NCT02152631	33194700 10.3389/fonc.2020.578756	^695^
Adagrasib in Combination With Palbociclib in Patients With Advanced Solid Tumors (KRYSTAL-16)	**Arm A**: dose escalation of MRTX849 and Palbociclib to determine maximum tolerated dose in combination **Arm B**: expansion cohorts may be implemented to ensure sufficient safety experience, pharmacokinetic data and early evidence of clinical activity of MRTX849 in combination with Palbociclib.	CDK4/6 – Palbociclib	I/Ib	Active, not recruiting	NCT05178888	35167329 10.1200/JCO.21.02752	^699^
PD 0332991 and Cetuximab in Patients With Incurable SCCHN	**Phase I, Dose Level 1:** Palbociclib (orally daily from day 1 to 21, 28-days cycle) + Cetuximab (intravenously, weekly for the duration of their participation on study) **Phase I, Dose Level 2:** Palbociclib (orally daily from day 1 to 21, 28-days cycle) + Cetuximab (intravenously, weekly for the duration of their participation on study) **Phase II Arm 1: Platin-Resistant HPV-Unrelated SCCHN**. Palbociclib (orally daily from day 1 to 21, 28-days cycle) + Cetuximab (intravenously, weekly for the duration of their participation on study) **Phase II Arm 2: Cetuximab-Resistant HPV-Unrelated SCCHN**. Palbociclib (orally daily from day 1 to 21, 28-days cycle) + Cetuximab (intravenously, weekly for the duration of their participation on study) **Phase II Arm 3**. Palbociclib (orally daily from day 1 to 21, 28-days cycle) + Cetuximab (intravenously, weekly for the duration of their participation on study)	CDK4/6 – Palbociclib	I/II	Completed	NCT02101034	31351869^700^	.^700^
Avelumab, Cetuximab, and Palbociclib in Recurrent or Metastatic Head and Neck Squamous Cell Carcinoma	**Arm A**: identify the maximum tolerated dose (MTD) or recommended phase II dose (RP2D) for the combination of Palbociclib, Avelumab, and Cetuximab	CDK4/6 – Palbociclib	I	Active, not recruiting	NCT03498378	36279618^705^	^705^
Ibrutinib and Palbociclib in Treating Patients With Previously Treated Mantle Cell Lymphoma	**Arm A:** Ibrutinib (orally daily on days 1-28) + Palbociclib (orally daily on days 1-21). Cycles repeat every 28 days in the absence of disease progression or unacceptable toxicity	CDK4/6 – Palbociclib	I	Active, not recruiting	NCT02159755	30692121 10.1182/blood-2018-11-886457	
A Study of Voruciclib Alone or in Combination With Venetoclax in Subjects With B-Cell Malignancies or AML	**Arm A**: Voruciclib monotherapy – Open-label, 3 + 3 dose escalation study which may enroll up to 6 subjects at each dose level and disease type (AML or B-cell malignancies) **Arm B**: Voruciclib + Venetoclax - Open-label, 3 + 3 dose escalation study which may enroll up to 6 subjects at each dose level for AML subjects	CDK9 - Voruciclib	I	Recruiting	NCT03547115	10.1182/blood-2023-179577	
Study of SLS009 (Formerly GFH009) a Potent Highly Selective CDK9 Inhibitor in Patients With Hematologic Malignancies	**Group 1. Dose escalation in pts with r/r AML, Group 2. Dose escalation in pts with r/r CLL/SLL or lymphoma:** in the dose escalation part, the dose levels will be escalated following the Bayesian optimal interval (BOIN) design. **Group 3 Cohort 1. 45** **mg once a week in pts with r/r AML**. SLS009 + Venetoclax and Azacitidine in pts with r/r AML who have relapsed on or are refractory to Venetoclax-based regimens. **Group 3 Cohort 2. 60** **mg once a week in pts with r/r AML**. SLS009 + Venetoclax and Azacitidine in pts with r/r AML who have relapsed on or are refractory to Venetoclax-based regimens. **Group 3 Cohort 3. 30** **mg twice/week in pts with r/r AML**. SLS009 + Venetoclax and Azacitidine in pts with r/r AML who have relapsed on or are refractory to Venetoclax-based regimens. **Group 3 Cohort 4. 30** **mg twice/weekly in pts with r/r AML with ASXL1 mutation**. SLS009 + Venetoclax and Azacitidine in pts with r/r AML who have relapsed or are refractory to Venetoclax-based regimens and with documented ASXL1 mutation. **Group 3 Cohort 5. 30** **mg twice/weekly in pts with r/rAML with other than ASXL1 mutations**. SLS009 + Venetoclax and Azacitidine in pts with r/r AML who have relapsed or are refractory to Venetoclax-based regimens and with documented defining somatic mutations, cytogenetic abnormalities defining acute myeloid leukemia, myelodysplasia related, other than ASXL1 mutation.	CDK9 - SLS009	I/IIa	Recruiting	NCT04588922		
A Study to Investigate Fadraciclib (CYC065), in Subjects With Advanced Solid Tumors and Lymphoma	**Phase I**. Fadraciclib (orally) in escalating doses for 3 weeks of a 4-week cycle. Subsequent cohorts will escalate in dose and schedule until optimized phase 2 dose and schedule is achieved. **Phase 2**. Recommended Fadraciclib phase 2 dose and schedule administered orally in 28-day cycles.	CDK2/9 - Fadraciclib	I/II	Recruiting	NCT04983810	10.1200/JCO.2024.42.16_suppl.3125	
Highly Selective CDK7 Inhibitor Q901 in Selected Advanced Solid Tumors	**Arm A: Dose escalation, Arm B: Single-Agent Expansion Cohorts:** Once a week for 4 weeks, followed by a two weeks interval. **Arm C**: Q901 (once a week for 4 weeks, followed by a two-week interval) + Pembrolizumab (every six weeks)	CDK7 - Q901	I/II	Recruiting	NCT05394103	10.1200/JCO.2024.42.16_suppl.3078	
Open-Label Study to Evaluate the Safety, Tolerability, PK, and Efficacy of INX-315 in Patients With Advanced Cancer (INX-315-01)	**Arm A, Dose escalation:** Multiple doses of INX-315 monotherapy (orally) **Arm B, Ovarian Dose Expansion:** INX-315 monotherapy (orally) **Arm C, ER** + **/HER2- BC Dose Expansion:** INX-315 in combination with CDK4/6i and endocrine therapy, oral administration	CDK2 - INX-315	I/II	Recruiting	NCT05735080		
RVU120 for Treatment of Anemia in Patients With Lower-risk Myelodysplastic Neoplasms (MDS)	**Arm A:** RVU120 (orally daily. in a 21-day treatment cycle). RVU120 will be administered from day 1 to day 13 (total of 7 doses per cycle).	CDK8/19 - RVU120	II	Not yet recruiting	NCT06243458		

The efficacy of this combination was confirmed in the double-blind Phase III trial (PALOMA-2), where a 23-month follow-up showed a median PFS of 24.8 months in the palbociclib plus letrozole group versus 14.5 months in the letrozole-only group.^650^ Subsequently, the PALOMA-3 trial included both pre- and postmenopausal patients who had progressed or relapsed on prior endocrine therapy and could have had one line of chemotherapy in advanced disease settings. The results, again, revealed a notable benefit of the combination treatment (palbociclib plus fulvestrant) compared to fulvestrant alone, with a PFS of 9.5 versus 4.6 months, as per the final analysis.^651,652^

In the Phase II MONARCH-1 study, abemaciclib was tested as a single agent in previously heavily treated HR+/HER2- metastatic breast cancer patients, showing tolerable toxicity and promising clinical activity.^653^ The final analysis of MONARCH-1 was consistent with the initial report, with a median overall survival (OS) of 22.3 months, confirming the long-term efficacy of abemaciclib.^654^ Interim results of MONARCH-3, a phase III randomized, double-blind trial, showed that the combination of abemaciclib with nonsteroidal aromatase inhibitors (AI) in first-line setting significantly improved both PFS (median PFS not reached in the abemaciclib plus AI group vs. 14.7 months in the AI group) and objective response rate (59% in the abemaciclib plus AI group vs. 44% in the AI group) in postmenopausal HR+/ HER2-, chemotherapy-naive, advanced breast cancer patients.^655^ The final overall survival results, with a median follow-up of 8.1 years, showed that women treated with abemaciclib and AI had a median OS of more than 5.5 years, with an increase of 13.1 months compared to the control arm in the intent-to-treat population (66.8 vs. 53.7 months), although statistical significance for the OS outcome was not reached (hazard ratio 0.804; *P* = 0.06).^656^

The MONARCH-2 phase III trial combined abemaciclib with fulvestrant in chemotherapy-naive patients (any menopausal stage) who had relapsed on previous endocrine therapy. Results showed that the addition of abemaciclib to fulvestrant significantly improved both PFS (16.4 vs. 9.3 months) and ORR (48.1% vs. 21.3%).^657^ A recent update with long-term follow-up indicates that the addition of abemaciclib prolongs OS and chemotherapy-free survival, with the benefit being more pronounced in patients with a poor prognosis.^658^

In a similar setting, the efficacy of ribociclib in overcoming or delaying resistance to endocrine therapy was documented in the MONALEESA-2 phase III double-blind, placebo-controlled randomized trial.^659^ The updated analysis showed a significant benefit of combining ribociclib with letrozole, with a median PFS of 25.3 months for the ribociclib plus letrozole group versus 16.0 months for the letrozole group. At the time of this analysis, OS data was immature, but recently published OS data shows that the combination significantly improves OS, with a median OS more than a year longer in the ribociclib arm compared to the placebo arm.^660^

The MONALEESA-3 phase III trial showed that the combination of ribociclib with fulvestrant is equally effective in prolonging PFS (median PFS of 20.5 months for the ribociclib plus fulvestrant arm vs. 12.8 months for the fulvestrant arm) in postmenopausal women with HR+/HER2- advanced breast cancer.^661^ The most recent update corroborates the significant benefit of ribociclib plus fulvestrant in OS as well.^662^

The last dual CDK4/6 inhibitor to be approved for breast cancer treatment, at least from the Chinese NMPA, was dalpiciclib. Dalpiciclib was tested in the DAWNA-1 multicentric, randomized, double-blind, placebo-controlled, phase III trial in combination with fulvestrant in HR+/HER2- advanced breast cancer patients with disease progression after ET. The study showed that PFS was significantly prolonged with dalpiciclib plus fulvestrant (15.7 months) versus placebo plus fulvestrant (7.2 months), providing a significant benefit for patients who relapse or progress on previous ET.^663^ Following these promising results, the efficacy of AI (letrozole or anastrozole) plus dalpiciclib or placebo for endocrine-sensitive HR+/HER2- advanced breast cancer patients was evaluated in the DAWNA-2 trial. The study reported a median PFS longer in the dalpiciclib group than in the placebo group (30.6 months in the combination arm versus 18.2 months in the placebo arm, *P* < 0,0001), along with a substantial 49% reduction in the risk of disease progression or mortality. Notably, this suggest that dalpiciclib plus letrozole or anastrozole could be a novel standard first-line treatment for patients with HR+/HER2- advanced breast cancer.^664^

Still under clinical studies is lerociclib, which efficacy is being evaluated by LEONARDA-1 phase III trial, assessing the effect of fulvestrant plus lerociclib or placebo in patients who had experienced relapse or progression on prior ET. During the 2023 ASCO Annual Meeting were presented the results demonstrating a significantly prolonged PFS in the lerociclib group compared to the placebo group (11.07 months for lerociclib plus fulvestrant versus 5.49 months for placebo plus Fulvestrant, *p* < 0.001). The efficacy remained consistent across all subgroups, including those with primary endocrine resistance, showing a significantly reduced risk of disease progression or death with lerociclib (HR = 0.374, 95% CI: 0.182–0.769).^634^

In all abovementioned trials large portion of the enrolled patients are postmenopausal. In this regard, the phase III MONALEESA-7 study is unique in that it specifically tested the efficacy of ribociclib plus endocrine therapy in premenopausal HR+/HER2- advanced breast cancer patients. The addition of ribociclib to endocrine therapy proved significantly beneficial in extending PFS (23.8 months in the combination arm vs. 13.0 months in the placebo arm, *p* < 0.0001).^665^ The interim OS analysis at 42 months revealed significant long-term overall survival, with 70.2% in the ribociclib plus endocrine-treated group versus 46% in the endocrine-treated group.^666^

##### CDK4/6 inhibitors as adjuvant and neo-adjuvant therapies in breast cancer

In the adjuvant setting, the benefits of adding palbociclib to endocrine therapy have been evaluated in large Phase III trials (PALLAS and PENELOPE-B) involving HR+/HER2- breast cancer cohorts. The PALLAS trial included 5,760 participants with stage II/III breast cancer who had undergone surgery and adjuvant/neoadjuvant chemotherapy. The PENELOPE-B cohort was particularly enriched with high-risk patients, with one-third of the cohort having residual disease after neoadjuvant chemotherapy. The primary endpoint of these studies was invasive disease-free survival (iDFS). Both the interim (iDFS 88.2% in the palbociclib arm vs. 88.5% in the endocrine arm at 3 years) and final analyses (iDFS 84.2% in the palbociclib arm vs. 84.5% in the endocrine arm at 4 years) of the PALLAS study showed no benefit of palbociclib in the adjuvant setting.^667,668^ Equally disappointing results were reported in the PENELOPE-B study.^669^

The Phase III NATALEE study enrolled 5101 patients at any menopausal stage with stage > IIA HR+/HER2+ early breast cancer to evaluate the efficacy of ribociclib in combination with endocrine therapy over a 3-year treatment period. Preliminary analysis shows that the trial met its primary endpoint, with a 3.3% absolute benefit in iDFS for ribociclib plus endocrine therapy (iDFS 90.4% vs. 87.1%).^670^

The efficacy of abemaciclib in combination with endocrine therapy was evaluated in the MONARCH-E Phase III trial, with the primary endpoint being iDFS. The study enrolled 5637 patients and defined a high-risk group as those with either pathological axillary lymph nodes >= 4 or up to 4 pathological axillary nodes along with one of the following characteristics: 1) tumor size >= 5 cm, 2) grade >= 3, or 3) more than 20% Ki67 positivity. Unlike Palbociclib, treatment with abemaciclib plus standard endocrine therapy for two years significantly improved iDFS to 92.2% vs. 88.7%. Additionally, this interim analysis revealed that in premenopausal women, the addition of abemaciclib reduced the risk of developing iDFS by 37%.^671^ A recent update of the MONARCH-E study reassured the benefit of abemaciclib plus endocrine therapy and showed that the benefit is durable even after the completion of treatment, with a 4-year iDFS difference of 6.4% in favor of the abemaciclib plus endocrine-treated group.^672^ Based on this trial, the adjuvant abemaciclib plus endocrine therapy received FDA approval in 2021 for high-risk (Ki67 positivity > 20%, node-positive) HR+/HER2- early breast cancer patients. An updated analysis found that the benefit of abemaciclib is not correlated with a Ki67 score > 20%,^672^ leading to the removal of the Ki67 positivity requirement in the updated approval announcement in 2023.

In the neoadjuvant setting, the combination of palbociclib plus letrozole was compared to chemotherapy in the Neopal Phase II trial with 106 patients with HR+/HER- node-positive, high-risk advanced luminal A/B breast cancer, as defined by the PAM50 gene panel. The primary endpoint was residual cancer burden (RCB, 0-1 scoring), with secondary endpoints including clinical response, safety, and proliferation markers.^673^ The interim report was negative for the primary endpoint. However, secondary endpoint analysis showed that the Palbociclib-letrozole combination was as effective as chemotherapy in reducing Ki67 levels (geometric mean 1.17% and 3.3%, respectively). The most recent update in this trial validated previous observations, as there was no difference between palbociclib plus letrozole treatment and chemotherapy treatment (PFS 86.7% and 89.9%, respectively).^674^

Ribociclib in the neoadjuvant setting was studied in the CORALLEEN Phase II trial with 106 postmenopausal HR+/HER2- early-stage breast cancer patients, mostly luminal B. The primary endpoint was downstaging the risk of recurrence. At enrollment, 44 out of 52 patients in the ribociclib plus letrozole group and 48 out of 54 in the chemotherapy group were at high risk of recurrence. At surgery, 23 of 49 patients in the ribociclib plus letrozole group and 24 of 52 patients in the chemotherapy group were designated as low risk of recurrence.^675^ Although ribociclib plus endocrine therapy reduced the risk of recurrence, it was not superior to chemotherapy.^675^ Similarly, the FELINE Phase II study evaluated the addition of ribociclib to endocrine therapy in HR+/HER2- postmenopausal women. Although ribociclib (continuous or intermittent dosing) plus letrozole initially achieved significant CCCA (complete cell cycle arrest) compared to letrozole alone (at day 14, 92% vs. 52% respectively), proliferation rebounded in a significant number of patients in the combination arm by the time of surgery (63.3% vs. 71.4%).^676^

The PALLET Phase II study evaluated the neoadjuvant combination of palbociclib and letrozole in postmenopausal HR+/HER2- early breast cancer patients. Patient inclusion criteria included unilateral non-metastatic tumors measuring >= 2 cm by ultrasound. The addition of Palbociclib significantly reduced proliferation (Ki67) and CCCA. However, the clinical response between groups did not reach statistical significance.^677^ The NeopalAna study, a single-arm Phase II study in 50 HR+/HER2- breast cancer patients, evaluated the antiproliferative effect (complete cell cycle arrest, CCCA, Ki67 <= 2.7%) of sequentially administered endocrine and palbociclib treatment as the primary endpoint.^678^ This study demonstrated that the addition of palbociclib imposes an antiproliferative effect regardless of menopausal state, luminal subtype, histology, stage, grade, PI3K, P53, PTEN, and RUNX1 mutational status. Moreover, the study highlights the importance of continuing palbociclib treatment, as early palbociclib withdrawal led to a Ki67 rebound after surgery.^678^ In line with the NeopalAna study, the DxCARTES Phase II trial results also showed that palbociclib plus letrozole-driven downstaging is independent of the tumor baseline molecular characteristics.^679^

The NeoMONARCH open-label Phase II study randomized 224 postmenopausal stage I-IIIB HR+/HER2- breast cancer patients to two weeks of combination (abemaciclib plus anastrozole), abemaciclib and anastrozole monotherapies, followed by 14 weeks of combination treatment. The primary endpoint was to evaluate changes in Ki67 at the end of the run-up treatment.^680^ The study met its primary endpoint, with a reduction in Ki67 (geometric mean change of -93%, -91%, and -61% for the combination group, abemaciclib monotherapy group, and anastrozole monotherapy group, respectively). As a result, a significantly higher number of patients achieved CCCA in the abemaciclib-treated groups compared to the anastrozole monotherapy group.^680^ Further studies and molecular characterizations performed with specimens from this trial revealed several noteworthy results. First, early withdrawal of CDK4/6 inhibitors led to a Ki67 rebound, which was also reported in the NeopalAna study. Second, transcriptome analysis revealed activation of immune-related pathways only in the combination arm. However, histopathological analysis did not find any change in Tumor Infiltrating Lymphocytes (TIL).^681^ In a preclinical model, it was shown that CDK4/6 inhibition leads to increased antigen presentation on tumor cells and imposes an antiproliferative effect on Treg cells, thus activating an immune response. Therefore, CDK4/6 inhibitors may activate the immune response without increasing the number of TILs. The study by Scirocchi et al., specifically designed to test the effect of CDK4/6 inhibition on immune response in HR+/HER2- metastatic breast cancer, showed that at least a month-long CDK4/6 inhibition plus endocrine therapy depletes circulating Treg cells and Myeloid-derived suppressor cells (MDSC), while increasing naive CD3+ and effector CD8+CD137+T cells.^682^

In this regard, combinations of CDK4/6 inhibition with immune checkpoint inhibitors have been evaluated in early clinical trials. A phase Ib study by Rugo et al. investigated the combination of abemaciclib plus pembrolizumab in HR+/HER2- metastatic breast cancer, but deemed the combination to be highly toxic.^683^ Another phase I/II study assessed the combination of palbociclib, letrozole, and pembrolizumab. Although some clinical activity was noted (31% complete response, 25% partial response, and 31% stable disease), grade III/IV toxicities were common.^684^ Nevertheless, initial reports from the PACE trial are encouraging, as administration of avelumab in patients who progressed on initial CDK4/6 inhibition doubled PFS time.^685^

##### CDK4/6 inhibitors in other breast cancer types

Due to the success of CDK4/6 inhibitors in HR+/HER2- breast cancer therapy, their efficacy in other breast cancer subtypes is being tested.

The NA-PHER exploratory Phase II trial evaluated the combination of palbociclib, fulvestrant, and pertuzumab in 30 treatment-naive patients with HR+/HER2+ unilateral invasive breast cancer. The primary endpoint was to assess changes in Ki67 levels at week 2 and during surgery, with secondary endpoints including clinical objective responses and pathological complete responses. The interim report demonstrated that the triple-targeting regimen significantly reduced Ki67 levels both at two weeks and at the time of surgery. Additionally, 29 out of 30 patients achieved a clinical objective response, and 8% achieved a pathological complete response at surgery.^686,687^ Currently ongoing, this trial, if confirmed in larger cohorts with placebo groups, may pave the way for a chemotherapy-free option.

The PATRICIA Phase II trial tested the combination of palbociclib plus trastuzumab with or without letrozole in advanced HER2+ breast cancer patients who had received at least one prior line of trastuzumab therapy. Initial analysis indicated that ER+ subgroups, especially those treated with the triple combination, achieved longer progression-free survival (PFS) at 6 months. The study also underscored the potential of the PAM50 gene panel as a biomarker, with transcriptomic profiling revealing that luminal subtype tumors benefited significantly from palbociclib treatment in terms of PFS.^688^

The ongoing Phase III PATINA trial, expected to conclude in 2026, is investigating the addition of Palbociclib to trastuzumab or pertuzumab plus endocrine therapy in HR+/HER2+ metastatic breast cancer settings.^689^

In the MONARCH-HER Phase II study, triplet therapy with abemaciclib, fulvestrant, and trastuzumab prolonged progression-free survival (PFS) (8.3 months in the triplet arm vs. 5.7 months in chemotherapy plus trastuzumab).^690^ A recent update on overall survival (OS) also showed a numerical extension in OS time in the triplet treatment arm. Exploratory analysis of the study indicated that the triplet regimen was more effective in luminal subtype tumors compared to non-luminal types, in terms of both PFS and OS.^691^

CDK4/6 inhibitors were tested also in patients with triple negative breast cancer. Indeed, Trilaciclib (approved in 2021 by FDA to decrease chemotherapy-induced myelosuppression in patients with extensive-stage small cell lung cancer) was evaluated in an exploratory, randomized, open-label, phase II trial that investigated the efficacy and safety of administering trilaciclib prior to gemcitabine in patients with TNBC who had previously received up to two lines of therapy in the metastatic setting. Results showed that administration of trilaciclib prior to gemcitabine resulted in longer PFS (median 9.4 months vs. median 5.7 months) and significantly longer OS compared with GCb alone (median 20.1 months vs. 12.6 months).^692^ The phase III of PRESERVE 2 trial has been designed to confirm the survival benefit observed in the phase II study of trilaciclib in patients with metastatic TNBC.^693^ The study was completed in May 2024 and the results are not published yet.

##### CDK4/6 inhibitors in other tumors

Early studies have explored the efficacy of CDK4/6 inhibitors beyond breast cancer, extending into other solid and hematological malignancies. For instance, Patnaik’s phase I trial in diverse solid tumors highlighted the potential of CDK4/6 inhibition as a rational strategy, particularly in KRAS-mutated non-small cell lung cancer (NSCLC) (disease control rate 55% vs. 39% in KRAS Mut vs. WT patients), as well as in melanoma patients harboring NRAS mutations.^694^ A subsequent JUNIPER phase III trial compared abemaciclib with erlotinib in previously treated, KRAS-mutated NSCLC patients. While the study did not meet its primary endpoint of median overall survival (7.4 vs. 7.8 months), abemaciclib demonstrated superiority in secondary endpoints such as median progression-free survival (3.6 vs. 1.9 months) and objective response rate (8.9% vs. 2.7%), warranting further investigation.^695^

Several early-phase trials targeting CDK4/6 in KRAS-mutated tumors have shown promising results. Moreover, Puyol et al. elegantly demonstrated a synthetic lethal interaction between CDK4 and KRAS in mouse models.^696^

Historically, the “undruggable” nature of KRAS due to its intrinsic structural features limited the possibility of co-targeting CDK4/6 and KRAS. However, recent successes in developing KRAS inhibitors represent a significant milestone in cancer research, potentially revolutionizing treatment for KRAS-driven malignancies such as pancreatic, non-small cell lung, and colon cancers.^697,698^ Co-targeting CDK4/6 and KRAS in these settings holds promise for improving clinical outcomes. The Phase I KRYSTAL-16 study is currently evaluating the combination of CDK4/6 inhibitors and the KRAS inhibitor adagrasib in solid tumors harboring KRAS G12C mutation, aiming to further explore this therapeutic approach.^699^

In human papillomavirus (HPV)-negative, platinum- and/or cetuximab-resistant head and neck squamous cell carcinomas (HNSCC), initial studies combining palbociclib with cetuximab showed promising results. However, in the larger PALATINUS phase II double-blinded randomized study, palbociclib plus cetuximab did not significantly improve overall survival (OS) or progression-free survival (PFS) in cetuximab-naive HPV-negative HNSCC patients.^700^ Biomarker analysis within the PALATINUS study identified subgroups of patients (e.g., CDK4/6 amplification, cyclin D1 expression < 80% by immunohistochemistry, CDKN2A mutations in cfDNA) potentially benefiting from CDK4/6 inhibitors, despite the limitation of a small study population.^700^ These findings underscore the importance of testing CDK4/6 inhibitors in well-selected populations in larger studies.

It is noteworthy that the introduction of immune checkpoint inhibitors (ICI) has reshaped the treatment landscape of HNSCC.^701–704^ Inhibition of CDK4/6 has been shown to trigger immune responses, particularly by increasing antigen presentation in tumor cells and depleting the regulatory T cell (Treg) repertoire.^681,682^ Building on this knowledge, a recent phase I study evaluated the combination of palbociclib, cetuximab, and avelumab in advanced HNSCC, demonstrating that this triplet treatment is safe and exhibits promising antitumor activity (ORR 42%).^705^

Mantle Cell Lymphoma (MCL), characterized by overexpression of cyclin D1, presents a potential vulnerability to CDK4/6 targeted therapies.^706^ Early-phase clinical trials have assessed the efficacy of CDK4/6 inhibitors in this context, showing initial promising activity.^707^ In a phase I study, combination therapy of CDK4/6 inhibition with the Bruton Tyrosine Kinase (BTK) inhibitor ibrutinib extended duration of response, with an objective response rate (ORR) of 67%, similar to that of single-agent ibrutinib.^708^ Ongoing Phase II trials (NCT02159755) are expected to further elucidate the role of CDK4/6 inhibitors in MCL, with results anticipated in 2025.

A complete list of interventional clinical trials recruiting, active not recruiting and not yet recruiting using CDKs inhibitors is provided as supplementary table one and obtained by searching the https://www.clinicaltrials.gov/ database on may 2024. Among 119 listed trials the vast majority is testing different CDK4/6 inhibitors in different type of cancer alone or in combination therapies (Table S1). Among the few other CDKs inhibitors being tested we outline here the CDK9 inhibitor Voruciclib in a Phase I trial in subjects with relapsed and/or refractory B cell malignancies or AML after failure of prior standard therapies alone or in combination with venetoclax (NCT03547115). In similar settings is being tested the CDK9 inhibitor GFH009 in the Phase I/II NCT04588922 trial. The Phase I/II NCT04983810 trial is recruiting patients with advanced solid tumors and lymphoma investigate the safety, pharmacokinetics, and efficacy of the oral CDK2/9 inhibitor, fadraciclib (CYC065).

The CDK7 inhibitor Q901 is being tested in a phase 1/2 multicenter, open-label, dose-escalation, safety, pharmacodynamic, and pharmacokinetic study (NCT05394103) in adult patients with selected advanced solid tumors. Same settings and design are using to test the CDK2 inhibitor INX-315 (NCT05735080).

Not yet recruiting is the NCT06243458 trial that will evaluate the orally administered Cyclin-Dependent Kinase (CDK) 8/19 inhibitor RVU120, in terms of erythroid hematologic improvement and safety in participants with lower-risk myelodysplastic syndrome (MDS), opening the way to the clinical study of a more specific inhibition of transcriptional CDKs.

### Key takeaways

The first aspect that has emerged clearly from this large work of literature revision, is how the complexity of this topic increases exponentially with the accumulation of knowledge. Every step forward in biomedical research is inextricably linked to the development of other sciences, such as chemistry, mathematics and physics. Today we are faced with an unprecedented development of computer science and omics that allows the dissection of each normal or pathological tissue from both molecular and spatial points of view. We need to integrate the knowledges and the collected information in ways that can be understandable and of potential clinical use. Which part of the accumulated information is of primary interest, toward the development of novel diagnostic, prognostic and therapeutically useful information? Can we rely on the use of artificial intelligence approaches to summarize the accumulating knowledge? We strongly believe that most promising results will need extensive validation in appropriate models. Increasingly in-depth studies, characterizing human cells and tissues, need a biological and functional validation. Vice versa, observations made in vitro and in preclinical models need to be validated in human samples. In both cases, it is important that this approach will be carried out in the context of multidisciplinary and multiprofessional research groups, with competences in biological, medical, physical and mathematical sciences.

The use of simple models is necessary in biology and medicine and the study of cell cycle and CDKs is a striking example. The initial use of yeast allowed the identification of the first CDK. The use of genetically modified mouse models then permitted the better understanding of the role of individual CDKs in mouse and human physiological and pathological conditions, like cancer. Now we need to develop better models that recapitulate to a greater extent the complexity of human pathologies. These include of course patients derived- and genetically modified-models, but also computer designed virtual patients that rely on the accumulated physio-pathological notions, which could help in better define the heterogeneity of human diseases. For instance, in cancer research, sporadic tumors arise from the accumulation of mutations in single somatic cells or clones. Generating realistic, reliable and reproducible somatic models of cell transformation will be mandatory to understand how cancer cell clones really progress in the context of an otherwise healthy tissue.

Complexity is further enhanced by CDKs overlapping functions. Although we can schematically divide CDKs in three main subfamilies (i.e. cell cycle, transcriptional and atypical) each CDK has multiple functions that go beyond the ones ascribed to its subfamily. These functions could vary depending on sub cellular localization, transcriptional or post-transcriptional modification and binding to different cyclins or regulatory subunits. These multiple layers of complexity should be carefully taken into account when the inhibition of one or more CDKs is explored for therapeutic purposes.

On the other hand, most CDKs remain understudied and their roles in embryogenesis, adult tissue physiology and human pathology are largely unknown. Gaining a deeper understanding of these lesser-known CDKs could reveal their potential as therapeutic targets. Cancer research studies have shown us that inhibiting multiple CDKs often leads to unacceptable toxicities. Therefore, selectively inhibiting individual CDKs in specific pathological contexts may be a more effective and safer strategy for treating chronic diseases. For instance, in Alzheimer’s disease models, inhibition of CDK5 alleviate cognitive defects.^476^ Novel approaches, such as specific protein degraders, or computationally designed small-molecule inhibitors, could help determine whether targeting CDK5 could delay or prevent the progression of such invalidating disease, without causing significant toxicity.

At this regard, it is important to note that kinase independent effects have been described for many CDKs, including regulation of transcription and differentiation.^52^ These observations implicate that chemical and genetic inhibition (or targeted protein degradation) could have different effects in term of both disease control and toxic effects. The use of kinase dead mutant models along with tissue specific expression could be useful to better characterize the role(s) of understudied kinases and the still unclear role(s) of the most explored ones.

Despite the extensive testing of several CDK inhibitors across different cancer types, only CDK4/6 inhibitors have been approved for clinical use, specifically in breast cancer patients. This observation calls for a profound rethinking of our approach to clinical research, not just in cancer but in other diseases as well.

One key lesson learned is that targeting oncogenic drivers significantly increases the likelihood of achieving clinically meaningful results. Cyclin D/CDK4/6 complexes are crucial in cancer progression, particularly in breast cancer, as revealed by study of human pathology and preclinical models. Another important insight is that inhibiting specific kinases is more effective and manageable in terms of toxicity control and clinical utility than broad inhibition of all or most cell cycle CDKs, as emerged from the studies of first- and second-generation inhibitors. Lastly, identifying a compelling target is not enough if you lack a very specific way to inhibit its activity. Notably, among CDK4/6 inhibitors in clinical use, those more selective for CDK4, such as ribociclib and abemaciclib,^577^ seem to be more effective in the clinical setting than those inhibiting both CDK4 and CDK6, like palbociclib. This observation is in line with the notion that cyclin D1/CDK4 complex is particularly implicated in breast cancer progression among the various cyclin D/CDK4/6 complexes. Yet, it is also possible that the superior efficacy of ribociclib and abemaciclib could be linked to still undiscovered off target effects.

Another critical point to highlight, for a successful application of targeted anti-CDK therapies, is the definition of the appropriate population of patients to be selected, the stage of the disease in which the therapy is likely more active and the combination therapy that should be used, if any. The highest benefit in treatment with CDK4/6 inhibitors was achieved from patients with advanced HR+/HER2- breast cancer, in combination with endocrine therapies. The expression of hormone receptors and the lack of HER2 amplification were clear predictive biomarker of response and were used to select the patient population. Also, it was clear that CDK4/6 inhibitors worked better when administered in combination with endocrine therapies. However, moving to a very similar patient population, only with an early-stage disease, the same was less or not true. This observation could have many different molecular explanations. First, it could be easier to observe treatment benefits when the standard therapy (in this case endocrine therapy) is less active, compared to a situation in which the disease is still very hormone-driven. Secondly, in advanced setting, breast cancer may have accumulated more genomic alterations that render tumor cells more sensitive to CDK4/6 inhibition. Finally, it is worth to mention that the effects of CDK4/6 inhibition on the patients’ immune system and tumor metabolism could be quite different in these two different stages of diseases.

Many valuable lessons were also learned from the unsuccessful clinical experiences. First, the substantial failure of broad-range inhibitors in cancer patients, due to both high toxicity and limited clinical benefits, suggests that this approach should be either abandoned or critically re-evaluated. Whether the lack of success was due to poor patients’ selection is something that remains unclear. At this regard, roscovitine (seliciclib), a broad inhibitor of CDK1, CDK2, CDK7 and CDK9, showed promising effects in Cushing disease.^601^ In this case, the strong preclinical evidence supporting CDK2 hyper-activation as a key driver in pituitary corticotrope cell adenoma provides a rationale for its testing, managing the observed side effects through dose adjustments. This raises the possibility that, in non-cancer disease characterized by hyperproliferation, the use of CDK inhibitors at lower doses or on an intermittent schedule, could provide clinical benefit for selected patients, with tolerable side effects. Of course, strong preclinical evidence supporting the involvement of CDKs in the disease pathogenesis would be essential.

Beyond CDK4/6 inhibitors, very few other CDK inhibitors are currently being tested in cancer, and none in other diseases. This lack of investment in clinical research is likely a consequence of the profit-driven nature of drug development research. It is understandable that pharmaceutical and biotech companies prioritize compounds with a high likelihood of clinical success. Yet, this approach has led to a narrow focus, with CDK4/6 inhibitors being repeatedly tested in the same patient population, while other diseases remain orphan of active treatments. What is even more puzzling is why the public stakeholders have not adequately fund drug development from preclinical studies to clinical application. The COVID-19 pandemic demonstrated that when public and private resources are combined, as they were for the development, testing, and production of the anti-SARS-CoV-2 vaccines, significant progress can be made. A similar collaborative approach could potentially accelerate the development of CDK inhibitors for a broader range of diseases.

### Conclusions and perspectives

Since their discovery, CDKs have been recognized as key regulators of cellular processes, including cell proliferation, gene transcription, translation, and RNA metabolism. Consequently, CDK inhibition has been explored as a therapeutic strategy for several diseases, particularly cancer. The greatest success has come from inhibiting cell cycle CDKs, especially CDK4 and CDK6, in certain cancer types. However, given their involvement in various pathologies, CDKs are also potential targets for neurodegenerative, metabolic, cardiovascular, and autoimmune diseases.

Emerging non-canonical roles of CDK4 and CDK6 could be leveraged to counteract drug resistance in cancer treatment, improving patient outcomes. The approval of CDK4/6 inhibitors has spurred interest in next-generation of CDK2 inhibitors, which show potential in cancer therapy. Additionally, very recent findings, published while this manuscript was under review, suggest that cyclin B1-CDK5 plays a role in ensuring mitotic fidelity,^709^ highlighting the potential of CDK5 as a cancer therapeutic target. This opportunity should be considered when exploring mitosis as a potential target, even because the direct inhibition of CDK1, master regulator of M phase progression, is likely impractical due to the widespread toxicity associated with its inhibition. CDK5 can act as both an oncogene and a tumor suppressor, in different cancer types. It regulates processes like epithelial-to-mesenchymal transition, protein degradation, mitochondrial apoptosis, and cytoskeletal remodeling. Its levels, along with those of its cofactors, have been linked to prognosis across various cancers, further supporting the idea that it could also represent a valid therapeutic target.

From a molecular and biological point of view, the use of novel and more precise and sophisticate approaches like chemical genetics, might help to discover new functions of the different CDKs, belonging to the diverse CDK subfamilies.^709^ Starting from here, we could expect that with improvement in the technologies to modulate the expression and functions of specific CDKs in living cells and organisms, in the next years we will be able to better dissect the complex role(s) of the still understudied CDKs. We could predict that exploring better the role of transcriptional and atypical CDKs will highlight previously undisclosed regulatory pathways that participate to the onset and progression of human diseases. The description and clarification of these molecular mechanisms could then provide the way to better personalize the treatments for patients.

Challenges remain in targeting CDKs for non-cancer diseases, where off-target effects and lack of selective inhibitors severely complicate treatment. Advances in pharmaceutical chemistry and development of protein degraders are expected to improve CDK specificity and reduce toxicity.^710,711^

Looking ahead, transcriptional CDK inhibitors hold promise for treating diseases with transcriptional dependence. However, challenges in developing selective inhibitors persist, due to the structural similarities between CDKs and other kinases, as seen with CDK12 and CDK13. Artificial Intelligence-driven drug design and synergistic drug combinations may enhance selectivity and therapeutic outcomes, especially in context like drug-resistant cancers, in which current treatments are not sufficient.

Among transcriptional CDKs, CDK9 and other transcription-associated CDKs, like CDK11, are also emerging as important targets. For instance, cancer cells addicted to continuous protein production are particularly vulnerable to CDK9 inhibition.^177^ On the other hand, CDK9 inhibition represents a potential therapeutic strategy against chemotherapy-persistent tumor cells for its ability to interfere with diapause-like adaptation, necessary for persistent cells to survive the pressure of chemotherapy.^712^ However, careful dosing and treatment schedules are needed to manage toxicities and optimize therapeutic windows.

Another aspect that should be taken into serious consideration is the multiple roles of the different CDKs. For instance, CDK5 plays a prominent role in neural development and in neurodegenerative diseases, like Alzheimer’s, Parkinson’s, and ALS. Its involvement in neuron migration and differentiation, as well as its accumulation in the brain of affected patients, make it a promising target for halting neurodegeneration. This also means that targeting CDK5 for cancer therapy might elicit unwanted neurological side effects. How to manage these multiple activities represents one of the most challenging issues to address in the next future.

CDKs are also involved in inflammatory conditions, contributing to immune cell proliferation, cytokine production, and inflammation-related diseases. Though CDK inhibitors show some potential in these settings, progress has been slow, due to the lack of selective and safe inhibitors. Continued research is required to cover this therapeutic gap. At this regard, it is important to highlight that while cancer is a highly and rapidly progressing disease that need to be treated and defeated in a relatively short period of time to prevent patients’ death, these other diseases are chronic conditions that could last very long and with which the patients could, even with difficulty, cohabit. As a consequence, the balance between therapeutic and side effects of any drug need to be very seriously considered and, therefore, CDKs have a long way ahead before being explored as therapeutic targets in these settings, unless specific understudied CDKs will demonstrate a pivotal role in their pathogenesis.

In cancer therapy, CDK inhibitors have revolutionized treatments, especially in advanced breast cancer, with the use of CDK4/6 inhibitors. However, reducing toxicity, identifying biomarkers of response, and addressing drug resistance remain critical challenges. New resistance mechanisms to CDK4/6 inhibitors, such as RB loss and activation of the PI3K/AKT/mTOR pathway, emphasize the need for further research. Next-generation CDK inhibitors, specifically targeting CDK4 and CDK2, are showing promise in overcoming resistance and improving patient outcomes. For instance, CDK4-selective inhibitors that minimize CDK6 blockade, might reduce toxicity and enhance efficacy. Yet, as mentioned above, even the most specific inhibitor or degrader agent might have side effects due to the multiple roles played by each individual CDK.

In the upcoming years, the multifaced role of individual CDKs and how their activity could be specifically modulated to cure human diseases will certainly be better dissected. In cancer and, likely, in neurodegenerative diseases, new specific CDK inhibitors will be tested and, hopefully, approved. This progress will be possible thanks to the technological advancements that, combined with rigorous functional and preclinical experimentations, will provide a clearer picture of how and when to use a specific CDK as a therapeutic target in human disease.

## Supplementary information

Supplementary Table S1
