# Supplementary material for: Cyclin-dependent protein kinases and cell cycle regulation in biology and disease
Source: Signal Transduct Target Ther. 2025 Jan 13;10:11. doi: 10.1038/s41392-024-02080-z (PMC11734941; doi:10.1038/s41392-024-02080-z)
Supplement: Supplementary file 1 — Supplementary Table S1 [file 41392_2024_2080_MOESM1_ESM.docx]

Supplementary Materials for

Cyclin-dependent protein kinases and cell cycle regulation in biology and disease

Ilenia Pellarin, Alessandra Dall’Acqua, Andrea Favero, Ilenia Segatto, Valentina Rossi, Nicole Crestan, Javad Karimbayli, Barbara Belletti and Gustavo Baldassarre.

Correspondence to: [gbaldassarre@cro.it](mailto:gbaldassarre@cro.it)

**This PDF file includes:**

Tables S1

**Table S1. Ongoing interventional clinical trials using CDKs inhibitors**

| **#** | **Study Name** | **Intervention** | **CDK - Inhibitor** | **Phase** | **Status** | **NCT #** |
| --- | --- | --- | --- | --- | --- | --- |
| **1** | Phase I Study of the CDK4/6 Inhibitor Palbociclib (PD-0332991) in Combination With the PI3K/mTOR Inhibitor Gedatolisib (PF-05212384) for pts With Advanced Squamous Cell Lung, Pancreatic, Head & Neck and Other Solid Tumors | **Arm A.** Palbociclib (orally, daily) on Days 1-21 for each of the 4-week cycles at a pre-determined dose. Gedatolisib (intravenously, weekly) on the first day for each of the four weeks during the 4-week cycles at a pre-determined dose. | CDK4/6-Palbociclib | I | Recruiting | NCT03065062 |
| **2** | Cyclin Dependant Kinase 4/6 (CDK4/6) Inhibitors as a Second Line Treatment in Metastatic Breast Cancer pts | **Arm A.** Palbociclib 125 mg tab/day for 3 weeks and 1 week rest + Fulvestrant 500 mg intrmuscular injection on day 1, 15, 29 of the 1st cycle, then every month. Arm B: Ribociclib 200 mg 3 tabs/day for 3 weeks and 1 week rest+ Fulvestrant 500 mg intramuscular injection on day 1, 15, 29 of 1st cycle, then every month | CDK4/6-Palbociclib/ Ribociclib | III | Recruiting | NCT05670054 |
| **3** | Implementing Geriatric Assessment for Dose Optimization of CDK 4/6-inhibitors in Older Breast Cancer pts - a Pragmatic Randomized-controlled Trial (IMPORTANT Trial) | **Arm A.** -1 level dose reduction as initial dose of either one of the CDK 4/6-inhibitors: Palbociclib 100 mg x 1 for 21 days with 7 days off; or Ribociclib 400 mg x 1 for 21 days with 7 days off; or Abemaciclib 100 mg x 2 daily added to endocrine therapy. Arm B: Full initial dose of either one of the CDK 4/6-inhibitors: Palbociclib 125 mg x 1 for 21 days with 7 days off; or Ribociclib 600 mg x 1 for 21 days with 7 days off; or Abemaciclib 150 mg x 2 daily) added to physician's choice endocrine therapy. Arm C: Full initial dose of either one of the CDK 4/6-inhibitors: Palbociclib 125 mg x 1 for 21 days with 7 days off; or Ribociclib 600 mg x 1 for 21 days with 7 days off; or Abemaciclib 150 mg x 2 daily) added to physician's choice endocrine therapy. | CDK4/6-Palbociclib | III | Recruiting | NCT06044623 |
| **4** | Discontinuation of CDK4/6 Inhibitors in pts With Metastatic HR Positive, HER2 Negative Breast Cancer With Durable Disease Control: A Randomized Low-intervention Phase II Trial of the AIO Working Groups Breast Cancer and Quality of Life | **Arm A.** Continuation of CDK4/6 inhibitor treatment + Continuation of endocrine treatment. **Arm B.** Discontinuation of CDK4/6 inhibitor treatment + Continuation of endocrine treatment | CDK4/6-Palbociclib/ Abemaciclib | II | Recruiting | NCT06207734 |
| **5** | Prospective Evaluation of Determinants of Resistance to Endocrine Therapy and a Cyclin-dependent Kinases 4 and 6 (CDK4/6) Inhibitor in Hormone Receptor (HR) Positive Metastatic Breast Cancer (MBC) | **Arm A: Participants with untreated metastatic disease receiving ET and a CDK 4/6. Arm B: Participants initiating a CDK 4/6 i after progression on ET.** Participants will undergo blood collection (intervention) at time of initiating treatment with endocrine therapy and palbociclib, at 4 weeks after initiating this treatment, and every 3-4 months while on treatment. If a participant progresses on this treatment, they will have a blood collection at that time. | CDK4/6-Palbociclib | II | Recruiting | NCT03439735 |
| **6** | A Phase III Randomized, Open-Label Study Evaluating Efficacy and Safety of Giredestrant Compared With Fulvestrant, Both Combined With a CDK4/6 Inhibitor, in pts With Estrogen Receptor-Positive, HER2-Negative Advanced Breast Cancer With Resistance to Prior Adjuvant Endocrine Therapy | **Arm A: Giredestrant + Investigator's Choice of CDK4/6i.** Participants in the experimental arm will receive giredestrant plus the investigator's choice of CDK4/6 inhibitor (CDK4/6i): Palbociclib, ribociclib, or abemaciclib. **Arm B: Fulvestrant + Investigator's Choice of CDK4/6i.** Participants in the control arm will receive fulvestrant plus the investigator's choice of CDK4/6 inhibitor (CDK4/6i): Palbociclib, ribociclib, or abemaciclib. | CDK4/6-Palbociclib/ Abemaciclib / Ribociclib | III | Recruiting | NCT06065748 |
| **7** | A Phase Ib/II Study Confirmed Inhibition of Autophagy Synergizes Anti-tumor Effect of High Dose CDK4/6i With Manageable Safety in HR+HER2-breast Cancer pts | **Arm A.** 600mg bid dose of hydroxychloroquine group + with three predefined dose groups of Palbociclib (100mg QD, 150mg QD, and 200mg QD), separately. **Arm B.** After MTD was determined, RP2D dose was selected for phase II clinical trial. | CDK4/6-Palbociclib | I/II | Recruiting | NCT05953350 |
| **8** | A Phase III, Double-blind, Randomised Study to Assess Switching to AZD9833 (a Next Generation, Oral SERD) + CDK4/6 Inhibitor vs Continuing Aromatase Inhibitor (Letrozole or Anastrozole)+ CDK4/6 Inhibitor in HR+/HER2-MBC pts With Detectable ESR1Mutation Without Disease Progression During 1L Treatment With Aromatase Inhibitor+ CDK4/6 Inhibitor- A ctDNA Guided Early Switch Study | **Arm A.** The pts will receive AZD9833 (75 mg, PO, once daily) + Palbociclib (PO, once daily, 125, 100 or 75 mg for 21 consecutive days followed by 7 days off treatment), abemaciclib (PO, twice daily, 150,100 or 50 mg) or ribociclib (To Be Determined, PO, once daily for 21 consecutive days followed by 7 days off treatment) + anastrozole placebo (PO, once daily) or letrozole placebo (PO, once daily) **Arm B.** The pts will recieve anastrozole (1 mg, PO, once daily) or letrozole (2.5 mg, PO, once daily) + Palbociclib (PO, once daily, 125, 100 or 75 mg for 21 consecutive days followed by 7 days off treatment), abemaciclib (PO, twice daily, 150, 100 or 50 mg) or ribociclib (To Be Determined, PO, once daily for 21 consecutive days followed by 7 days off treatment) + AZD9833 placebo (PO, once daily) | CDK4/6-Palbociclib/ Abemaciclib / Ribociclib | III | Recruiting | NCT04964934 |
| **9** | A Randomized, Open-label, Parallel-group Study Comparing the Immune Modulation Effect of Ribociclib, Palbociclib, and Abemaciclib in Early ER+/HER2- Breast Cancer | **Arm A.** Palbociclib + Letrozole. **Arm B.** Ribociclib + Letrozole. **Arm C.** Abemaciclib + Letrozole. | CDK4/6-Palbociclib/ Abemaciclib / Ribociclib | II | Recruiting | NCT05766410 |
| **10** | Palbociclib Combine With Endocrine Therapy and Anti-HER2 Therapy in HR Positive and HER2 Positive Advanced Breast Cancer | **Arm A.** CDK4/6 inhibitor: 125mg, (oral QD) from the 1st to the 21st day of the 28 day cycle. Trastuzumab: on the first day of the 21 day cycle, the initial dose was 8mg/kg, and the intravenous infusion was 90 minutes; Every 3 weeks thereafter, the dose is 6mg/kg, and the intravenous infusion is 30~90 minutes. Pertuzumab: on the first day of the 21 day cycle, the initial dose was 840mg, intravenous infusion was 60 minutes, and then once every 3 weeks, the dose was 420mg, and the infusion time was 30-60 minute. Letrozole selected by the doctor: 2.5mg, oral Q24H or exemestane from the 1st to the 21st day of the 21 day cycle: 25mg, oral Q24H from the 1st to the 21st day of the 21 day cycle. The efficacy is evaluated every 2 months (CR, PR, SD, PD). | CDK4/6-Palbociclib | II | Recruiting | NCT05969184 |
| **11** | Multicenter, First-line Metastatic Open-label Prospective Phase II Trial Evaluating the Combination of Palbociclib (CDK 4/6 Inhibitor) and Hormone Therapy (Letrozole or Anastrozole) in Women With Luminal, HER2 Negative Advanced Breast Cancer: Evaluation of the Prediction of Individual Treatment Efficacy Using Infrared Laser Spectroscopy Analysis on Liquid Biopsies (Quantum Optics). | **Arm A.** Palbociclib (125 mg daily per os (3 weeks on-1 week off) with dose adaptation according to safety profile) + non-steroidal aromatase inhibitor (Letrozole (2.5mg) or Anastrozole (1mg) daily per os). This combination will continue until progression for an average duration of 2 years. | CDK4/6-Palbociclib | II | Recruiting | NCT05190094 |
| **12** | A Phase Ib/III, Open-label, Randomised Study of Capivasertib Plus CDK4/6 Inhibitors and Fulvestrant Versus CDK4/6 Inhibitors and Fulvestrant in Hormone Receptor-Positive and Human Epidermal Growth Factor Receptor 2-Negative Locally Advanced, Unresectable or Metastatic Breast Cancer (CAPItello-292) | **Arm A.** Capivasertib Plus Palbociclib and Fulvestrant (Ph 1b) **Arm B.** Capivasertib Plus Ribociclib and Fulvestrant (Ph 1b) **Arm C**. Capivasertib Plus Abemaciclib and Fulvestrant (Ph 1b) **Arm D.** Capivasertib Plus Fulvestrant and Investigator's choice of CDK4/6i (Palbociclib or ribociclib) (Ph III) **Arm E.** Fulvestrant and investigator's choice of CDK4/6i (Palbociclib or ribociclib) (Ph III) | CDK4/6-Palbociclib | III | Recruiting | NCT04862663 |
| **13** | Randomised Phase II Study of Induction Fulvestrant and CDK4/6 Inhibition With the Addition of Ipatasertib in Metastatic ER+/HER2- Breast Cancer pts Without ctDNA Suppression | **Arm A.** Where high ctDNA is detected in screening, pts to be randomised on a 1:1 basis to interventional arm or comparison arm. pts randomised to interventional arm receive Palbociclib + Fulvestrant + Ipatasertib. **Arm B.** Where high ctDNA is detected in screening, pts to be randomised on a 1:1 basis to interventional arm or comparison arm. pts randomised to Comparison arm receive Palbociclib + Fulvestrant. **Arm C.** Where no ctDNA is detected in screening, pts to be allocated to the observational arm and receive standard of care (Abemaciclib / Ribociclib / Palbociclib + fulvestrant). **Arm D.** Where low ctDNA is detected in screening, pts to be allocated to the observational arm and receive standard of care (Abemaciclib / Ribociclib / Palbociclib + fulvestrant). | CDK4/6-Palbociclib | II | Recruiting | NCT04920708 |
| **14** | Phase Ib Trial Evaluating the Combination of CDK4 Inhibitor With Immunotherapy in pts With Undifferentiated Pleomorphic Sarcoma (UPS) | **Arm A.** Palbociclib given for 2 weeks following a pre-treatment ultrasound guided biopsy used to establish an immunological baseline of the tumor microenvironment. After the conclusion of Palbociclib therapy, a post-treatment biopsy will be performed to assess the impact of Palbociclib on the tumor microenvironment; pembrolizumab will be started the same day as the second biopsy. After 2 doses of pembrolizumab, a third (optional) biopsy could be performed. | CDK4/6-Palbociclib | I | Recruiting | NCT06113809 |
| **15** | CDK4/6 Inhibitor Palbociclib Combined With Afatinib as Second-line Treatment for Advanced Squamous Carcinoma of the Esophagus or Gastroesophageal Junction Progressed on at Least First-line Chemotherapy: a Phase 2 Trial | **Arm A.** 6 pts treated with Palbociclib 125 mg po qd on day 1 to 21, Afatinib 40 mg po qd on day 1 to 14, every 28 days as a cycle; If there are ≥ 2 cases of dose-limiting toxicity (DLT), the following 6 pts will reduce the dose to Palbociclib 125mg po qd on day 1 to 21, Afatinib 30mg po qd on day 1 to 28, every 28 days as a cycle; If ≥ 2 cases of DLT occur again, the following 6 pts will reduce the dose to Palbociclib 100mg po qd on day 1 to 21, Afatinib 30mg po qd on day 1 to 28, every 28 days as a cycle; If ≥ 2 cases of DLT occur again, we will analyze the characteristics of adverse events and determine the subsequent dose. | CDK4/6-Palbociclib | II | Recruiting | NCT05865132 |
| **16** | A Randomized Trial of Neratinib, A Pan-ERBB Inhibitor, Alone or in Combination With Palbociclib, a CDK4/6 Inhibitor, in pts With HER2+ Gynecologic Cancers and Other Solid Tumors: A ComboMATCH Treatment Trial | **Arm A.** pts receive neratinib maleate PO QD on days 1-14 of cycle 0 in the absence of disease progression or unacceptable toxicity. Pts then receive neratinib maleate PO QD on days 1-28 of each subsequent cycle. Cycles repeat every 28 days in the absence of disease progression or unacceptable toxicity. Pts who experience progression may crossover to Arm II. Pts undergo ECHO or MUGA during screening and on study, and CT or MRI and collection of blood samples throughout the trial. Pts may also undergo tumor biopsy during screening and on study. **Arm B.** pts receive neratinib maleate PO QD on days 1-14 of cycle 0 in the absence of disease progression or unacceptable toxicity. pts then receive neratinib maleate PO QD on days 1-28 and Palbociclib PO QD on days 1-21 of each subsequent cycle. Cycles repeat every 28 days in the absence of disease progression or unacceptable toxicity. pts undergo ECHO or MUGA during screening and on study, and CT or MRI and collection of blood samples throughout the trial. Pts may also undergo tumor biopsy during screening and on study. | CDK4/6-Palbociclib | II | Recruiting | NCT06126276 |
| **17** | A Phase III Open-label, Multicenter, Randomized Trial of Adjuvant Palbociclib in Combination With Endocrine Therapy Versus Endocrine Therapy Alone for pts With Hormone Receptor Positive / HER2-negative Resected Isolated Locoregional Recurrence of Breast Cancer | **Arm A.** Palbociclib 125 mg/day tablet taken orally for 21 days, followed by 7 days rest for 3 years from randomization, plus standard endocrine therapy for at least 3 years from randomization. **Arm B.** Aromatase inhibitor (anastrozole or exemestane or letrozole) oral daily tablet, or Selective Estrogen Receptor Modulator (SERM) such as tamoxifen oral daily tablet or fulvestrant (Faslodex) injection once every 2 weeks for 3 doses then every month. Premenopausal women and men may also receive an LHRH (luteinizing hormone-releasing hormone) agonist by injection. Standard endocrine therapy will be given for at least 3 years from randomization. | CDK4/6-Palbociclib | III | Recruiting | NCT03820830 |
| **18** | Phase Ib IIT of Heat Shock Protein 90 Inhibitor TAS-116 Combined With Cyclin-dependent Kinase 4/6 Inhibitor Palbociclib in Advanced Breast Cancer Progressing on Palbociclib & Treatment-refractory Solid Tumors With Retinoblastoma Deficiency | **Arm A.** Level 0 Starting Palbociclib with TAS-116. **Arm B.** Level -1 Palbociclib with TAS-116. **Arm C.** Level -2 Palbociclib with TAS-116. | CDK4/6-Palbociclib | I | Recruiting | NCT05655598 |
| **19** | Palbociclib, Trastuzumab, Pyrotinib and Fulvestrant Treatment in pts With Brain Metastasis From ER/PR Positive, HER-2 Positive Breast Cancer: A Multi-center, Prospective Study in China | **Arm A.** ER/PR positive, HER2-positive breast cancer Pts with brain metastatic lesions receive palbociclib PO daily on days 1-21, combined with trastuzumab IV every three weeks, pyrotinib PO daily and fulvestrant IM every 4 weeks. Cycles repeat every 28 days in the absence of disease progression or unacceptable toxicity. | CDK4/6-Palbociclib | II | Recruiting | NCT04334330 |
| **20** | Ulixertinib (BVD-523) in Combination With Palbociclib in pts With Advanced Solid Tumors With Expansion Cohort in Previously Treated Metastatic Pancreatic Cancer and Metastatic RAS-mutant and NF1-mutant (no BRAFV600 Mutations) Melanoma | **Arm A.** Ulixertinib added to palbociclib. | CDK4/6-Palbociclib | I | Recruiting | NCT03454035 |
| **21** | A Phase Ib/II Study to Investigate the Safety, Tolerability, of APG-2575 as a Single Agent or in Combination With Systemic Anti-cancer Agents in pts With ER Positive Breast Cancer or Advanced Solid Tumors. | **Arm A.** APG2575 monotherapy. **Arm B.** APG2575 + palbociclib. | CDK4/6-Palbociclib | I/II | Recruiting | NCT04946864 |
| **22** | A Phase 1B Study to Evaluate the Safety, Tolerability and Preliminary Efficacy of the Combination of Encorafenib, Binimetinib and Palbociclib in pts With BRAF-mutant Metastatic Melanoma (The CELEBRATE Study). | **Arm A.** Encorafenib (tablet) 450mg PO daily. Binimetinib (tablet) 45mg PO BD. Palbociclib (tablet) variable dose PO daily for 21 consecutive days on treatment, followed by 7 consecutive days off treatment in a 28 day cycle. | CDK4/6-Palbociclib | I/II | Recruiting | NCT04720768 |
| **23** | A Phase 2 Trial to Evaluate the Safety and Efficacy of NKT2152 in Combination With Palbociclib (Doublet) and With Palbociclib and Sasanlimab (Triplet) in Subjects With Advanced or Metastatic Clear Cell Renal Cell Carcinoma | **Arm A.** Lead-in Doublet assesses safety of oral dosing NKT2152 at increasing dosage levels in combination with palbociclib to determine a recommended dose for expansion (RDE). **Arm B.** Lead-in Triplet assesses the safety of two doses of NKT2152 identified in the Doublet arm (RDE and RDE-1) by orally dosing ccRCC Pts with NKT2152 in combination with palbociclib and sasanlimab. **Arm C.** Subjects randomized to Arm 1 will receive the Doublet combination (NKT2152 in combination with palbociclib) to provide an assessment of anti-tumor activity and to determine the RP2D. **Arm D.** Subjects randomized to Arm 2 will receive the Triplet therapy (NKT2152 in combination with palbociclib and sasanlimab) to provide an assessment of anti-tumor activity and to determine the RP2D. | CDK4/6-Palbociclib | II | Recruiting | NCT05935748 |
| **24** | A Phase II Clinical Trial Assessing the Safety of Neoadjuvant Palbociclib in Combination With Endocrine Therapy for Post and Pre-menopausal pts With Early Stage Hormone Receptor Positive and Her-2/Neu Negative Breast Cancer | **Arm A.** The following drugs will be taken for six cycles. Palbociclib at a dose of 125 mg should be taken by mouth with food on 21 days and 7 days off schedule (meaning: on Days 1-21 of each 28-day cycle). Letrozole should be taken daily by mouth, every day of each 28-day cycle, at a dose of 2.5 mg. Goserelin is given as subcutaneous injection every 28 days at a dose of 3.6 mg. It is to be given on Day 1 of each cycle. Goserelin will only be administered to pre-menopausal subjects. | CDK4/6-Palbociclib | II | Recruiting | NCT05069038 |
| **25** | A Phase III, Multicenter, Open-label Study of Ribociclib vs. Palbociclib in pts With Advanced Hormone Receptor-positive/HER2-negative/HER2-Enriched Breast Cancer - HARMONIA Trial | **Arm A.** Ribociclib + Fulvestrant or Letrozole. **Arm B.** Palbociclib + Fulvestrant or Letrozole. **Arm C.** Paclitaxel +/- Tislelizumab. | CDK4/6-Palbociclib/ Ribociclib | III | Recruiting | NCT05207709 |
| **26** | Phase II Study of CDK 4/6 Inhibitor, LEE011 (Ribociclib), in Combination With Adjuvant Endocrine Therapy at Varying Duration for ER-positive Breast Cancer (LEADER). | **Arm A.** Ribociclib + endocrine therapy. **Arm B.** endocrine therapy. | CDK4/6-Ribociclib | II | Recruiting | NCT03285412 |
| **27** | A ctDNA Screening Program in pts With HR+, HER2- Metastatic Breast Cancer for Detection of High-risk Relapse pts on Any CDK4/6 Inhibitor and a Randomised Phase II Study Comparing Alpelisib Combined With Fulvestrant to Ribociclib Combined With Fulvestrant, in pts With Persistent Targetable PIK3CA Mutations | **Arm A.** Oral alpelisib (300 mg daily, in 28-day cycle) and fulvestrant as per standard practice. Moreover, men and premenopausal women will receive an LH-RH analogue (goserelin, leuprorelin, or triptorelin) every 28 days ±3 days, as per standard practice. **Arm B.** Oral ribociclib (600 mg daily, 3 weeks on, then 1 week off treatment in 28-day cycles) and fulvestrant as per standard practice. Moreover, men and premenopausal women will receive an LH-RH analogue (goserelin, leuprorelin, or triptorelin) every 28 days ±3 days, as per standard practice. | CDK4/6-Ribociclib | II | Recruiting | NCT05625087 |
| **28** | A Phase I/II, Single Arm, Non-randomized Study of Ribociclib (LEE011), a CDK 4/6 Inhibitor, in Combination With Bicalutamide, an Androgen Receptor (AR) Inhibitor, in Advanced AR+ Triple-negative Breast Cancer: Big Ten Cancer Research Consortium BRE15-024 | **Arm A.** Dose Escalation Cohort 1 will consist of 3-6 Pts who will receive bicalutamide 150mg PO daily on days 1-28 of a 28 day cycle and ribociclib 400mg PO daily on days 1-21 of a 28 day cycle. Cohort 2 will consist of 3-6 Pts who will receive bicalutamide 150mg PO daily on days 1-28 of a 28 day cycle and ribociclib 400mg PO daily on days 1-28 of a 28 day cycle. Cohort 3 will consist of 3-6 Pts who will receive bicalutamide 150mg PO daily on days 1-28 of a 28 day cycle and ribociclib 600mg PO daily on days 1-21 of a 28 day cycle. **Arm B.** The maximum safe dose of ribociclib in combination with bicalutamide will be given to up to 25 Pts. | CDK4/6-Ribociclib | I/II | Recruiting | NCT03090165 |
| **29** | A Phase I/Ib Trial of the CDK4/6 Antagonist Ribociclib And The HDAC Inhibitor Belinostat In pts With Metastatic Triple Negative Breast Cancer And Recurrent Ovarian Cancer With Response Prediction By Genomics (CHARGE) | **Arm A.** Ribociclib and belinostat will be given at escalating doses and on multiple administration schedules throughout the dose escalation component of the study. The MTD identified in the dose escalation component will be used to define the dose and administration schedule used in the dose expansion. | CDK4/6-Ribociclib | I | Recruiting | NCT04315233 |
| **30** | Comparison of Clinical Efficacy Between Letrozole + Ribociclib and Fulvestrant + Letrozole + Ribociclib in Hormone Receptor Positive, HER2 Negative Metastatic Breast Cancer - a Randomized, Phase 2 Study | **Arm A.** Fulvestrant + AI + Ribociclib. **Arm B.** AI + Ribociclib. | CDK4/6-Ribociclib | II | Recruiting | NCT05816655 |
| **31** | Phase II Trial of SMO/AKT/ NF2/CDK Inhibitors in Progressive Meningiomas With SMO/AKT/ NF2/CDK Pathway Mutations | **Arm A.** Pts receive Vismodegib (PO QD). Cycles repeat every 28 days in the absence of disease progression or unacceptable toxicity. **Arm B.** Pts receive FAK inhibitor GSK2256098 (PO BID). Cycles repeat every 28 days in the absence of disease progression or unacceptable toxicity. **Arm C.** Pts receive Capivasertib (PO BID) on days 1-4. Treatment repeats every 7 days for up to 1 cycle (28 days) in the absence of disease progression or unacceptable toxicity. **Arm D.** Pts receive Abemaciclib (PO Q12H). Cycles repeat every 28 days in the absence of disease progression or unacceptable toxicity. | CDK4/6- Abemaciclib | II | Recruiting | NCT02523014 |
| **32** | Phase II Trial of Fulvestrant Plus Abemaciclib With or Without Run-in of Fulvestrant in Er-Positive, Her2-Negative Metastatic Breast Cancer After Failure of a CDK4/6 Inhibitor In Combination With an Aromatase Inhibitor | **Arm A.** A 1-month (28 days) run-in of fulvestrant will precede fulvestrant plus abemaciclib treatment. Fulvestrant at a dose of 500 mg will be administered intramuscularly (IM) into the buttocks slowly (1-2 minutes per injection) as two 5-mL injections, one in each buttock, on Days 1 and 15 of the run-in period. After the fulvestrant run-in, fulvestrant plus abemaciclib will be administered in 28-day cycles until disease progression or unacceptable toxicity. Fulvestrant (500 mg IM) will be administered on Day 1 of each 28-day cycle. Abemaciclib at a dose of 150 mg will be given p.o. BID on Days 1-28 of each cycle | CDK4/6-Abemaciclib | II | Recruiting | NCT05305924 |
| **33** | A Phase II Study of the CDK4/6 Inhibitor Abemaciclib in pts With Solid Tumors Harboring Genetic Alterations in Genes Encoding D-type Cyclins or Amplification of CDK4 or CDK6 | **Arm A.** Participants with CCND1, CCND2, or CCND3 receive Abemaciclib. **Arm B.** Participants with CDK4 or CDK6 receive Abemaciclib. | CDK4/6-Abemaciclib | II | Recruiting | NCT03310879 |
| **34** | Single Cell Immune and Non-immune Correlates of Response to Neoadjuvant Abemaciclib and Letrozole in Hormone Receptor Positive Breast Cancer | **Arm A.** pts will take twice daily abemaciclib and daily letrozole (Arm A) for 2 weeks prior to your planned standard treatment for breast cancer. In addition to tests and procedures that are part of your standard care, you will have a biopsy and blood draw for study purposes prior to starting study treatment. **Arm B.** pts will take daily letrozole only (Arm B) according to treatment arm for 2 weeks prior to your planned standard treatment for breast cancer. In addition to tests and procedures that are part of your standard care, you will have a biopsy and blood draw for study purposes prior to starting study treatment. | CDK4/6-Abemaciclib | II | Recruiting | NCT04614194 |
| **35** | Targeted Therapy With CDK4/6 Inhibitors in Chemo- Refractory/Relapsed, Rb Wild-type Extensive Small Cell Lung Cancer (SCLC), Large Cell Neuroendocrine Lung Cancer, Extrapulmonary Small Cell Cancers and Other High Grade Neuroendocrine Cancers of the Lung, an Open Label Phase 2 Trial. | **Arm A.** Subjects will receive Abemaciclib (200 mg), orally every 12 hours on days 1 to 28 of a 28-day cycle for a total of 56 doses per cycle. Subjects will be evaluated after 4 weeks (1st cycle) and then every 8 weeks (2 cycles) with radiographic imaging to assess response to treatment. | CDK4/6-Abemaciclib | II | Recruiting | NCT04010357 |
| **36** | Genetically-informed Therapy for ER+ Breast Cancer in a Post-CDK4/6 Inhibitor Setting: a Phase II Umbrella Study (GERTRUDE) | **Arm A.** Participants with a qualifying ERBB2 (HER2) mutation will be assigned to Treatment Arm A and given the combination of neratinib and fulvestrant until the end of the primary treatment phase. Fulvestrant (500 mg) will be administered by intramuscular injection into the buttocks on Cycle 1 Day 1 and 15, and on Day 1 of subsequent Cycles. Neratinib will initially be administered orally in 3 tablets (total dose of 120 mg) taken 1 time per day with food on Cycle 1 Days 1-7, in combination with fulvestrant starting on Cycle 1 Day 1 as described above. The dose of neratinib will be increased to 4 tablets (total dose of 160 mg) taken 1 time per day with food on Cycle 1 Days 8-14, and then increased further to 6 tablets (240 mg) taken once daily with food thereafter. **Arm B.** If a participant does not have a qualifying ERBB2 (HER2) mutation, but they have a qualifying PIK3CA mutation, the subject will be assigned to Treatment Arm B and given the combination of alpelisib and fulvestrant until the end of the primary treatment phase. Fulvestrant (500 mg) will be administered by intramuscular injection into the buttocks on Cycle 1 Day 1 and Day 15, and on Day 1 of subsequent Cycles. Alpelisib will be administered orally in 2 tablets (total dose of 300 mg) taken 1 time per day with food, in combination with fulvestrant as described above. **Arm C.** If a subject does not have a qualifying ERBB2 or PIK3CA mutation, but they have a qualifying mutation/alteration in AKT1, MTOR, or PTEN, the subject will be assigned to Treatment Arm C and given the combination of everolimus and fulvestrant until the end of the primary treatment phase. Fulvestrant (500 mg) will be administered by intramuscular injection into the buttocks on Cycle 1 Day 1 and 15, and on Day 1 of subsequent Cycles. Everolimus will be administered orally in 1 tablet (10 mg per tablet) taken 1 time per day, in combination with fulvestrant as described above. **Arm D.** If a participant does not have a qualifying mutation/alteration for Arms A/B/C, and the participant does not have mutation or loss of RB1, the subject will be assigned to Treatment Arm D and given the combination of abemaciclib and fulvestrant until the end of the primary treatment phase. Fulvestrant (500 mg) will be administered by intramuscular injection into the buttocks on Cycle 1 Day 1 and Day 15, and on Day 1 of subsequent Cycles. Abemaciclib will be administered orally in 1 tablet (150 mg) taken 2 times per day, in combination with fulvestrant as described above. | CDK4/6-Abemaciclib | II | Recruiting | NCT05933395 |
| **37** | A Phase I/II Study of the Cyclin-Dependent Kinase(CDK)4/6 Inhibitor Abemaciclib for Neurofibromatosis Type 1 (NF1) Related Atypical Neurofibromas | **Arm A.** Abemaciclib orally twice daily at escalating doses to determine the MTD/RP2D. **Arm B**. Abemaciclib orally twice daily at the RP2D | CDK4/6-Abemaciclib | I/II | Recruiting | NCT04750928 |
| **38** | RADIANT Study: Pre-op Radiation With Abemaciclib Andletrozole in Early Stage Breast Cancer | **Arm A.** Part A: 28-day treatment cycle based on abemaciclib 150mg BID (twice a day). Letrozole 2.5mg daily and Abemaciclib 150mg twice a day for three cycles prior to undergoing radiation therapy. On-treatment biopsy conducted between cycle 3, day 16, and cycle 4, day 1. This occurs two weeks prior to transitioning to Part B.Part B: Continue treatment from Part A, 28-day cycle based on abemaciclib 150mg BID (twice a day) with letrozole 2.5mg daily. Administration of radiation therapy following the three cycles of combined Abemaciclib and Letrozole. Part C: Two cycles of abemaciclib, 150mg twice a day. Letrozole 2.5mg daily. Part D: surgery. | CDK4/6-Abemaciclib | I | Recruiting | NCT06139107 |
| **39** | A Phase 1 Study of BET Bromodomain Inhibitor ZEN003694 in Combination With the CDK4/6 Inhibitor Abemaciclib in pts With NUT Carcinoma and Other Solid Tumors | Patients receive ZEN003694 PO QD on days 1-28 or 5 days on and 2 days off, and abemaciclib PO BID on days 1-28 of each cycle. Cycles repeat every 28 days in the absence of disease progression or unacceptable toxicity. Patients undergo imaging evaluation, blood sample collection and tumor biopsy throughout the study. | CDK4/6-Abemaciclib | I | Recruiting | NCT05372640 |
| **40** | A Phase 1/2 Study of CB-103 (Oral Pan-NOTCH Inhibitor) With Abemaciclib or Lenvatinib in Combination in pts With NOTCH Activated Adenoid Cystic Carcinoma (CALCulus) | **Arm A.** CB-103 + Abemaciclib. Days 1- 28 of 28-day cycle: Predetermined dose of CB-103 2x daily on five consecutive days followed by two days of treatment break in each treatment week. **Arm B.** Days 1- 28 of 28-day cycle: Predetermined dose of CB-103 2x daily on five consecutive days followed by two days of treatment break in each treatment week and predetermined dose of Abemaciclib 1x daily. Therapy will continue until disease progression, therapy intolerance, or participant withdrawal. End of Treatment (EOT) visit within 30 days of last administration of study treatments. **Arm C.** Lenvatinib + CB-103. Days 1- 28 of 28-day cycle: Predetermined dose of CB-103 2x daily on five consecutive days followed by two days of treatment break in each treatment week. **Arm D.** Lenvatinib + CB-103. Predetermined dose of CB-103 2x daily on five consecutive days followed by two days of treatment break in each treatment week and predetermined dose of Abemaciclib 1x daily. Therapy will continue until disease progression, therapy intolerance, or participant withdrawal. End of Treatment (EOT) visit within 30 days of last administration of study treatments. | CDK4/6-Abemaciclib | I/II | Recruiting | NCT05774899 |
| **41** | PreOperative Endocrine Therapy for Individualised Care With Abemaciclib | **Arm A.** Endocrine therapy prescribed as per standard of care, for an expected duration of at least 5 years, or until evidence of disease recurrence or other discontinuation criteria are met. Choice of endocrine therapy may include non-steroidal aromatase inhibitor (letrozole or anastrozole), steroidal aromatase inhibitor (exemestane), or tamoxifen. **Arm B.** Abemaciclib administered at dose of 150mg twice daily (provided as 50mg tablets), for 2 years or until evidence of disease recurrence or other discontinuation criteria are met. Endocrine therapy prescribed as per standard of care, for an expected duration of at least 5 years, or until evidence of disease recurrence or other discontinuation criteria are met. Choice of endocrine therapy may include non-steroidal aromatase inhibitor (letrozole or anastrozole), steroidal aromatase inhibitor (exemestane), or tamoxifen | CDK4/6-Abemaciclib | III | Recruiting | NCT04584853 |
| **42** | Genomically-Guided Treatment Trial in Brain Metastases | **Arm A.** (CDK gene mutation) Patients receive abemaciclib PO BID on days 1-28. Cycles repeat every 28 days in the absence of disease progression or unacceptable toxicity. **Arm B.** Patients receive PI3K inhibitor paxalisib PO QD on days 1-28. Cycles repeat every 28 days in the absence of disease progression or unacceptable toxicity. Arm C. (NTRK/ROS1 gene mutation). Patients receive entrectinib PO QD on days 1-28. Cycles repeat every 28 days in the absence of disease progression or unacceptable toxicity. **Arm C.** (KRAS G12C mutation) Patients receive adagrasib (MRTX849) PO BID on days 1-28. Cycles repeat every 28 days in the absence of disease progression or unacceptable toxicity. | CDK4/6-Abemaciclib | II | Recruiting | NCT03994796 |
| **43** | An Open Label Phase II Study of the Efficacy and Safety of Abemaciclib, a Cyclin Dependent Kinase (CDK4/6) Inhibitor in Selected pts With Recurrent Ovarian or Endometrial Cancer | **Arm A.** Patients receive abemaciclib PO BID on days 1-28. Patients with tumors that are hormone receptor positive also receive and anastrozole or letrozole per standard of care. Cycles repeat every 28 days in the absence of disease progression or unacceptable toxicity. | CDK4/6-Abemaciclib | II | Recruiting | NCT04469764 |
| **44** | A Phase 2 Trial of the CDK4/6 Inhibitor Abemaciclib in pts With Advanced and Refractory Well-Differentiated Gastroenteropancreatic Neuroendocrine Tumors (GEP NETs) | **Arm A.** Treatment (abemaciclib). Patients receive abemaciclib PO BID on days 1-28. Cycles repeat every 28 days in the absence of disease progression or unacceptable toxicity. | CDK4/6-Abemaciclib | II | Recruiting | NCT03891784 |
| **45** | A Phase 0/2 Study of LY3214996 (ERK Inhibitor) in Combination With Abemaciclib (CDK4 and 6 Inhibitor) in Recurrent Glioblastoma Participants Scheduled for Resection to Evaluate Central Nervous System (CNS) Penetration | **Arm A.** 400 mg of LY3214996 QD for 6 doses and 100 mg of Abemaciclib BID for 11 doses over 5.5 days prior to surgical resection. On Day 6, participants will receive Abemaciclib + LY3214996 dose 7 to 9 hours prior to craniotomy for tumor resection. | CDK4/6-Abemaciclib | 0/II | Recruiting | NCT04391595 |
| **46** | Phase I/II Study of CDK4/6 Inhibition With Abemaciclib to Upregulate PSMA Expression Prior to 177Lu-PSMA-617 Treatment in pts With Metastatic Castrate Resistant Prostate Cancer (mCRPC) Previously Treated With Novel Hormonal Agents and Chemotherapy | **Arm A.** Abemaciclib, 177Lu-PSMA-617. Patients receive abemaciclib lead-in on days 1-14 and lutetium Lu 177 vipivotide tetraxetan IV over 30 minutes on day 15. Treatment repeats every 6 weeks for up to 4 cycles in the absence of disease progression or unacceptable toxicity. **Arm B.** Recommended Phase 2 dose of Abemaciclib, 177Lu-PSMA-617. Patients receive the recommended phase 2 dose of abemaciclib lead-in on days 1-14 and lutetium Lu 177 vipivotide tetraxetan IV over 30 minutes on day 15. Treatment repeats every 6 weeks for up to 4 cycles in the absence of disease progression or unacceptable toxicity. | CDK4/6-Abemaciclib | I/II | Recruiting | NCT05113537 |
| **47** | The TRADE Study: A Phase 2 Trial to Assess the ToleRability of Abemaciclib Dose Escalation in pts With Early-Stage HR-positive and HER2-negative Breast Cancer | **Arm A.** Abemaciclib. Cycles 1 - 24. Days 1 - 28 of 28-day cycle: Predetermined dose of Abemaciclib 2 x per day. Endocrine therapy 1 x per day. In clinic visits with blood tests, questionnaires, and assessments. Day 1 of Cycles 1, 2, and 3. Day 15 of Cycles 1 and 2. Every three cycles after Cycle 3 Day 1. End of treatment visit with blood tests, questionnaires, assessments, and stool sample collection. | CDK4/6-Abemaciclib | II | Recruiting | NCT06001762 |
| **48** | PARP Inhibitor in Combination With CDK4/6 Inhibitor and Endocrine Therapy as the First-line Therapy for HR+/ HER2-Advanced Breast Cancer | **Arm A.** In this cohort, a patient would receive Dalpiciclib (CDK4/6 inhibitor) combined with Fluzoparib (PARP inhibitor) and endocrine therapy. **Arm B.** In this cohort, a patient would receive Dalpiciclib (CDK4/6 inhibitor) combined with endocrine therapy. | CDK4/6-Abemaciclib | II | Recruiting | NCT05759546 |
| **49** | Apatinib in Combination With CDK4/6 Inhibitor and Endocrine Therapy as the First-line Therapy for HR+/ HER2-Advanced Breast Cancer | **Arm A.** In this cohort, a patient would receive Dalpiciclib (CDK4/6 inhibitor) combined with Apatinib and endocrine therapy. **Arm B.** In this cohort, a patient would receive Dalpiciclib(CDK4/6 inhibitor) combined with endocrine therapy. | CDK4/6-Dalpiciclib | II | Recruiting | NCT05759572 |
| **50** | A Phase 2 Multicenter, Open-Label Study of the CDK4/6 Inhibitor SPH4336 in Subjects With Locally Advanced or Metastatic Liposarcomas | **Arm A.** SPH4336 400 mg (2 - 200 mg tablets) orally, daily. | CDK4/6-SPH4336 | II | Recruiting | NCT05580588 |
| **51** | Explore the Efficacy and Safety of Dalpiciclib Combined With Fluvestrant and Compound Gossypol Acetate Tablets in Advanced HR-positive and HER2-negative Breast Cancer After CDK4/6 Treatment Failed. | **Arm A.** Darcilie: 150mg orally for three weeks and stop for one week. Fluvestrant: 500mg every four weeks. A further 500mg dose two weeks after the first dose. Compound gossyrol acetate tablet: 20mg daily. | CDK4/6-Dalpiciclib | II | Recruiting | NCT06133088 |
| **52** | An Exploratory Study on Predicting the Efficacy of Dalpiciclib in Combination With Endocrine Therapy for HR-Positive and HER2-Negative Recurrent/Metastatic Breast Cancer pts After CDK4/6 Inhibitor Treatment Failure Using 18F-FES PET/CT | **Arm A.** The combination of Dalpiciclib with physician-selected endocrine therapy. **Arm B**. Chemotherapy selected by the physician. | CDK4/6-Dalpiciclib | III | Recruiting | NCT05861830 |
| **53** | Dalpiciclib Combined With Camrelizumab for PD-1 Inhibitor Refractory R/M NPC | **Arm A.** Dalpiciclib, orally daily from day 1 to 21, Q4W. Camrelizumab, intravenously, 200mg, on day 1, Q3W. | CDK4/6-Dalpiciclib | II | Recruiting | NCT05724355 |
| **54** | Study of Efficacy of Everolimus Combined With First-line Endocrine Therapy for HR+/HER2- (Open, Randomized, Phase II ) | **Arm A.** Everolimus + CDK4/6 inhibitor+ Endocrine therapy group: Everolimus, 10mg po. qd; Dalpiciclib 125mg po. qd. for 3 weeks, followed by 1 week off, 4 weeks as a cycle. Aromatase inhibitors (Letrozole/Anastrozole/Exemestane), po. qd. at specific doses (Letrozole 2.5mg/day; Anastrozole 1mg/day, Exemestane 25mg/day); Or Fluvestrant, 500mg im. q28d, (Extra 500mg given after 2 weeks of first dose); Premenopause participants: Goserelin 3.6mg, subcutaneously, once every 4 weeks. **Arm B.** CDK4/6 inhibitor+ Endocrine therapy group: Dalpiciclib 125mg po. qd. for 3 weeks, followed by 1 week off, 4 weeks as a cycle. Aromatase inhibitors (Letrozole/Anastrozole/Exemestane), po. qd. at specific doses (Letrozole 2.5mg/day; Anastrozole 1mg/day, Exemestane 25mg/day); Or Fluvestrant, 500mg im. q28d, (Extra 500mg given after 2 weeks of first dose); Premenopause participants: Goserelin 3.6mg, subcutaneously, once every 4 weeks. | CDK4/6-Dalpiciclib | II | Recruiting | NCT05949541 |
| **55** | A Phase II/III Study of SPH4336 in Combination With Endocrine Therapy in the Treatment of HR-positive, HER2-negative Locally Advanced or Metastatic Breast Cancer That Progressed on CDK4/6 Inhibitor Combined With Endocrine Therapy | **Arm A.** SPH4336 Tablets; Letrozole tablets; Fulvestrant injection. **Arm B.** SPH4336 Tablets Placebo; Letrozole tablets; Fulvestrant injection | CDK4/6- SPH4336 | II/III | Recruiting | NCT05860465 |
| **56** | A Phase 1a/1b Study Investigating the Safety, Tolerability, Pharmacokinetics, Pharmacodynamics, and Preliminary Antitumor Activity of the CDK4 Inhibitor BGB-43395, Alone or as Part of Combination Therapies in Chinese pts With Advanced or Metastatic HR+/HER2- Breast Cancer and Other Solid Tumors | **Arm A.** Sequential cohorts of increasing dose levels of BGB-43395 will be evaluated as monotherapy and in combination with either fulvestrant or letrozole. **Arm B.** The recommended dose for expansion (RDFE) for BGB-43395 in combination with fulvestrant from Phase 1a will be evaluated in HR+ breast cancer and selected tumor cohorts. | CDK4 – BGB-43395 | I | Recruiting | NCT06253195 |
| **57** | A Phase 1a/1b Study Investigating the Safety, Tolerability, Pharmacokinetics, Pharmacodynamics, and Preliminary Antitumor Activity of the CDK4 Inhibitor BGB-43395, Alone or as Part of Combination Therapies in pts With Metastatic HR+/HER2- Breast Cancer and Other Advanced Solid Tumors | **Phase 1a:** Sequential cohorts of increasing dose levels of BGB-43395 will be evaluated as monotherapy and in combination with either fulvestrant or letrozole to assess for safety and tolerability. **Phase 1b:** The recommended dose for expansion (RFDE) for BGB-43395 (in combination with fulvestrant or letrozole) from Phase 1a will be evaluated in HR+ breast cancer and selected tumor-specific cohorts. | CDK4 – BGB-43395 | I | Recruiting | NCT06120283 |
| **58** | AN INTERVENTIONAL, OPEN-LABEL, RANDOMIZED, MULTICENTER PHASE 3 STUDY OF PF-07220060 PLUS FULVESTRANT COMPARED TO INVESTIGATOR'S CHOICE OF THERAPY IN PARTICIPANTS OVER 18 YEARS OF AGE WITH HORMONE RECEPTOR-POSITIVE, HER2-NEGATIVE ADVANCED/METASTATIC BREAST CANCER WHOSE DISEASE PROGRESSED AFTER PRIOR CDK 4/6 INHIBITOR BASED THERAPY | **Arm A.** PF-07220060 to be taken by mouth as a tablet in combination with fulvestrant (a solution for injection). **Arm B.** Fulvestrant alone (a solution for injection), or everolimus in combination with exemestane, both a tablet to be taken by mouth. | CDK4/6-PF-07220060 | III | Recruiting | NCT06105632 |
| **59** | A Phase II Study of the CDK4/6 Inhibitor Dalpiciclib Combined With Letrozole in Unresectable Refractory or Resistant Recurrent HR+/HER2 - Gynecologic Solid Tumors | **Arm A.** Dalpiciclib 150mg qd, (day 1-21), once every 4 weeks. Letrozole 2.5mg qd, repeated once every 4 weeks. | CDK4/6-Dalpiciclib | II | Recruiting | NCT06243185 |
| **60** | A Phase 1/2a Study to Evaluate the Tolerability, Safety, Pharmacokinetics and Efficacy of BPI-1178 Alone in Advanced Solid Tumor and of BPI-1178 in Combination With Endocrine Therapy in Advanced HR+/HER2- Breast Cancer | **Arm A.** Participants will first receive single dose BPI-1178 orally at dose levels of 25mg, 75mg, 150mg, 250mg, 400mg and 500mg followed by a 7-day washout period , and then start receiving the 28 days/cycle continuous treatment until disease progression or unacceptable toxicity. After the 500 mg dose escalation trial is completed, the PK study will be conducted for the 400 mg dose group, the 300 mg dose group and the 200 mg dose group. **Arm B.** Participants will receive BPI-1178 at dose levels of MTD, MTD-1 or MTD-2 in combination with fulvestrant for 3 consecutive weeks, followed by 1 week drug withdrawal or continuous dosing for 28 days, in each 28-day treatment cycle, until disease progression or unacceptable toxicity. **Arm C.** Participants will receive BPI-1178 at dose levels of MTD, MTD-1 or MTD-2 in combination with letrozole for 3 consecutive weeks, followed by 1 week drug withdrawal or continuous dosing for 28 days, in each 28-day treatment cycle, until disease progression or unacceptable toxicity. | CDK4/6 - BPI-1178 | I/II | Recruiting | NCT04282031 |
| **61** | MegaMOST - A Multicenter, Open-label, Biology Driven, Phase II Study Evaluating the Activity of Anti-cancer Treatments Targeting Tumor Molecular Alterations /Characteristics in Advanced / Metastatic Tumors. | **Arm A.** HDM201 + Ribociclib. Patient with documented amplification of Cyclin-dependent kinase 6 (CDK6) and/or Cyclin-dependent kinase 4 (CDK4), and/or cyclin dependent kinase inhibitor 2A (CDKN2A) homozygous deletion, and/or amplification of Cyclin D1 (CCND1) and/or Cyclin D3 (CCND3) with no deletion/losses more than single copy of retinoblastoma 1 (RB1) by copy number and P53 wild-type detected on tumor sample from primary tumor or metastatic lesion. **Arm B.** Cabozantinib. Patient with AXL, MET, vascular endothelial growth factor receptor (VEGFR), vascular endothelial growth factor (VEGF), RET, ROS1, MER, Tropomyosin receptor kinase B (TRKB),TIE-2 and/or Tyro3 activating mutations and/or amplification, and/or NTRK translocation and/or ROS1 translocation, and/or MET translocation detected on tumor sample from primary tumor or metastatic lesion. **Arm C.** Alectinib. Activating ALK alterations: translocation, or selected mutations. **Arm D.** Regorafenib. Patient with activating mutation and/or amplification of VEGFR1-3, TIE-2, KIT, RET, RAF1, BRAF (other than V600 mutations), CRAF, HRAS, Platelet Derived Growth Factor Receptor (PDGFR), Fibroblast Growth Factor Receptor 1-2 (FGFR1-2), FLT3 and/or Colony Stimulating Factor 1 Receptor (CSF1R), and/or amplification of the ligands, and/or biallelic inactivation of SMAD4. **Arm E.** Trametinib. Patient with activating mutation and/or amplification of KRAS (except all KRAS G12 mutations), NRAS, HRAS and/or Mitogen-Activated Protein Kinase Kinase (MAP2K); and/or biallelic inactivation of Neurofibromin 1 (NF1); and/or activating mutation Protein Tyrosine Phosphatase Non-Receptor Type 11 (PTPN11); and/or amplification or translocation of BRAF ; and/or translocation RAF1. Arm F. Trametinib + Dabrafenib. Patient with BRAF V600 mutation. **Arm G.** Avapritinib. Activating mutations of KIT exon 17 or PDGFRA exon 18 associated or not to mutation on KIT exon 11 or PDGFRA exon 12/14 | CDK4/6-Ribociclib | II | Recruiting | NCT04116541 |
| **62** | Efficacy and Safety of Neoadjuvant Dalpiciclib Combined With Endocrine Therapy in Luminal B/HER2-negative Breast Cancer and Biomarker Analysis: a Single-arm, Open-label Trial | **Arm A.** Dalpiciclib combined with letrozole or anastrozole | CDK4/6-Dalpiciclib | II | Recruiting | NCT05640778 |
| **63** | Adebrelimab Combined With Dalpiciclib and Standard Endocrine Therapy for HR+/HER2 - Advanced Breast Cancer:a Single-arm, Phase II Exploratory Clinical Study | **Arm A.** Adebrelimab (1200mg intravenously ,Q3W) + Dalpiciclib (150mg once a day for 3 weeks, stop for 1 week, Q4W) + Endocrine recommended drugs untreated: aromatase inhibitors (letrozole/anastrozole/exemestane), given orally once daily at a specific dose (letrozole 2.5mg/ day; Anastrozole 1mg/ day, exemestane 25mg/ day); first-line endocrine therapy failed: fluvestrant was given once every 28 days, 500mg intramuscular injection, and then 500mg intramuscular injection 2 weeks after the first administration | CDK4/6-Dalpiciclib | II | Recruiting | NCT06149130 |
| **64** | Pyrotinib Maleate, Trastuzumab, SHR6390(Dalpiciclib) and Letrozole in Combination for Neoadjuvant Treatment of Stage II-III TPBC | **Arm A.** Combined treatment of pyrotinib maleate, CDK4/6 inhibitor dalpiciclib, trastuzumab and letrozole. The effectiveness of the combined treatment will be evaluated by MRI every two treatment cycles. If the disease progresses, the participant will withdraw from the trial. If the combined treatment has identified effectiveness, the participant will undergo surgical treatment within 4 weeks (over 2 weeks) after termination of the neoadjuvant treatment. The patients will be followed up for 5 years | CDK4/6-Dalpiciclib | II | Recruiting | NCT05228951 |
| **65** | Phase 1, Open-label, Study of Voruciclib in Subjects With Relapsed and/or Refractory B Cell Malignancies or AML After Failure of Prior Standard Therapies and Voruciclib in Combination With Venetoclax in Subjects With Relapsed/Refractory AML | **Arm A.** Voruciclib monotherapy - Open-label, 3 + 3 dose escalation study which may enroll up to 6 subjects at each dose level and disease type (AML or B-cell malignancies). Voruciclib and Venetoclax - Open-label, 3 + 3 dose escalation study which may enroll up to 6 subjects at each dose level for AML subjectsL subjects. | CDK9 - Voruciclib | I | Recruiting | NCT03547115 |
| **66** | Cetuximab Plus Dalpicilib in the Second-line Treatment of pts With HPV Negative, PD-1 Resistant Recurrent/Metastatic Head and Neck Squamous Cell Carcinoma: an Open-label,Single Arm,Phase 2 Trial | **Arm A.** Cetuximab: starting dose of 400 mg/m2 + maintenance dose of 250 mg/m2. Dalpiciclib: 150 mg once daily for 21 days, followed by 7 days of discontinuation (3/1 dosing regimen) for a 28-day treatment cycle. Subjects will continue treatment with cetuximab in combination with dalpiciclib until termination criteria are met. | CDK4/6-Dalpiciclib | II | Recruiting | NCT05721443 |
| **67** | A PHASE 1/2A STUDY EVALUATING THE SAFETY, TOLERABILITY, PHARMACOKINETICS, PHARMACODYNAMICS, AND ANTI-TUMOR ACTIVITY OF PF-07220060 AS A SINGLE AGENT AND AS PART OF COMBINATION THERAPY IN PARTICIPANTS WITH ADVANCED SOLID TUMORS | **Arm A.** PF-07220060 Monotherapy Escalation. **Arm B**. PF-07220060 with Letrozole combination Escalation. **Arm C.** PF-07220060 with Fulvestrant Combination Escalation. Arm D. PF-07220060 Monotherapy Food Effect **Arm E**. PF-07220060 with Letrozole Combination Expansion. **Arm F**. PF-07220060 with fulvestrant Combination Expansion. **Arm G.** PF-07220060 with Letrozole Combination Expansion. **Arm H.** PF-07220060 with Enzalutamide Escalation. **Arm I.** PF-07220060 DDI with Midazolam. **Arm J.** PF-07220060 with enzalutamide Combination Expansion. | CDK4/6 - PF-07220060 | I/II | Recruiting | NCT04557449 |
| **68** | A Single-arm, Exploratory Clinical Study of Trilaciclib Combined With mFOLFIRINOX Regimen in the Treatment of pts With Advanced Pancreatic Cancer | **Arm A.** Trilaciclib (240mg/m2 IV infusion, D1, D2, Q2W), Oxaliplatin( 68mg/m2 IV infusion, D1), Irinotecan (135mg/m2 IV infusion D1), Leucovorin (400mg/m2 IV infusion D1), 5-FU (2.4g/m2 IV infusion for 46h, D1); a total of 12 cycles of treatment were performed every 14 days as a cycle. | CDK4/6 - Trilaciclib | II | Recruiting | NCT06151262 |
| **69** | A Phase 1/2, Open-label, Multicenter Study to Investigate the Safety, Pharmacokinetics, and Efficacy of Fadraciclib (CYC065), an Oral CDK 2/9 Inhibitor, in Subjects With Advanced Solid Tumors and Lymphoma | **Arm A.** Phase I = Fadraciclib administered orally in escalating doses starting at 50mg bid MWF for 3 weeks of a 4 week cycle. Subsequent cohorts will escalate in dose and schedule until optimized phase 2 dose and schedule is achieved. Phase 2 = Recommended Fadraciclib phase 2 dose and schedule administered orally in 28 day cycles. | CDK2/9 - Fadraciclib (CYC065) | I/II | Recruiting | NCT04983810 |
| **70** | A Phase I/IIa, Open-Label Dose Escalation and Dose Expansion Study of Intravenous GFH009 Single Agent and in Combination With Venetoclax and Azacitidine in pts With Relapsed/Refractory Hematologic Malignancies | **Arm A.** Dose escalation in patients with r/r AML. In the dose escalation part, the dose levels will be escalated following the Bayesian optimal interval (BOIN) design. **Arm B**. Dose escalation in patients with r/r CLL/SLL or lymphoma. In the dose escalation part, the dose levels will be escalated following the Bayesian optimal interval (BOIN) design. **Arm C**. 45 mg QW in patients with r/r AML. SLS009 (45 mg QW) in combination with venetoclax and azacitidine in patients with r/r AML who have relapsed on or are refractory to venetoclax-based regimens. **Arm D.** 60 mg QW in patients with r/r AML. SLS009 (60 mg QW) in combination with venetoclax and azacitidine in patients with r/r AML who have relapsed on or are refractory to venetoclax-based regimens. **Arm E.** 30 mg BIW in patients with r/r AML. SLS009 (30 mg BIW) in combination with venetoclax and azacitidine in patients with r/r AML who have relapsed on or are refractory to venetoclax-based regimens. **Arm F.** 30 mg BIW in patients with r/r AML with ASXL1 mutation. SLS009 (30 mg BIW) in combination with venetoclax and azacitidine in patients with r/r AML who have relapsed or are refractory to venetoclax-based regimens and with documented ASXL1 mutation. **Arm G**. 30 mg BIW in pts with r/rAML with other than ASXL1 mutations. SLS009 (30 mg BIW) in combination with venetoclax and azacitidine in patients with r/r AML who have relapsed or are refractory to venetoclax-based regimens and with documented Defining somatic mutations, Cytogenetic abnormalities defining acute myeloid leukemia, myelodysplasia related, other than ASXL1 mutation per WHO 5th Edition classification. | CDK9 - GFH009 | I/II | Recruiting | NCT04588922 |
| **71** | A Phase 1/2 Multicenter, Open-label, Dose-escalation, Safety, Pharmacodynamic, and Pharmacokinetic Study of Q901 Administered Via Intravenous Infusion in Adult pts With Selected Advanced Solid Tumors With a Cohort Expansion at the Recommended Phase 2 Dose | **Arm A.** Dose escalation (Q901). **Arm B.** Q901 Single-Agent Expansion Cohorts. **Arm C.** Q901 + Pembrolizumab Cohorts | CDK7 - Q901 | I/II | Recruiting | NCT05394103 |
| **72** | A Phase 1/2, Open-Label Study to Evaluate the Safety, Tolerability, Pharmacokinetics, and Efficacy of INX-315 in pts With Advanced Cancer | **Arm A.** Multiple doses of INX-315 monotherapy, oral administration. **Arm B.** INX-315 monotherapy, oral administration. **Arm C.** INX-315 in combination with CDK4/6i and endocrine therapy, oral administration | CDK2 - INX-315 | I/II | Recruiting | NCT05735080 |
| **73** | A Phase 1 Open-Label, Multi-Center, Safety and Efficacy Study of PRT2527 as Monotherapy and in Combination With Zanubrutinib in Participants With Relapsed/Refractory Hematologic Malignancies | **Arm A.** PRT2527 will be administered by intravenous infusion once weekly on a 21-day treatment cycle at the dose level assigned during the dose escalation phase and at the defined RP2D dose for indication-specific cohorts during the dose confirmation phase. **Arm B.** PRT2527 will be administered by intravenous infusion once weekly on a 35-day treatment cycle for Cycle 1 followed by 21-day treatment for subsequent treatment cycles at the dose level assigned during the dose escalation phase and at the defined RP2D dose for indication specific cohort during the dose confirmation phase. **Arm C.** Zanubrutinib will be administered orally as combination therapy once daily. | CDK9 - PRT2527 | I | Recruiting | NCT05665530 |
| **74** | A Phase I Clinical Study of Safety, Tolerability, Pharmacokinetics, and Initial Efficacy of ETH-155008 Tablets in pts With Relapsed or Refractory Acute Myeloid Leukemia and Non-Hodgkin's Lymphoma | **Arm A.** Dose level: 20mg/day, 40mg/day, 60mg/day, 80mg/day, 100mg/day. Each dose level will recruit 1-6 subjects, taking ETH-155008 tablets once daily. Intervention: Drug: ETH-155008 | CDK4/6 - ETH-155008 | I | Recruiting | NCT05758610 |
| **75** | Precision Treatment of Refractory Triple Negative Breast Cancer Based on Molecular Subtyping --FUSCC-TNBC- Umbrella Trial | **Arm A.** Pyrotinib with Capecitabine, if patients were LAR subtype with HER2 gene activated mutation. **Arm B.** AR inhibitor with CDK4/6 inhibitor. If patients were LAR subtype without HER2 gene activated mutation, but had PIK3CA mutation, enter into arm B1; If patients were LAR subtype without HER2 gene activated mutation or PIK3CA mutation, enter into arm B2;If B2 was closed, enter into B4. **Arm C.** Anti PD-1 with nab-paclitaxel. If patients were IM subtype(CD8 positive T cell more than 20%). **Arm D.** PARP inhibitor included therapy. If patients were BLIS subtype and had a BRCA gene pathogenic mutation. **Arm E.** BLIS with anti-VEGFR included therapy. If patients were BLIS subtype and did not have a BRCA gene pathogenic mutation. **Arm F.** MES with anti-VEGFR included therapy. If patients were MES subtype and without PI3K/AKT pathway activation. **Arm G.** mTOR inhibitor with nab-paclitaxel. If patients were MES subtype and had PI3K/AKT pathway activation. | CDK4/6 - SHR6390 | I/II | Recruiting | NCT03805399 |
| **76** | Combination Followed by Maintenance Chemotherapy Versus CDK4/6 Inhibitor Combined With Endocrine Therapy for HR Low/HER2-negative Advanced Breast Cancer: a Prospective, Randomized, Open-label Phase Ⅱ Clinical Trial | **Arm A.** Nab-paclitaxel 130mg/m2, day 1 and day 8 Capecitabine 2000mg/m2, from day1 to DAY 14 Vinorelbine 25mg/m2, day 1 and day 8 **Arm B.** Palbociclib/Dalpiciclib Letrozole/Anastrozole/fulvestrant | CDK4/6 - Dalpiciclib | II | Not yet recruiting | NCT06176534 |
| **77** | Cyclin dEpendent Kinase in tRiple nEGatIVe brEast canceR - a "Window of Opportunity" Study | **Arm A.** Palbociclib alone (125 mg orally (PO) per day, days 1-14) + Paclitaxel alone (80 mg/m^2 intravenously (IV), day 1, 8, 15 and 22) **Arm B.** Paclitaxel (80 mg/m^2 IV, day 1, 8, 15 and 22) + Palbociclib (125 mg PO per day, days 1-21) **Arm C.** Carboplatin alone (area under the curve (AUC) 2 IV, day 1, 8, 15 and 22)+ Carboplatin (AUC 2 IV, day 1, 8, 15 and 22) + Palbociclib (125 mg PO per day, days 1-21) | CDK4/6-Palbociclib | II | Not yet recruiting | NCT05067530 |
| **78** | A Phase 2 Multicenter, Double-blind, Randomized-controlled Study of Abemaciclib (CDK4 and 6 Inhibitor) in Newly Diagnosed RB-proficient Grade 3 Meningioma Participants | **Arm A.** Palbociclib administered twice daily on days 1-28 of each 28-day cycle. **Arm B.** Placebo administered twice daily on days 1-28 of each 28-day cycle. | CDK4/6 - Abemaciclib | II | Not yet recruiting | NCT05940493 |
| **79** | An Exploratory Clinical Study of CDK4/6 Inhibitor Dalpiciclib Combined With Letrozole in Neoadjuvant Treatment of Stage Ⅱ-Ⅲ HR-positive/HER2-negative Breast Cancer | Dalpiciclib: 150 mg (p.o.) was given once daily for 3 weeks, followed by 1 week off in each 4-week cycle + Letrozole: 2.5mg, p.o., once a day, continuous administration. 28 days as one cycle. | CDK4/6 - Dalpiciclib | II | Not yet recruiting | NCT05512780 |
| **80** | Exploration of Dalpiciclib + Chidamide in HR+/HER2- Advanced Breast Cancer After Failure of CDK4/6 Inhibitor: a Phase Ⅰb Study | **Arm A.** Dalpiciclib: 100 mg/d or 125 mg/d, po., qd, administered on an empty stomach (fasting should be ensured at least 1 hour before and 1 hour after administration). The drug will be administered in a 28-day cycle, with continuously administration in the first 3 weeks (D1-21), and discontinuation in the fourth week (D22-28). + Chidamide: 25 mg/BIW or 20 mg/BIW, po., q2w. The interval between doses should not be less than 3 days (e.g. Monday and Thursday, Tuesday and Friday, Wednesday and Saturday, etc.), administered 30 minutes after meals | CDK4/6 - Dalpiciclib | I | Not yet recruiting | NCT05586841 |
| **81** | A Phase II, Single Arm, Open-label Trial of Dalpiciclib Plus Fulvestrant With Pyrotinib in Hormone Receptor-positive, HER2-low Advanced Breast Cancer That Progressed on Previous CDK4/6i Plus AI Therapy | **Arm A.** Dalpiciclib 125 mg/day orally continuously dosed for 3 weeks followed by 1 week off + Pyrotinib 320mg/day orally continuously + Fulvestrant 500mg intramuscularly on Days 1 and 15 of Cycle 1, and then on Day 1 of each subsequent 28 day cycle | CDK4/6 - Dalpiciclib | II | Not yet recruiting | NCT05806671 |
| **82** | A Phase II, Open-label, Multicenter Study of Orally Administered RVU120 for the Treatment of Anemia in pts With Lower-risk Myelodysplastic Neoplasms (MDS) | **Arm A.** RVU120 will be taken orally every other day (q.o.d). in a 21-day treatment cycle. Doses of RVU120 will be administered from day 1 to day 13 (total of 7 doses per cycle). | CDK8/19 - RVU120 | II | Not yet recruiting | NCT06243458 |
| **83** | Phase I Study of the CDK4/6 Inhibitor Palbociclib (PD-0332991) in Combination With the MEK Inhibitor Binimetinib (MEK162) for pts With Advanced KRAS Mutant Non-Small Cell Lung Cancer | **Arm A.** Palbociclib will be administered orally once daily (patients will be dosed with palbociclib for three weeks out of every four weeks per cycle). Binimetinib will be administered orally twice daily (continuously through the four weeks per cycle) | CDK4/6-Palbociclib | I | Active (not recruiting) | NCT03170206 |
| **84** | A MULTICENTER, RANDOMIZED, DOUBLE-BLIND PHASE 3 STUDY OF Palbociclib (ORAL CDK 4/6 INHIBITOR) PLUS LETROZOLE VERSUS PLACEBO PLUS LETROZOLE FOR THE TREATMENT OF PREVIOUSLY UNTREATED ASIAN POSTMENOPAUSAL WOMEN WITH ER (+), HER2 (-) ADVANCED BREAST CANCER | **Arm A.** Palbociclib, 125mg, orally once daily on Day 1 to Day 21 of every 28-day cycle followed by 7 days off treatment in combination with Letrozole, 2.5mg, orally once daily (continuously) + Placebo, 125mg, orally once daily on Day 1 to Day 21 of every 28-day cycle followed by 7 days off treatment in combination with Letrozole, 2.5mg, orally once daily (continuously). | CDK4/6-Palbociclib | III | Active (not recruiting) | NCT02297438 |
| **85** | PREDIX Luminal B - Neoadjuvant Response-guided Treatment of ER Positive Tumors With High Proliferation or Low Proliferation With Metastatic Nodes. Part of a Platform of Translational Phase II Trials Based on Molecular Subtypes | **Arm A.** Pts receive weekly paclitaxel 80mg/m2, eventually dose-adjusted in relation to side effects, for a 12-week period. Thereafter, treatment is switched to endocrine treatment in combination with palbociclib. Pre- or perimenopausal women and all men are treated with tamoxifen, alternatively with an LHRH analogue in combination with an aromatase inhibitor (only women); postmenopausal women receive an aromatase inhibitor together with palbociclib 125 mg orally days 1-21, followed by a 7-days rest period, repeated twice during the second 12-week period. **Arm B.** Pre- or perimenopausal women and all men are treated with tamoxifen together with palbociclib 125 mg orally days 1-21, followed by a 7-days rest period, repeated twice during a 12-week period. Thereafter, treatment is switched to weekly paclitaxel 80mg/m2, eventually dose-adjusted in relation to side effects, for further 12 weeks. **Arm C.** Postmenopausal women receive an aromatase inhibitor together with palbociclib 125 mg orally days 1-21, followed by a 7-days rest period, repeated twice during a 12-week period. Thereafter, treatment is switched to weekly paclitaxel 80mg/m2, eventually dose-adjusted in relation to side effects, for further 12 weeks. **Arm D.** Pre- or perimenopausal women may be treated with goserelin and an aromatase inhibitor together with palbociclib 125 mg orally days 1-21, followed by a 7-days rest period, repeated twice during a 12-week period. Thereafter, treatment is switched to weekly paclitaxel 80mg/m2, eventually dose-adjusted in relation to side effects, for further 12 weeks. | CDK4/6-Palbociclib | II | Active (not recruiting) | NCT02603679 |
| **86** | PREDIX Luminal A - Neoadjuvant Response-guided Treatment of Slowly Proliferating Hormone Receptor Positive Tumors. Part of a Platform of Translational Phase II Trials Based on Molecular Subtypes | **Arm A.** Pre- or perimenopausal women are treated with tamoxifen, alternatively with an LHRH analogue in combination with an aromatase inhibitor (only women); postmenopausal women receive an aromatase inhibitor. The preoperative treatment is continued for further 12 weeks, provided that re-evaluation after 6 weeks, week 10 of the preoperative treatment, does not indicate progression. Upon progression (PD), individualized management, preferentially surgery, is the primary option. **Arm B.** Pts receive the same endocrine treatment as in arm A together with palbociclib 125 mg orally days 1-21, followed by a 7-days rest period. The combined treatment is continued for further 12 weeks, provided that re-evaluation after 6 weeks, week 10 of the preoperative treatment, does not indicate progression. Upon progression (PD), individualized management, preferentially surgery, is the primary option. **Arm C.** Pts receive the same endocrine treatment as in arm A together with palbociclib 125 mg orally days 1-21, followed by a 7-days rest period. The combined treatment is continued for further 12 weeks, if re-evaluation after 6 weeks, week 10 of the preoperative treatment, does not indicate progression. Upon progression (PD), individualized management, preferentially surgery, is the primary option. | CDK4/6-Palbociclib | II | Active (not recruiting) | NCT02592083 |
| **87** | BOOG 2017-03: Endocrine Therapy Plus CDK 4/6 Inhibition in First- or Second-line for Hormone Receptor Positive Advanced Breast Cancer - the SONIA Study | **Arm A. Strategy A CDK4/6 inhibitor in 1st line.** Non-steroidal aromatase inhibitor (letrozole or anastrozole, at the discretion of the treating physician) plus CDK4/6 inhibitor (palbociclib, ribociclib or abemaciclib, depending on availability and physician's preference) in first line followed by fulvestrant in second line. **Arm B. Strategy B CDK4/6 inhibitor in 2nd line.** Non-steroidal aromatase inhibitor (letrozole or anastrozole, at the discretion of the treating physician) in first line followed by fulvestrant plus CDK4/6 inhibitor in second line (palbociclib, ribociclib or abemaciclib, depending on availability and physician's preference). | CDK4/6-Palbociclib/ Abemaciclib/ Ribociclib | III | Active (not recruiting) | NCT03425838 |
| **88** | A Phase I Trial of Palbociclib and Bosutinib With Fulvestrant in pts With Metastatic Hormone Receptor Positive and HER2 Negative (HR+ HER2-) Breast Cancer Refractory to an Aromatase Inhibitor and a CDK4/6 Inhibitor (ASPIRE - WI231696) | **Arm A.** Palbociclib (75mg daily for 21 days of each 28 day cycle) + Bosutinib (300mg on days 1-5 of each week of the 28 day cycle) + Fulvestrant (500mg on days day 1, 5, and 28 of each 28 day cycle). **Arm B.** Palbociclib (75mg daily for the first 21 days of each 28 day cycle) + Bosutinib (300mg on days 1-5 of each week of the 28 day cycle). **Arm C.** Palbociclib (100mg daily for 21 days of each 28 day cycle) + Bosutinib (300mg on days 1-5 of each week of the 28 day cycle) + Fulvestrant (500mg on days day 1, 5, and 28 of each 28 day cycle). **Arm D.** Palbociclib (100mg daily for 21 days of each 28 day cycle) + Bosutinib (500mg on days 1-5 of each week of the 28 day cycle) + Fulvestrant (500mg on days day 1, 5, and 28 of each 28 day cycle). | CDK4/6-Palbociclib | I | Active (not recruiting) | NCT03854903 |
| **89** | A Phase I/II Dose Escalation Study of the CDK4/6 Inhibitor, Palbociclib in Combination With Cetuximab and Intensity Modulated Radiation Therapy (IMRT) for Locally Advanced Squamous Cell Carcinoma of the Head and Neck | **Arm A.** IMRT (5 days on/2 days off with a total dose of 70 Gy for 33-35 fractions) + Cetuximab (400 mg/m2 IV at 7 days before (day -7) starting radiation and then 250 mg/m2 IV weekly for 7 weeks) + Palbociclib (orally daily 3 week-on and 1-week of during IMRT (Day 1-21 and Day 29-49) on 3 dose levels and the MTD) | CDK4/6-Palbociclib | I/II | Active (not recruiting) | NCT03024489 |
| **90** | A Phase Ib Trial of Fulvestrant, Palbociclib (CDK4/6 Inhibitor) and Erdafitinib (JNJ- 42756493,Pan-FGFR Tyrosine Kinase Inhibitor) in ER+/HER2-/FGFR-Amplified Metastatic Breast Cancer (MBC) | **Arm A. Escalation.** Fulvestrant - injection into muscle 1 time per month. Palbociclib capsule taken by mouth 1 time per day every 21 days followed by 1 week of rest (no drug taken). Erdafitinib tablet taken by mouth 1 time per day. **Arm B. Expansion.** Fulvestrant - injection into muscle 1 time per month. Palbociclib capsule taken by mouth 1 time per day every 21 days followed by 1 week of rest (no drug taken). Erdafitinib tablet taken by mouth 1 time per day | CDK4/6-Palbociclib | I | Active (not recruiting) | NCT03238196 |
| **91** | A Phase 1 Study Of Palbociclib (IND#141416), A CDK 4/6 Inhibitor, In Combination With Chemotherapy In Children With Relapsed Acute Lymphoblastic Leukemia (ALL) Or Lymphoblastic Lymphoma (LL) | **Arm A.** Pts receive Palbociclib 50 mg/m^2 (starting dose with maximum dose of 100 mg) PO (or via NG-tube) once daily on Days 1-21; Intrathecal cytarabine (IT ARAC) age-based dosing on Day 1, Doxorubicin 60 mg/m^2 IV push or infusion over 1-15 min on Day 4; Prednisone or prednisolone 40 mg/m^2 PO divided BID or TID on days 4-31; Vincristine 1.5 mg/m^2 (maximum dose 2 mg) IV push or mini-bag per institutional policy on days 4, 11, 18, and 25; and Pegaspargase 2500 IU/m^2 IV over 1-2 hours on Days 5, and 18. If CNS3 leukemia is present, pts receive Intrathecal Triple Therapy (ITT) age-based dosing on days 4, 11, 18, and 25. pts known to be CNS3 at study entry may receive ITT on Day 1 rather than IT ARAC. If CNS1 and 2 leukemia present, patient receive Methotrexate (IT MTX) age-based dosing on Days 18 and 32. Treatment will be given for one cycle, 32 days, in the absence of disease progression or unacceptable toxicity. | CDK4/6-Palbociclib | I | Active (not recruiting) | NCT03792256 |
| **92** | A Phase II Study of Palbociclib, A CDK4/6 Inhibitor, in pts With Metastatic Castration-Resistant Prostate Cancer | **Arm A.** Palbociclib (125mg orally days 1-21 every 28 day cycle) | CDK4/6-Palbociclib | II | Active (not recruiting) | NCT02905318 |
| **93** | Combination of MEK Inhibitor Binimetinib and CDK4/6 Inhibitor Palbociclib in KRAS and NRAS Mutant Metastatic Colorectal Cancers | **Arm A.** Pts receive binimetinib PO BID on days 1-28 and palbociclib PO QD on days 1-21. Treatment repeats every 28 days for up to 24 cycles in the absence of disease progression or unacceptable toxicity. **Arm B**. Pts receive trifluridine and tipiracil hydrochloride PO BID on days 1-5 and 8-12. Treatment repeats every 28 days for up to 24 cycles in the absence of disease progression or unacceptable toxicity. Patients with disease progression may optionally crossover to Arm A. | CDK4/6-Palbociclib | II | Active (not recruiting) | NCT03981614 |
| **94** | A PHASE 1 OPEN-LABEL PHARMACOKINETICS STUDY OF Palbociclib, A CYCLIN-DEPENDENT KINASE 4 AND 6 (CDK4/6) INHIBITOR, IN POSTMENOPAUSAL CHINESE WOMEN WITH ER (+), HER2 (-) ADVANCED BREAST CANCER | **Arm A.** Palbociclib (125 mg orally once daily with food on Day 1 to Day 21 followed by 7 days off treatment in a 28-day cycle9 + Letrozole (2.5 mg, orally once daily continuously) | CDK4/6-Palbociclib | I | Active (not recruiting) | NCT02499146 |
| **95** | A Phase II Study of Adjuvant Palbociclib as an Alternative to CHemotherapy in Elderly pts With High-risk ER+/HER2- Early Breast Cancer | **Arm A.** Standard adjuvant endocrine therapy for a duration of at least 5 years + palbociclib (one capsule 125mg QD, orally, for 21 days followed by 7 days off treatment) for a total duration of up to 2 years. **Arm B.** Adjuvant chemotherapy: 4 cycles docetaxel (75 mg/m2) + cyclophosphamide (600 mg/m2 q3w) or 4 cycles doxorubicin (60 mg/m2) + cyclophosphamide (600 mg/m2 q3w) or 4 cycles epirubicin (90 mg/m2) + cyclophosphamide (600 mg/m2 q3w) or 4 cycles weekly paclitaxel (80 mg/m2 D1, D8, and D15 q3w). Followed by standard adjuvant endocrine therapy for a duration of at least 5 years. | CDK4/6-Palbociclib | II | Active (not recruiting) | NCT03609047 |
| **96** | SERENA-4: A Randomised, Multicentre, Double-Blind, Phase III Study of AZD9833 (an Oral SERD) Plus Palbociclib Versus Anastrozole Plus Palbociclib for the Treatment of pts With Estrogen Receptor-Positive, HER2-Negative Advanced Breast Cancer Who Have Not Received Any Systemic Treatment for Advanced Disease | **Arm A.** Pts will receive AZD9833 (75 mg, PO, once daily) + palbociclib (PO, once daily, 125 mg for 21 consecutive days followed by 7 days off treatment) + anastrozole placebo (1 mg, PO, once daily). **Arm B.** Pts will recieve Anastrozole (1 mg, PO, once daily) + palbociclib (PO, once daily, 125 mg for 21 consecutive days followed by 7 days off treatment) + AZD9833 placebo (PO, once daily). | CDK4/6-Palbociclib | III | Active (not recruiting) | NCT04711252 |
| **97** | A Phase 1/1b Trial of MRTX849 in Combination With Palbociclib in pts With Advanced Solid Tumors With KRAS G12C Mutation | **Arm A. Dose escalation.** MRTX849 and palbociclib to determine maximum tolerated dose in combination. **Arm B. Dose Expansion.** Expansion cohorts may be implemented to ensure sufficient safety experience, pharmacokinetic data and early evidence of clinical activity of MRTX in combination with palbociclib. | CDK4/6-Palbociclib | I | Active (not recruiting) | NCT05178888 |
| **98** | A randoMized phAse II trIal of fulvestraNt wiTh or Without Ribociclib After Progression on AntI-estrogeN Therapy Plus Cyclin-dependent Kinase 4/6 Inhibition in pts With Unresectable or Metastatic Hormone Receptor +, HER2 - Breast Cancer (MAINTAIN Trial) | **Arm A.** LEE-011 (orally, 600mg, daily for 3 weeks and 1 week off) + Fulvestrant (intramuscularly, 500mg, every 2 weeks x 3, then every 4 weeks). **Arm B.** Placebo (orally, 600mg, daily for 3 weeks and 1 week off) + Fulvestrant (intramuscularly, 500mg, every 2 weeks x 3, then every 4 weeks). | CDK4/6 - Ribociclib | II | Active (not recruiting) | NCT02632045 |
| **99** | Ribociclib and Spartalizumab for Head and Neck Squamous Cell Carcinoma, a Phase I Study With Expansion Cohort (RISE-HN) | **Arm A.** Ribociclib (400mg, 600mg, or 200mg oral daily, D1-D21, 28 days a cycle) + Spartalizumab (400mg ivdrip on D1, 28 days a cycle). | CDK4/6 - Abemaciclib | I | Active (not recruiting) | NCT04213404 |
| **100** | A Randomized, Double-Blind, Placebo-Controlled, Phase 3 Study of Nonsteroidal Aromatase Inhibitors (Anastrozole or Letrozole) Plus LY2835219, a CDK4/6 Inhibitor, or Placebo in Postmenopausal Women With Hormone Receptor-Positive, HER2-Negative Locoregionally Recurrent or Metastatic Breast Cancer With No Prior Systemic Therapy in This Disease Setting | **Arm A.** Abemaciclib orally every 12 hours plus either 1 mg anastrozole or 2.5 mg letrozole orally once daily for 28 days (28 day cycles). **Arm B.** Placebo orally every 12 hours plus either 1 mg anastrozole or 2.5 mg letrozole orally once daily for 28 days (28 day cycles). | CDK4/6- Abemaciclib | III | Active (not recruiting) | NCT02246621 |
| **101** | postMONARCH: A Randomized, Double Blind, Placebo-Controlled, Phase 3 Study to Compare the Efficacy of Abemaciclib Plus Fulvestrant to Placebo Plus Fulvestrant in Participants With HR+, HER2-, Advanced or Metastatic Breast Cancer Following Progression on a CDK4 & 6 Inhibitor and Endocrine Therapy | **Arm A.** Abemaciclib administered orally in combination with fulvestrant administered intramuscularly (IM). **Arm B.** Placebo administered orally in combination with fulvestrant administered IM. | CDK4/6- Abemaciclib | III | Active (not recruiting) | NCT05169567 (post MONARCH) |
| **102** | A Randomized, Double-Blind, Placebo-Controlled, Phase 3 Study to Compare NSAI (Anastrozole or Letrozole) Plus Abemaciclib, a CDK4 and CDK6 Inhibitor, or Plus Placebo, and to Compare Fulvestrant Plus Abemaciclib or Plus Placebo in Postmenopausal Women With Hormone Receptor-Positive, HER2-Negative Locoregionally Recurrent or Metastatic Breast Cancer | **Arm A.** Abemaciclib given orally every 12 hours (Q12H) plus anastrozole or letrozole given orally every 24 hours (Q24H) on days 1 to 28 of a 28 day cycle. Participants receiving benefit may continue until disease progression. **Arm B.** Placebo given orally Q12H plus anastrozole or letrozole given orally Q24H on days 1 to 28 of a 28 day cycle. Participants receiving benefit may continue until disease progression. **Arm C.** Abemaciclib given orally Q12H on days 1 to 28 of a 28 day cycle plus fulvestrant intramuscularly (IM) on days 1 and 15 of cycle 1, then on day 1 of cycle 2 and beyond. Participants receiving benefit may continue until disease progression. **Arm D.** Placebo given orally Q12H on days 1 to 28 of a 28 day cycle plus fulvestrant IM on days 1 and 15 of cycle 1, then on day 1 of cycle 2 and beyond. Participants receiving benefit may continue until disease progression. | CDK4/6 - Abemaciclib | III | Active (not recruiting) | NCT02763566 (MONARCH plus) |
| **103** | Evaluation of the Effects of Endocrine Therapy and Abemaciclib on Host and Tumor Immune Cell Repertoire/Function in Advanced ER+/HER2- Breast Cancer | **Arm A.** Abemaciclib (50 - 150mg tablet BID as prescribed per standard of care) + Fulvestrant (500mg as prescribed per standard of care). **Arm B.** Abemaciclib (50 - 150mg tablet BID as prescribed per standard of care) + Aromatase Inhibitors (Letrozole, anastrozole as prescribed per standard of care) | CDK4/6 - Abemaciclib | II | Active (not recruiting) | NCT04352777 |
| **104** | An Open-label, Multi-center Phase IB/II Study of Abemaciclib With Paclitaxel for CDK4/6 Pathway Activated Tumors | **Arm A.** Abemaciclib + Paclitaxel. Phase 1b: About 3-6 patients enrollment is expected at dose level 1, -1 and -2. Dose reduction will be preceded with 3 patients/cohort until the first DLT with 4 weeks' observation. The recommend phase 2 dose (RP2D) will be defined. Level1: abemaciclib (100mg bid, D1-28) + Paclitaxel (80mg/m2, IV. D1, 8, 15 Q 4 weeks). Level-1: Abemaciclib (100mg bid, D1-28) + Paclitaxel (70mg/m2, IV. D1, 8, 15 Q 4 weeks). Level-2: Abemaciclib (50mg bid, D1-28) + Paclitaxel (70mg/m2, IV. D1, 8, 15 Q 4 weeks). Phase2: At the RP2D dose level in phase I part, we will expand phase 2 study. | CDK4/6-Abemaciclib | I/II | Active (not recruiting) | NCT04594005 |
| **105** | MONARCH 2: A Randomized, Double-Blind, Placebo-Controlled, Phase 3 Study of Fulvestrant With or Without Abemaciclib, a CDK4/6 Inhibitor, for Women With Hormone Receptor Positive, HER2 Negative Locally Advanced or Metastatic Breast Cancer | **Arm A.** Abemaciclib (150 milligram mg, orally every 12 hours on Days 1 to 28 of a 28-day cycle) + Fulvestrant (500mg intramuscularly (IM) on Days 1 and 15 of Cycle 1, then on Day 1 of Cycle 2 and beyond). Participants received treatment until discontinuation were met. **Arm B.** Placebo (orally every 12 hours on Days 1 to 28 of a 28-day cycle) + Fulvestrant (500mg IM on Days 1 and 15 of Cycle 1, then on Day 1 of Cycle 2 and beyond). Participants received treatment until discontinuation were met. | CDK4/6- Abemaciclib | III | Active (not recruiting) | NCT02107703 |
| **106** | A Phase II Trial of Osimertinib and Abemaciclib With a Focus on Non-Small Cell Lung Cancer pts With EGFR Activating Mutations With Osimertinib Resistance | **Arm A.** Osimertinib 80 mg QD Abemaciclib 150mg BID. | CDK4/6- Abemaciclib | II | Active (not recruiting) | NCT04545710 |
| **107** | A Phase 1 Trial of the Combination of the Heat Shock Protein-90 (HSP90) Inhibitor Onalespib (AT13387) and the Cyclin-Dependent Kinase (CDK) Inhibitor AT7519M in pts With Advanced Solid Tumors | **Arm A.** Pts receive Onalespib (IV) over 1 hour on days 1 and 4 (cycle 0 only). Pts then receive Onalespib (IV) and CDKI AT7519 (IV) on days 1, 4, 8, and 11 (cycle 1 and subsequent cycles thereafter). Cycles repeat every 21 days (7 days for course 0 only) in the absence of disease progression or unacceptable toxicity. | Pan CDK - AT7519 | I | Active (not recruiting) | NCT02503709 |
| **108** | Pyrotinib Maleate, CDK4/6 Inhibitor and Letrozole in Combination for Treatment of Stage II-III Triple-positive Breast Cancer: a Phase II Clinical Trial | **Arm A.** Pyrotinib maleate + CDK4/6 inhibitor SHR6390 + Letrozole. The effectiveness of the combined treatment will be evaluated by MRI every two treatment cycles. If the disease progresses, the participant will withdraw from the trial. If the combined treatment has identified effectiveness, the participant will undergo surgical treatment within 4 weeks (over 2 weeks) after termination of the neoadjuvant treatment. The patients will be followed up for 5 years. | CDK4/6 - SHR6390 | II | Active (not recruiting) | NCT04486911 |
| **109** | Study to Evaluate the Efficacy and Safety of CDK4/6 Inhibitor SHR6390 Combined With Pyrotinib in the Treatment of HER2-positive Advanced Breast Cancer | **Arm A.** Pts with HER2 positive breast cancer will receive Pyrotinib in combination with SHR6390 (at protocol defined dose levels) orally until disease progression, unacceptable toxicity, withdrawal of consent or death, whichever occurs first. | CDK4/6 - SHR6390 | II | Active (not recruiting) | NCT04293276 |
| **110** | A Phase II Trial of Neoadjuvant PD 0332991, a Cyclin-Dependent Kinase (Cdk) 4/6 Inhibitor, in Combination With Anastrozole in Women With Clinical Stage 2 or 3 Estrogen Receptor Positive and HER2 Negative Breast Cancer | **Arm A.** **PIK3CA Wild Type Cohort. Arm B. PIK3CA Mutant Type Cohort. Arm C. Endocrine resistant Cohort.** Tumor biopsy for testing/research at baseline and Cycle 1 Day 15. Cycle 0 is 28 days of anastrozole PO daily and, if premenopausal, goserelin SC every 28 days. Cycles 1-5: PD 0332991 combined with aanastrozole (and Goserelin if premenopausal) is to be (4) 28-day cycles followed by a 5th cycle of 10-12 days duration consisting of daily PD 0332991 and Anastrozole (last dose day before surgery). Standard surgery will be performed per institutional standards 2-4 weeks following the completion of Cycle 4 in those who did not receive Cycle 5. In pts who receive Cycle 5, surgery occurs on Day 11, 12, or 13 of Cycle 5. Pts who derived benefit from the therapy have the option of taking PD 0332991 in combination with endocrine therapy for 23 cycles after surgery and adjuvant chemotherapy and radiation if indicated. It should be re-started at least 4 weeks after the completion of chemotherapy and radiation therapy if these treatments were planned. | CDK4/6 - PD 0332991 | II | Active (not recruiting) | NCT01723774 |
| **111** | Phase 1/2 Safety, Pharmacokinetic, and Antitumor Activity Study of G1T38 in Combination With Fulvestrant in pts With Hormone Receptor-Positive, HER2-Negative Locally Advanced or Metastatic Breast Cancer After Endocrine Failure | **Arm A.** G1T38 (Lerociclib) orally (once daily) in combination with Fulvestrant.**Arm B.** G1T38 (Lerociclib) orally (twice daily) in combination with Fulvestrant. | CDK4/6 - G1T38 | I/II | Active (not recruiting) | NCT02983071 |
| **112** | A Phase 3, Randomized, Double-Blind Study of Trilaciclib or Placebo in pts Receiving First- or Second-Line Gemcitabine and Carboplatin Chemotherapy for Locally Advanced Unresectable or Metastatic Triple-Negative Breast Cancer (PRESERVE 2) | **Arm A.** Trilaciclib (240mg/m2) + Gemcitabine (1000 mg/m2) and Carboplatin (AUC 2). **Arm B.** Placebo (240mg/m2) + Gemcitabine (1000 mg/m2) and Carboplatin (AUC 2) | CDK4/6- Trilaciclib | III | Active (not recruiting) | NCT04799249 |
| **113** | Trilaciclib Administered Prior to Sacituzumab Govitecan-hziy in pts With Unresectable Locally Advanced or Metastatic Triple-Negative Breast Cancer Who Received at Least Two Prior Treatments, at Least One in the Metastatic Setting | **Arm A.** Trilaciclib + Sacituzumab govitecan-hziy on days 1 & 8 of a 21 day cycle. Trilaciclib is administered first, followed by sacituzumab govitecan-hziy. | CDK4/6- Trilaciclib | II | Active (not recruiting) | NCT05113966 |
| **114** | A Phase 1 Open-Label, Multi-Center, Safety and Efficacy Study of PRT3645 in Participants With Select Advanced or Metastatic Solid Tumors | **Arm A.** PRT3645 capsules will be self-administered once daily, continuously, at the dose-level assigned | CDK4/6- PRT3645 | I | Active (not recruiting) | NCT05538572 |
| **115** | A Phase II Trial of SCH 727965 (NSC 747135) in pts With Stage IV Melanoma | **Arm A.** Pts receive Dinaciclib IV over 2 hours on day 1. Treatment repeats every 21 days in the absence of disease progression or unacceptable toxicity. | CDK1/2/5/9- Dinaciclib | II | Active (not recruiting) | NCT00937937 |
| **116** | Phase 1 Trial of ABT-888 and SCH727965 in pts With Advanced Solid Tumors | **Arm A.** PART 1A: pts receive Veliparib (PO BID on days 1-28) and Dinaciclib (IV over 2 hours on days 8 and 22). Cycles repeat every 28 days in the absence of disease progression or unacceptable toxicity. PART 1B: pts receive Veliparib and Dinaciclib as pts in Part 1A. Cycles repeat every 28 days in the absence of disease progression or unacceptable toxicity. PART 1C: pts receive Veliparib (PO BID on days 1-7 of cycle 0). Pts then receive Veliparib (PO BID on days 1-21) and Dinaciclib (IV over 2 hours on days 1, 4, 8, and 11 or days 1 and 8). Cycles repeat every 21 days in the absence of disease progression or unacceptable toxicity. | CDK1/2/5/9- Dinaciclib | I | Active (not recruiting) | NCT01434316 |
| **117** | A Phase Ib Study of RVU120 (SEL120) in pts With Acute Myeloid Leukemia or High-risk Myelodysplastic Syndrome | **Arm A.** RVU120 (SEL120). The first part of the study consists of dose-escalation cohorts where patients will receive ascending doses of RVU120 (SEL120) to determine the recommended dose (RD) for further clinical development. The second part of the study is an enrichment cohort where additional 6 to 20 patients will be treated with RVU120 (SEL120) to support the evaluation of the RD. | CDK8/19- RVU120 | I | Active (not recruiting) | NCT04021368 |
| **118** | A Phase Ib/II, Multicenter Study of the Combination of LEE011 and BYL719 With Letrozole in Adult pts With Advanced ER+ Breast Cancer | **Arm A.** LEE011 - 28 day cycles (21 days followed by a 7 day break - dose escalating), letrozole - 2.5 mg/day. **Arm B.** BYL719 - daily (dose escalating) letrozole - 2.5 mg/day. **Arm C.** LEE011 - 28 day cycles (21 days followed by a 7 day break -dose escalating), BYL719 - daily (dose escalating), letrozole 2.5 mg/day. **Arm D.** LEE011-daily (dose escalating), BYL719 -daily (dose escalating), letrozole 2.5 mg/day | CDK4/6- Ribociclib | I/II | Active (not recruiting) | NCT01872260 |
| **119** | Pyrotinib, Dalpiciclib (SHR6390) and Endocrine Therapy in Subjects With Dual-receptor Positive (ER+/HER2+) Advanced Breast Cancer: a Multi-center Phase Ib/II Study | **Arm A.** Pyrotinib 320mg/d, SHR6390 125mg/d, and letrozole 2.5mg/d was declared as RP2D. The pharmacokinetic analysis had not yielded conclusive results and would involve more samples in phase II trial. Dalpiciclib combined with Pyrotinib and Endocrine therapy (treatment of physician's choice: letrozole or fulvestrant) ER+/HER2+ metastatic breast cancer pts eligible for first- or second-line treatment were enrolled to receive dalpiciclib combined with pyrotinib and endocrine therapy (treatment of physician's choice: letrozole or fulvestrant) | CDK4/6-Dalpiciclib | I/II | Active (not recruiting) | NCT03772353 |

The table reports a complete list of interventional clinical trials recruiting, active not recruiting and not yet recruiting using CDKs inhibitors as obtained by searching the <https://www.clinicaltrials.gov/> database on may 2024.
